# Current methods to analyze lysosome morphology, positioning, motility and function

**DOI:** 10.1111/tra.12839

**Published:** 2022-04-24

**Authors:** Duarte C. Barral, Leopoldo Staiano, Cláudia Guimas Almeida, Dan F. Cutler, Emily R. Eden, Clare E. Futter, Antony Galione, André R. A. Marques, Diego Luis Medina, Gennaro Napolitano, Carmine Settembre, Otília V. Vieira, Johannes M. F. G. Aerts, Peace Atakpa‐Adaji, Gemma Bruno, Antonella Capuozzo, Elvira De Leonibus, Chiara Di Malta, Cristina Escrevente, Alessandra Esposito, Paolo Grumati, Michael J. Hall, Rita O. Teodoro, Susana S. Lopes, J. Paul Luzio, Jlenia Monfregola, Sandro Montefusco, Frances M. Platt, Roman Polishchuck, Maria De Risi, Irene Sambri, Chiara Soldati, Miguel C. Seabra

**Affiliations:** ^1^ CEDOC, NOVA Medical School, NMS, Universidade NOVA de Lisboa Lisbon Portugal; ^2^ Telethon Institute of Genetics and Medicine (TIGEM) Pozzuoli Italy; ^3^ Institute for Genetic and Biomedical Research National Research Council (CNR) Milan Italy; ^4^ MRC Laboratory for Molecular Cell Biology University College London London UK; ^5^ University College London (UCL) Institute of Ophthalmology London UK; ^6^ Department of Pharmacology University of Oxford Oxford UK; ^7^ Medical Genetics Unit, Department of Medical and Translational Science Federico II University Naples Italy; ^8^ Clinical Medicine and Surgery Department Federico II University Naples Italy; ^9^ Leiden Institute of Chemistry Leiden University Leiden The Netherlands; ^10^ Department of Pharmacology University of Cambridge Cambridge UK; ^11^ Institute of Biochemistry and Cell Biology, CNR Rome Italy; ^12^ Cambridge Institute for Medical Research University of Cambridge Cambridge UK

**Keywords:** endolysosomes, lysosomal storage diseases, lysosome biogenesis, lysosome exocytosis, lysosome‐related organelles, lysosomes, membrane contact sites, mTOR, TFEB

## Abstract

Since the discovery of lysosomes more than 70 years ago, much has been learned about the functions of these organelles. Lysosomes were regarded as exclusively degradative organelles, but more recent research has shown that they play essential roles in several other cellular functions, such as nutrient sensing, intracellular signalling and metabolism. Methodological advances played a key part in generating our current knowledge about the biology of this multifaceted organelle. In this review, we cover current methods used to analyze lysosome morphology, positioning, motility and function. We highlight the principles behind these methods, the methodological strategies and their advantages and limitations. To extract accurate information and avoid misinterpretations, we discuss the best strategies to identify lysosomes and assess their characteristics and functions. With this review, we aim to stimulate an increase in the quantity and quality of research on lysosomes and further ground‐breaking discoveries on an organelle that continues to surprise and excite cell biologists.

## Graphical abstract

Lysosomes play essential roles beyond their well‐known degradative function, including nutrient sensing, intracellular signalling and metabolism. To better understand these functions, it is essential to employ the best methodological approaches and know their advantages and limitations. Here, we review current methods used to analyze lysosome morphology, positioning, motility and function. The proper use of these methods will allow new discoveries to be made about this fascinating organelle.

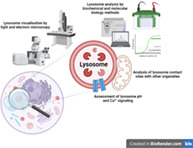

## 1 INTRODUCTION AND HISTORICAL PERSPECTIVE

The discovery of lysosomes dates from 1949, at the dawn of the cellular and molecular ‘revolution’ that ensued in the second half of the 20^th^ century.^1^, ^2^, ^3^ Like many others, the discovery of the lysosome as the cellular organelle responsible for intracellular digestion and recycling of macromolecules was serendipitous. The original goal of Christian de Duve's project was to study glucose‐6‐phosphatase and insulin action in cells. During glucose‐6‐phosphatase purification, his attention turned instead to a control enzyme, namely acid phosphatase, because of its abnormal behaviour upon differential centrifugation.^1^ Elegant experiments then led to the discovery of lytic bodies, described as compartmentalized (structure‐linked latency) activity. Novikoff and de Duve subsequently produced the first electron microscopy (EM) images of lysosomes in 1955 and later demonstrated that they contain degradative enzyme activities by cytochemistry.^4^ The next important conceptual development was the establishment of a physiological link between lysosomes, endocytosis and phagocytosis. Further developments included the concept of receptor‐mediated endocytosis and the discovery of early and late endosomes, as well as phagosomes, as compartments that form prior to the delivery of cargo to pre‐existing lysosomes.^5^, ^6^, ^7^, ^8^, ^9^ With the birth of the membrane traffic field came the discovery of lysosomal enzyme sorting via mannose‐6‐phosphate receptors (MPRs) in the Golgi apparatus, routeing them from their original organelle of synthesis, the endoplasmic reticulum (ER), to lysosomes.^10^ Lysosomes are also the terminal destination for autophagy (a term coined by de Duve in 1963),^11^ the self‐digestion process of cells.^12^ New aspects of lysosome function have been progressively uncovered, such as the formation of membrane contacts with the ER that promote lipid exchange and Ca^2+^ signalling^13^; lysosome:mitochondria contact sites, which are implicated in reciprocal regulation of lysosome and mitochondrial dynamics^14^; and the process of lysosome exocytosis, which, among other functions, plays a crucial role in plasma membrane repair.^15^

The discovery of lysosomes had a significant impact in medicine, as functional defects in lysosomes were linked to a group of genetic diseases, many of which are characterized by neurologic impairment, termed lysosomal storage diseases (LSDs).^16^ The first description of an LSD was that of acid alpha glucosidase deficiency by Hers *et al*., in 1963.^17^

More recently, the discovery of a regulatory mechanism that links the cellular metabolic state with lysosome renewal had a significant impact in the field. Feeding leads to the activation of the metabolic sensor kinase mammalian target of rapamycin (mTOR) at the surface of lysosomes to aid in the recycling of the digested macromolecules. Conversely, cellular starvation leads to inhibition of mTOR and the activation of the master transcription factor EB (TFEB), responsible for the expression of a set of genes leading to the biogenesis of new lysosomes.^18^ These discoveries highlight many aspects of the lysosome life cycle and function that are still poorly understood.

Recent advances in lysosome biology have been comprehensively described in excellent recent reviews.^18^, ^19^ Here, we focus on how lysosomes are recognized before describing state‐of‐the‐art methods to analyze lysosome morphology, positioning, motility and function. We highlight the principles behind these methods, the methodological strategy and their advantages and pitfalls. Importantly, researchers must be aware of what they can extract from the results and how to perform them correctly to yield accurate and reproducible results. Therefore, we also include detailed protocols in Data S1. We hope that this review will catalyze more research of high quality into this fascinating organelle.

## 2 LYSOSOME IDENTITY

Progression along the endocytic pathway from early to late endosomes is characterized by a gradual lowering of pH, removal of recycling proteins and accumulation of intraluminal vesicles that form by inward invagination of the endosomal limiting membrane. Endosomes also have an important role in lysosomal maintenance by delivering newly synthesized lysosomal membrane proteins and enzymes. Many lysosomal enzymes are delivered from the *trans*‐Golgi network (TGN) to endosomes via the cation‐dependent or the cation‐independent MPR, where they dissociate from the receptor in the acidic endosome lumen. MPRs recycle to the Golgi while the lysosomal enzymes are retained within the endosome lumen for subsequent lysosomal delivery. The low pH of late endosomes, their accumulation of internal membranes and their content of newly synthesized ‘lysosomal’ proteins, raises the question of how lysosomes can be distinguished from endosomes. Lysosomes are, however, distinct entities to which soluble cargo and intraluminal vesicles are delivered via full fusion or kiss‐and‐run (transient fusion followed by resealing of the two organelles) between late endosomes and pre‐existing lysosomes.^20^, ^21^ This is a step that is necessary for full degradation of endocytic cargos.

**FIGURE 1:**
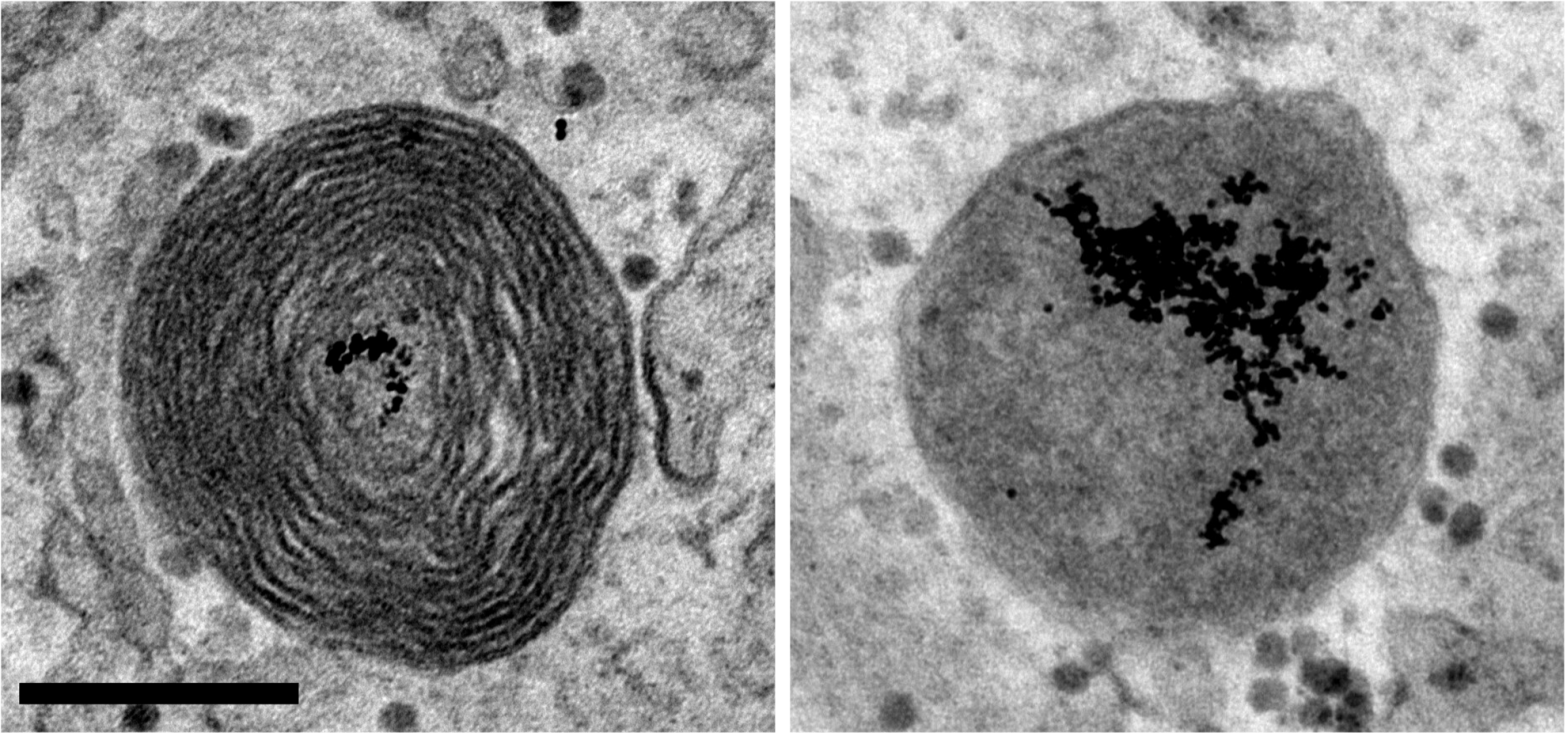
Transmission electron micrographs of electron‐dense lysosomes containing endocytosed gold particles. Primary porcine retinal pigment epithelial cells were incubated with bovine serum albumin (BSA)‐gold for 2 hours, followed by overnight chase to load lysosomes. Gold particles aggregate in the acidic environment of the lysosome after degradation of the BSA. Lysosomes are electron dense and sometimes contain membrane whorls. Scale bar, 200 nm

Lysosomes are heterogeneous in terms of morphology, composition, pH and intracellular distribution.^22^, ^23^ Most spherical lysosomes are in the size range of 200 to 600 nm and are usually distinguished at the ultrastructural level from endosomes by their electron density and the frequent presence of membrane whorls (Figure 1). Tubular lysosomes are also common in cell types such as macrophages and dendritic cells where they play a role in antigen presentation.^24^ Some cell types also contain lysosome‐related organelles with distinctive contents and morphologies (see Section 3).

The number and identity of lysosomal membrane proteins depends on the cell type, the method of isolation and how a lysosomal membrane protein is defined. As an example, more than 700 proteins were found enriched in liver lysosomal membranes, of which more than 200 were known or predicted endolysosomal proteins.^25^ A list of integral membrane proteins predominantly localized to lysosomes can be found elsewhere.^26^, ^27^ The lysosomal‐associated membrane proteins (LAMPs) 1 and 2 are commonly used lysosomal markers, because they constitute about 50% of the lysosomal membrane protein content. They are heavily glycosylated and form a glycocalyx lining the inner surface of the limiting membrane, protecting this membrane from the hostile luminal environment, and also have additional functions (*e.g*., LAMP2 is involved in chaperone‐mediated autophagy^28^). Recent data, however, suggests that in some cell types, not all LAMP‐positive vacuoles contain common acid hydrolases like cathepsin B and D.^23^ Even if the enzymes are present, not all LAMP‐positive lysosomes have the low pH optimal for acid hydrolase activity.^29^, ^30^ Thus, LAMP staining alone may be insufficient to identify degradation‐competent lysosomes.

Degradative enzymes, of which there are over 70, are also used as lysosomal markers, although the relative abundance of different enzymes can vary between cell types. As described above, many lysosomal membrane proteins and enzymes, including LAMPs and cathepsins, respectively, are delivered to lysosomes via the endocytic pathway and so will not be entirely restricted to lysosomes. Indeed, the degree to which a ‘lysosomal marker’ extends along the endocytic pathway can be highly cell type‐dependent. One way around this issue is to compare the distribution of the ‘lysosomal’ marker with that of MPRs, which can be found throughout the endocytic pathway, but not in lysosomes. Additional potential lysosomal markers include subunits of the vacuolar (H^+^)‐ATPase (v‐ATPase), which maintains the acidic lumen of the lysosome, and multiple ion channels (*e.g*., transient receptor potential channel mucolipin 1 [TRPML1]) and transporters (*e.g*., lysosomal membrane integral membrane protein 2 [LIMP2] and Niemann Pick type C1 [NPC1]). Components of the trafficking machinery that regulate the interaction/fusion of lysosomes with other organelles are also used as lysosomal markers. These include small guanosine triphosphatases (GTPases; *e.g*., Rab7 and ADP ribosylation factor‐like 8b [Arl8b]), the homotypic fusion and vacuolar protein sorting (HOPS) complex proteins, some soluble *N*‐ethylmaleimide sensitive factor attachment protein receptors (SNARE) proteins, including vesicle associated membrane protein 7 (VAMP7) and syntaxins 7 and 8, as well as the Ca^2+^ binding protein synaptotagmin VII.

**FIGURE 2:**
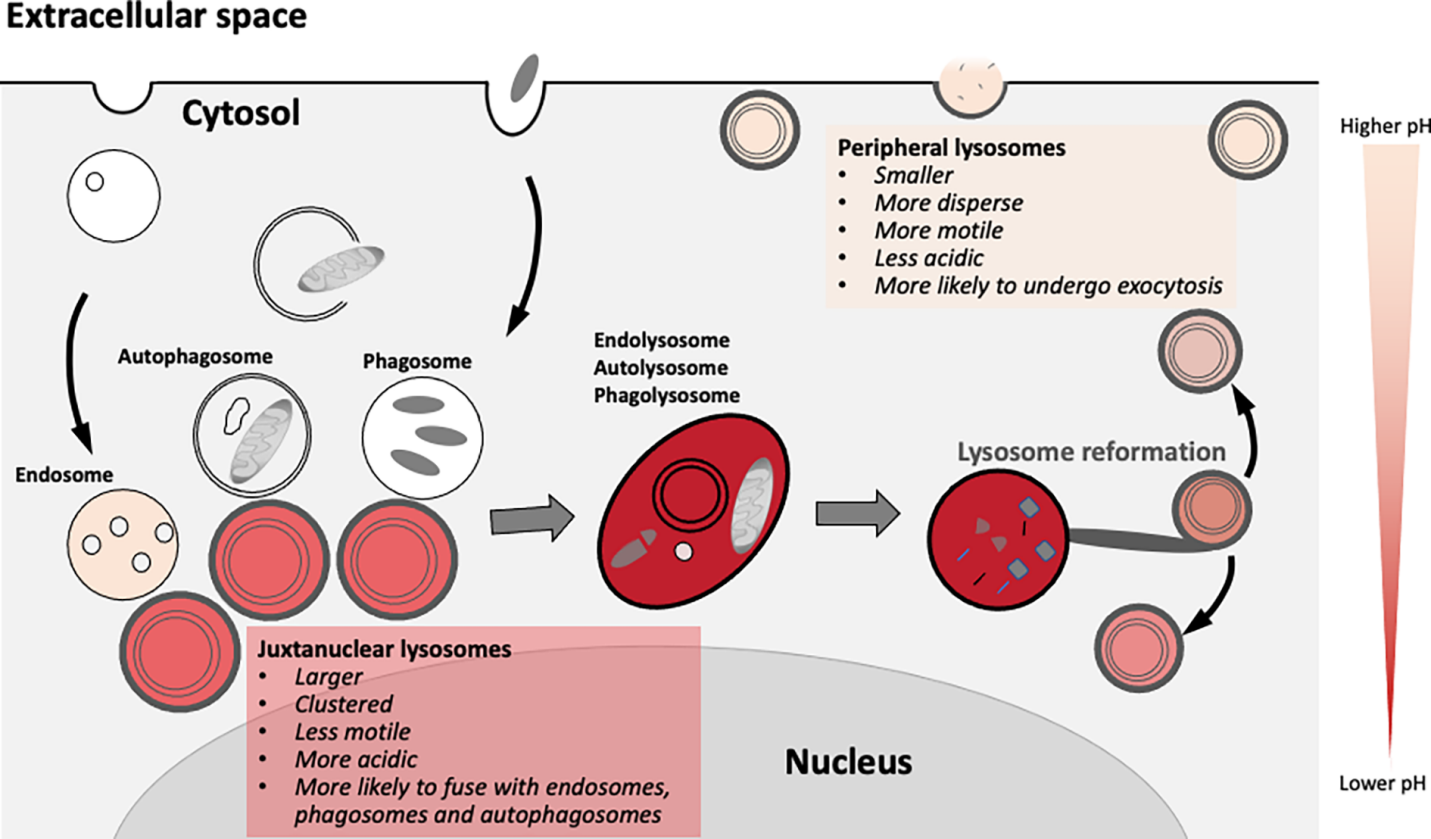
Lysosome heterogeneity and the lysosome cycle. Lysosomes accumulate near the nucleus, where they fuse with endosomes, phagosomes or autophagosomes to generate highly acidic degradative organelles (endolysosomes, phagolysosomes or autolysosomes). A process of lysosome reformation then occurs and can involve tubulation and budding to form less acidic protolysosomes, which may require replenishing with lysosomal enzymes. Lysosomes are also found peripherally, where they tend to be smaller and less acidic, and can be stimulated to fuse with the plasma membrane during lysosome exocytosis

Delivery of cargo for digestion by lysosomal hydrolases requires kiss‐and‐run and/or fusion of lysosomes with late endosomes, autophagosomes or phagosomes to form digestive lysosomal compartments termed endolysosomes, autolysosomes and phagolysosomes, respectively, from which lysosomes can be reformed.^31^, ^32^ For example, autolysosome reformation involves tubulation of the autolysosomal membrane, budding of protolysosomes from the tubule tips and repopulation of the protolysosomes with lysosomal enzymes.^33^, ^34^ Reformed lysosomes are available for subsequent fusion events and thus, lysosomes function in a fusion‐reformation cycle. Organelles at any stage in the cycle may be regarded as lysosomes, but they will have different properties in terms of morphology, luminal pH, hydrolase content and function. Variation in lysosomal pH is also linked to position within the cell, with more acidic lysosomes enriched in the juxtanuclear region and less acidic lysosomes in the periphery (Figure 2).^30^ The v‐ATPase is essential for pumping H^+^ ions into the lysosome and its regulation, as well as counter‐ion transport and H^+^ leakage, together play a role in determining lysosome pH.^30^, ^35^, ^36^

Variation in lysosome age is another potential contributor to lysosomal heterogeneity. In healthy cells, lysosome reformation via the lysosome cycle may be sufficient to maintain cellular degradative capacity but lysosome biogenesis can be activated by nutrient starvation or lysosomal stress (see Section 4.7), with new lysosomes potentially differing from pre‐existing organelles in size, composition and distribution.

## 3 LYSOSOME‐RELATED ORGANELLES

Lysosome‐related organelles (LROs) were originally described as cell‐type specific specialized secretory organelles, mainly found in cells of the haemopoietic lineage, which share characteristics with lysosomes. LROs are formed using machinery that was first identified in studies of genetic disorders such as Chediak‐Higashi syndrome, Griscelli syndrome and Hermansky‐Pudlak syndrome (see exemplary early references [37, 38, 39, 40, 41]). In most cell types, LROs coexist with conventional lysosomes, but at least in cytotoxic T‐lymphocytes, the lytic granules assume the functions of both LRO and lysosome.^42^

While LRO‐specialized contents and their morphology are very diverse, partly reflecting different contents, at least some of the machinery underlying their biogenesis and secretion is shared, including the small GTPase Rab27, which is usually found on the membrane of LROs, while absent from conventional lysosomes.

Conventionally, LROs arise from an endosomal compartment modified by receiving specialized cargo delivered by an adaptor protein complex (AP)‐3‐dependent route. However, this is not the only model; endothelial LROs (*i.e*., Weibel‐Palade bodies) initially form in an AP‐1‐dependent process at the *trans*‐Golgi network (TGN),^43^ where much of their cargo is acquired, but following scission also receive subsequent deliveries of cargo from endosomes in an AP3‐dependent process. AP‐1 is also needed for the formation of a canonical LRO, being essential to forming a subset of endosomal carriers delivering cargo to maturing melanosomes, the LROs present in melanocytes.

The number of identified LROs has grown to include approximately 30 examples, and it has also emerged that lysosomes themselves, as well as endosomes and autophagosomes, are capable of regulated secretion, blurring the distinction between lysosomes and LROs. However, ‘LRO’ is still a generally accepted descriptor, and is thus still of utility—although definitions are debated, providing recent lists and excellent bibliographies (see, *e.g*., Refs. [44, 45]). We therefore append a shortened list of generally accepted LROs with some key features, and links to useful papers (Table 1).

**TABLE 1:** Examples of lysosome‐related organelles (LROs)

Organelle name	Cell type	Organ	Function	Major content(s)	Membrane markers	Regulators of biogenesis	Phenotype when defective	Disease(s)	Refs.
Melanosome	Melanocytes	Skin (and hair)	Pigmentation	Melanin	TYRP‐1, ATP7A, OCA2, MART‐1, DCT	AP‐1, AP‐3, BLOC, Rab32, Rab38	Hypopigmentation, albinism	Hermansky‐Pudlak syndrome, Chediak‐Higashi syndrome, Griscelli syndrome	[46, 47]
Weibel‐Palade body	Endothelial cells	Endothelium	Vascular development and homeostasis, haemostasis.	von Willebrand factor (VWF)	P‐selectin, CD63, Rab27a (mature only)	AP‐1, AP‐3, Rab27, MyRIP	Bleeding and multiple vascular dysfunctions	Von Willebrand's Disease, HPS, Griscelli, etc	[48, 49]
Platelet alpha‐granule	Platelets	Blood	Haemostasis	Many proteins, including VWF, fibrinogen, PF4 and beta‐thromboglobulin	P‐selectin	VPS33B, VPS16, NBEAL2	Bleeding	von Willebrand's disease, Hermansky‐Pudlak syndrome, Griscelli syndrome, arthrogryposis‐renal dysfunction‐cholestasis syndrome, grey platelet syndrome, Wiskott‐Aldrich, Bernard‐Soulier, Glanzmann thromasthenia syndromes.	[49, 50, 51]
Platelet dense granule	Platelets	Blood	Haemostasis	Small molecules; including serotonin, ATP/ADP, Ca	MRP4 nucleotide transporter, CD63, LAMP1, LAMP2	BLOC, AP‐3	Bleeding	Hermansky‐Pudlak syndrome, Chediak‐Higashi syndrome, Griscelli syndrome	[49, 51, 52]
Lamellar body	Alveolar type II epithelial cells	Lung	Breathing	Surfactants (SP‐1, SP‐B, SP‐C, SP‐D)	ABCA3, LAMP3	AP‐3	Collapse of alveoli, lung fibrosis	Hermansky‐Pudlak syndrome	[53]
MHC Class II compartment	Antigen‐presenting cells (dendritic cells, B lymphocytes, macrophages, Langerhans cells)	Immune system	Processing of antigenic peptides for presentation: Adaptive immune response	Antigen‐presenting molecules (MHC Class II, HLA‐DM)	LAMP1	Arl8, Arl14, Arf7, AP‐1, AP‐2.	Immune deficiency	Chediak‐Higashi syndrome	[54, 55]
Eosinophil secretory (specific) granule	Eosinophils	Bone marrow/blood	Inflammatory, allergic and immunomodulatory responses	MBP, PRG2, ECP, EDN, EPX/O, plus cytokines, chemokines and growth factors	CD63, VAMP7 and 8	Poorly understood	Asthma	poorly understood	[56]
Ruffled border/secretory lysosome	Osteoclasts	Bone	Bone remodelling	Cathepsin K	v‐ATPase, OSTM1, Rab7	Rab7 Rab27; v‐ATPase‐alpha3	Petrosis/osteoporosis	Griscelli syndrome	[57, 58]
Azurophilic granule	Neutrophils	Blood	Innate Immunity	Myeloperoxidase, cytokines, chemokine, lysosomal enzymes	CD63	AP‐3	Neutropenia	Hermansky‐Pudlak syndrome	[59]
Acrosome	Sperm	Semen	Fertilization	Acrosin, other hyodrolases	Spaca1 (SAMP32)	Clathrin, CALM, GOPC, ATG7, VPS54/GARP, GM130	Infertility, globozoospermia	Not defined	[60, 61]
Fusiform vesicle	Facet AKA Umbrella cells	Urothelium	Expansion‐contraction cycle of urothelium	Uroplakins	Rab27b	VPS33A/HOPS complex, Rab8, Slac2‐a, Slp2‐a, Vamp8	Loss of FVs, accumulation of multivesicular bodies	Hermansky‐Pudlak syndrome	[62]
Notochord vacuole	Notochord inner cells	Notochord	Formation of intervertebral discs	Liquid providing hydrostatic force	LAMP1	Rab32a	Scoliosis	Hermansky‐Pudlak syndrome	[63]
Cytolytic granule	NK cells, CTLs	Immune system	Elimination of virally‐infected or oncogenic target cells	Perforin, lytic enzymes (eg granzyme)	LAMP1, CD63		Immune deficiency, viral infections	Griscelli syndrome	[64, 65]

## 4 METHODS TO ANALYZE LYSOSOME MORPHOLOGY, POSITIONING, MOTILITY AND FUNCTION

### 4.1 Lysosome visualization by light microscopy

Microscopic visualization is one of the first steps in the analysis of lysosomes. The discovery of new lysosomal proteins along with the development of new antibodies and dyes to selectively label lysosomes have provided an invaluable set of tools for the study of this subcellular compartment.

The visualization of lysosomes by light microscopy, mainly using fluorescence, can be achieved through different approaches: (a) immunolabelling of lysosomal proteins, either membrane‐bound proteins or soluble proteins such as enzymes; (b) use of cargoes internalized by endocytosis; (c) use of freely diffusible dyes that emit fluorescence only in the lysosomal acidic environment or in the presence of active lysosomal hydrolases. Immunolabelling of lysosomes can be performed by using antibodies against lysosomal membrane proteins, such as LAMP1 and LAMP2.^66^ Several antibodies are suitable for immunofluorescence microscopy of fixed and permeabilized cells (a complete list of antibodies referred throughout the review can be found in Table S1), including the mouse monoclonal anti‐human LAMP1 from the hybridoma bank (Iowa State University, H4A3), the rabbit polyclonal anti‐LAMP1 from Abcam (ab24170) and the rat monoclonal anti‐LAMP1 (Santa Cruz biotechnology, sc19992). Anti‐LAMP1 H4A3 is suitable only for use in human cells, whereas the rabbit polyclonal anti‐LAMP1 can be used for human and murine cells, and the rat monoclonal can be used on mouse tissues.^67^ However, LAMP1 is present in both late endosomes and lysosomes. Therefore, to better differentiate late endosomes from lysosomes, it is possible to use markers of late endosomes, such as CD63 (Hydridoma bank, H5C6), which specifically labels the intraluminal vesicles of those organelles, or MPRs, thus allowing a distinction between late endosomes (LAMP1‐positive/CD63‐positive/MPR‐positive) and terminal lysosomes (LAMP1‐positive/CD63‐negative/MPR‐negative).^68^

Labelling of luminal lysosomal hydrolases can also be used in this context, for which different approaches are available. One is immunolabelling with antibodies against cathepsins such as cathepsin B (Abcam, ab214428) or cathepsin D (Abcam, ab75852). These antibodies allow the visualization of lysosomes either by immunofluorescence or immunohistochemistry. Other suitable methodologies rely on the use of fluorescently labelled inhibitors of lysosomal hydrolases such as Bodipy FL‐Pepstatin A (Invitrogen, P12271), a cathepsin D inhibitor that targets it within lysosomes of living and fixed cells, or fluorescently labelled small inhibitors that specifically recognize active lysosomal hydrolases such as the glucocerebrosidase (GBA).^69^ In addition to the labelling of endogenous lysosomal proteins, an alternative technique to visualize lysosomes is the transient or stable expression of tagged lysosomal proteins. One example is C‐terminal GFP‐tagged LAMP1, which is very useful to track late endosomes‐lysosomes in live imaging experiments. Another strategy to visualize lysosomes is using cargoes internalized through endocytosis, either receptor‐mediated or fluid‐phase that are delivered to lysosomes (a complete list of cargoes, dyes and reagents referred throughout the review can be found in Table S2). Dextrans, which are available with different molecular weights and crosslinked to a variety of fluorophores, enter the cells by fluid‐phase endocytosis and reach lysosomes after trafficking through early and late endosomes^22^ (see Section 4.1.1). In addition to dextrans, other cargoes may be used for their ability to localize to lysosomes such as fluorescently‐labelled epidermal growth factor (EGF) and fluorescently‐labelled bovine serum albumin (BSA), available in two forms: Alexa Fluor‐BSA, which emits fluorescence in all the compartments of the endocytic pathway and DQ‐BSA (see Section 4.1.2), which emits fluorescence only within lysosomes. The same concept can be applied using gold‐conjugated BSA for identifying lysosomes by EM (see Section 4.2). One of the main limitations of Alexa Fluor‐conjugated BSA and EGF is that they are susceptible to degradation within lysosomes. This means that a fine tuning of pulse/chase timepoints must be performed in a cell line‐specific manner. An alternative way to visualize this compartment is the use of freely diffusible dyes that enter lysosomes and emit fluorescence in the presence of active cathepsins (*e.g*., Magic Red—see Section 4.12.2) or an acidic environment (*e.g*., LysoTracker—see Section 4.4.1). Noteworthy, any perturbation in pH caused by experimental treatment or certain disease states can affect the signal, so caution must be used in these situations when interpreting the data.

Visualization of lysosomes by fluorescence microscopy is also a fast and reliable way to infer possible pathological alterations in lysosomes by studying the distribution, size and presence of accumulated luminal material. However, fixation and permeabilization conditions may require optimization, especially in the case of co‐labelling of lysosomes and other markers. If the labelling of a marker of interest requires harsher permeabilization (*i.e*., with Triton X‐100), this can interfere with the localization of LAMPs. In this case, a two‐step immunofluorescence labelling is required. For this, cells are fixed and permeabilized with saponin or digitonin, blocked with BSA or species‐matched serum and incubated with anti‐LAMP antibody and subsequently with a fluorescently‐labelled secondary antibody. Cells are then post‐fixed with paraformaldehyde (PFA) and then permeabilized with Triton X‐100, without perturbing LAMP localization. As noted above, it is essential to keep in mind that LAMPs do not label lysosomes specifically, as these molecules are present also in late endosomes, at the plasma membrane and on LAMP carrier vesicles^70^, ^71^ and therefore, other complementary methods must be used.

#### 4.1.1 Dextran assay

Dextran is a hydrophilic polysaccharide with low immunogenicity. It can be conjugated with different dyes to evaluate a variety of *in vitro* (*e.g*., membrane permeability, intercellular communication, endocytosis) and *in vivo* (*e.g*., neuronal tracing, tissue permeability, fluid transport tracing) processes. Thus, depending on the applications, it is available in different molecular weights, with a neutral or anionic net charge. Dextrans are biologically inert because they are composed of a poly‐(α‐d‐1,6‐glucose) linkage that cannot be degraded by lysosomal hydrolases. Dextrans are available linked to different fluorophores such as Alexa Fluor, Oregon Green and Lucifer Yellow, the latter being useful also to monitor water flux in intracellular organelles and lysosomes given its sensitivity to deuterium (D_2_O) concentration.^72^ Fluorescence intensity of lysosomal‐localized Lucifer Yellow‐dextran increases in cells incubated in D_2_O‐containing medium according to the extent of D_2_O influx in lysosomes. Dextrans are stable within lysosomes, and, for this reason, represent a *bona fide* marker of lysosomal terminal compartments. The conditions to allow dextran to be used as a reliable lysosomal marker are cell‐specific and careful optimization of dextran concentration and pulse/chase times must be carried out for each cell type. For instance, in kidney epithelial cells, dextran is administered in complete medium at concentrations ranging from 50 to 500 μg/mL, for 2 hours (pulse).^73^ Chase times are crucial for the complete lysosomal localization of dextran. Indeed, after short chase times (1‐2 hours), dextrans mostly localize in LAMP1‐positive vesicles, although they are also found in early endosomes. However, after longer chase times (16‐24 hours), dextrans are completely absent from early endosomes and only present in LAMP1‐positive structures, mainly representing terminal lysosomes and endolysosomes resulting from late endosome‐lysosome fusion (Figure 2).^20^ The availability of dextrans with molecular masses ranging from 3 to 2000 kDa either neutral or anionic and lysine‐fixable opens many possibilities in addition to lysosome visualization. For instance, 3 or 10 kDa fluorescent dextrans can be used to evaluate lysosomal membrane permeabilization (LMP) (see Section 4.11),^74^ because dextrans in this size range can diffuse out of the lysosome in the presence of small pores in the lysosomal membrane. Therefore, dextran leak needs to be considered when low molecular weight dextrans are used to label lysosomes. To avoid misinterpretation of results coming from assays performed using low molecular weight dextrans, cells need to be immediately visualized by live imaging or alternatively, adequately fixed.

**FIGURE 3:**
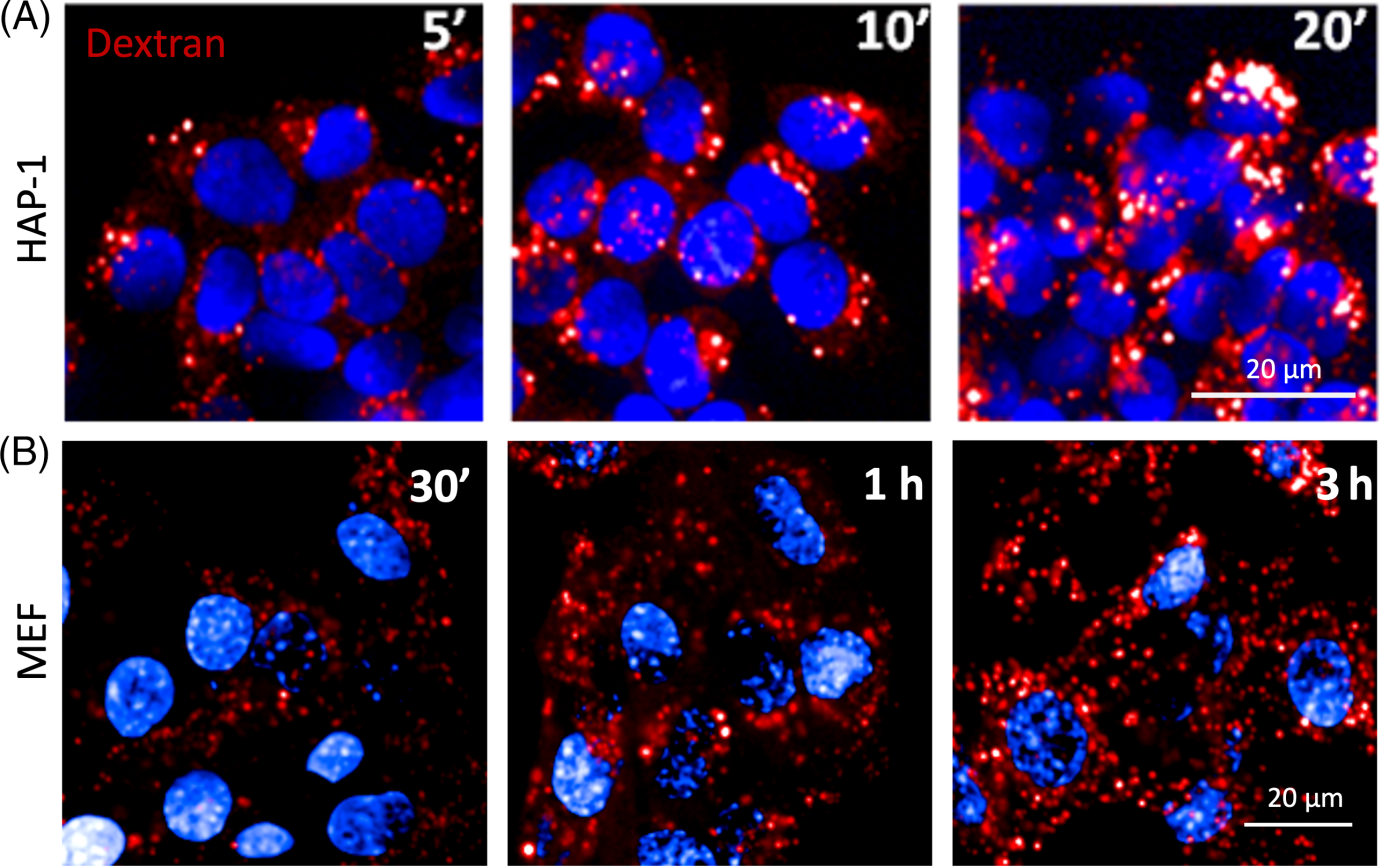
Dextran assay. Representative high content images obtained with Opera (40x water objective) of (A) uptake kinetics (from 5 to 20 minutes) in HAP‐1 cells (cell line derived from chronic myelogenous leukaemia) loaded with Alexa Fluor 568‐dextran (Thermo Fisher, D22912) (in red) and (B) uptake kinetics (from 30 minutes to 3 hours) in mouse embryonic fibroblasts (MEF). Nuclei stained with Hoechst are shown in blue. Scale bar, 20 μm

This assay is a powerful tool to monitor endocytosis^75^ and clearance pathways,^76^ and has been adapted to high content imaging (HCI, Figure 3). In particular, fluorescent dextrans allow the assessment of fluid phase uptake in different cell lines (*e.g*., HAP‐1, Figure 3A; MEFs, Figure 3B) that display different kinetics of uptake and lysosomal delivery. Further details are available in Data S1.

#### 4.1.2 DQ‐BSA assay

**FIGURE 4:**
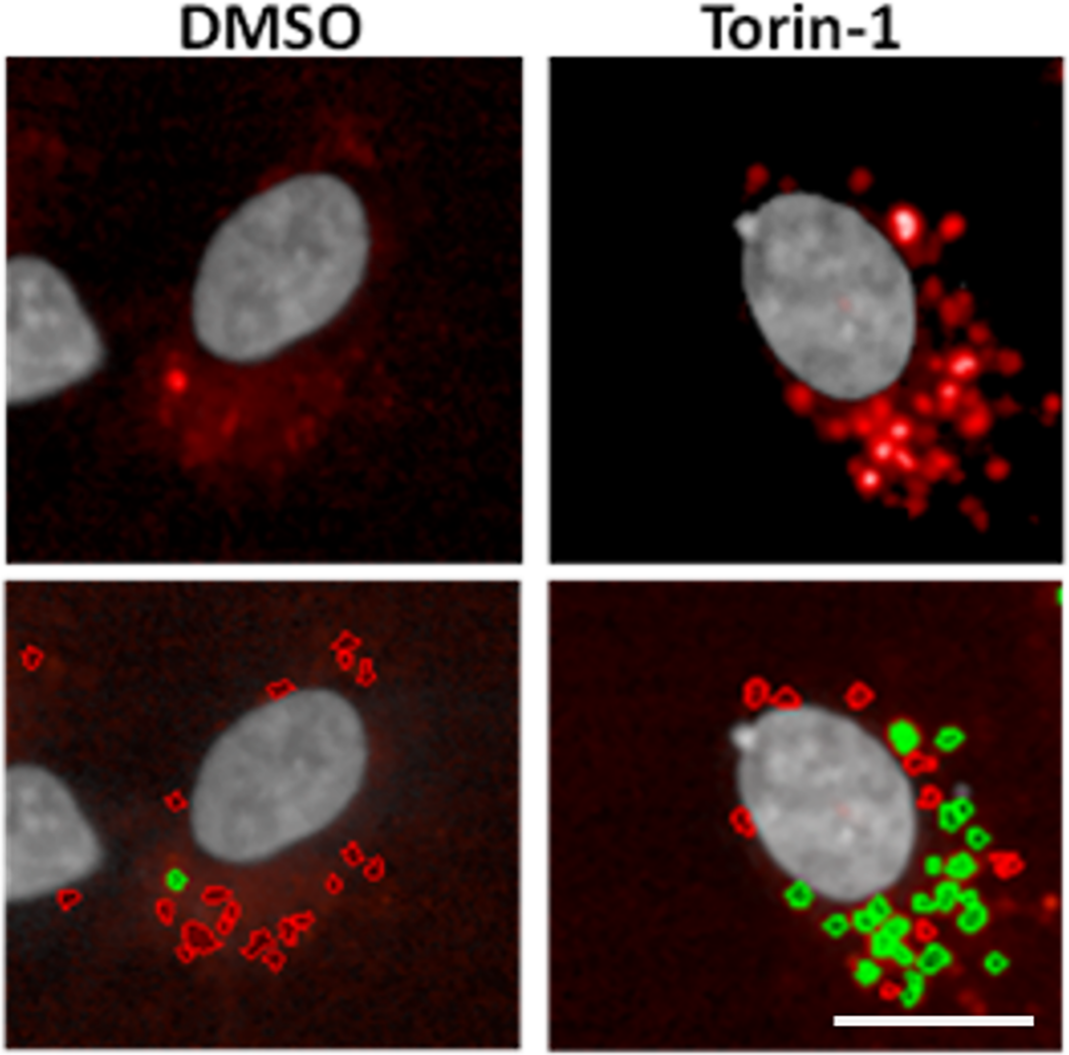
DQ‐BSA assay. Representative high content images of DQ‐BSA puncta in ARPE‐19 human retinal pigment epithelial cells treated with DMSO or Torin‐1 (1 μM for 3 hours) and incubated for 16 hours with 10 μg/mL of DQ‐BSA. Lower row images show the same cells subjected to analysis for the selection of DQ‐BSA puncta (selected spots traced in green; discarded spots with very low fluorescent intensity traced in red). More details in Data S1. Scale bar, 20 μm

DQ‐BSA is a fluorescent probe composed of a BSA derivative conjugated to the self‐quenched red‐BODIPY dye. After DQ‐BSA internalization by fluid‐phase endocytosis, this probe is degraded by proteolysis in lysosomes. This results in the release of bright fluorescent fragments (Figure 4), allowing the visualization of the lysosomal/autophagic degradation capacity.^77^ Some applications of the DQ‐BSA probe are the discovery of cancer therapeutics^78^, ^79^ or the identification of compounds promoting lysosomal function in cellular models of LSDs.^80^ In this assay, cells are incubated with DQ‐BSA (10 μg/mL for 2 hours), fixed and the number and intensity of DQ‐BSA puncta quantified. The advantage of this assay is that it can be used to assess the general degradative capacity of lysosomes and not of a specific lysosomal enzyme activity. Moreover, the red DQ‐BSA probe is pH insensitive and fixable, although the permeabilization step can reduce the signal. In the case that the assay is used to compare the lysosomal degradative capacity of different cell lines or treatments, potential differences in the endocytic capacity or kinetics of the delivery to lysosomes must be controlled by concomitant evaluation of endocytosis and lysosomal delivery of Alexa Fluor‐BSA or dextran, as this could influence the results.

### 4.2 Lysosome visualization by electron microscopy

Despite considerable heterogeneity in size and morphology, lysosomes are recognizable by transmission EM (TEM) as electron‐dense organelles enclosed by a single membrane, typically ranging from 200 to 600 nm in diameter. The formation of electron‐dense multilamellar whorls (probably formed as endosomal intraluminal vesicles start to be degraded) is a useful distinguishing feature of lysosomal ultrastructure^81^ (Figure 1). However, the appearance of lysosomes by TEM can vary, probably representing different stages in the lysosome cycle, with budding/tubular profiles^24^ that may arise during lysosome reformation,^22^ while terminal storage lysosomes may appear as smaller, more spherical, uniformly electron‐dense or amorphous organelles.^82^ Noteworthy, EM was used in the past for aiding in the diagnosis of LSDs.^83^ Because lysosomal morphology can be heterogeneous, with variations between different cell types or conditions (*e.g*., in LSDs) or even within a single population of cells, the following approaches can be taken to facilitate lysosomal recognition, as well as providing additional information (summarized in Table 2).

**TABLE 2:** Electron microscopy methods for analysis of lysosomes

Approach	Description	Pros	Cons	Refs.
Morphology: Conventional TEM ± high pressure freezing (HPF)	Lysosomes appear as relatively electron dense organelles between 200 and 600 nm in diameter often containing multilamellar whorls by conventional TEM	Conventional TEM is relatively straightforward for any lab with EM facilities. HPF‐TEM reduces fixation artefacts for improved ultrastructure.	May sometimes be difficult to distinguish from other organelles, eg, autophagosomes. HPF requires specialist equipment and expertize.	[84, 85]
Internalized probes	Pulse/chase experiments using probes labelled with gold particles or HRP can help identify endocytic organelles. Aggregated gold indicates lysosomal delivery as the protein component that stabilizes the gold is degraded and the gold aggregates in the low pH of the lysosome.	Labels endocytic organelles without the need for immuno‐EM. Pulse/chase experiments with different sized gold particles (or gold and HRP‐labelled probes) allow different endosomal populations and kinetics of lysosomal delivery to be studied.	Can use large amounts of antibody or ligand. Can only be used to trace the endocytic pathway to the lysosome (not Golgi to lysosome traffic).	[84, 86, 87]
Pre‐embedding labelling immuno‐EM	Cells are stained with antibodies specific for lysosomal markers as for IF, but the secondary antibody is conjugated to nanogold rather than fluorescent label. Cells are then prepared for conventional EM, but with an additional nanogold enhancement step.	This is an accessible immuno‐EM technique. Any lab can do the labelling; this immune‐EM approach is therefore available to any lab with access to conventional EM facilities.	Permeabilization can compromise morphology and conditions to retain morphology may not be sufficient to access luminal proteins, so only suitable for labelling the cytosolic domain of lysosomal proteins. Only one protein can be labelled at a time because of variability of the enhanced nanogold size.	[88, 89, 90]
Cryo‐Immuno EM (Tokuyasu method)	Cells fixed in PFA ± low levels of glutaraldehyde and embedded in gelatine are frozen in liquid nitrogen. Ultrathin frozen sections are labelled with antibodies to lysosomal markers at room temperature with up to three antibodies and protein‐A‐gold/gold‐conjugated secondary antibodies of different gold sizes.	Eliminates the possibility of artefacts associated with dehydration and the requirement for permeabilization. Up to three proteins can be labelled at once.	A level of technical skill and specialist equipment is required for ultrathin cryo‐sectioning.	[70, 84, 91]
3D‐EM	Electron tomography (ET): 3D reconstructions are generated from a tilt series through a thicker section.	3D‐ET: High resolution imaging and tomography‐compatible TEM is the only specialist equipment required. Can be coupled with HPF or cryo‐EM for close to native state preservation.	3D‐ET: Relatively small sample depths can be imaged. High volume of data to be processed and stored.	[82, 92, 93]
	Serial block‐face scanning EM (SBF‐SEM): automated serial sectioning with newly exposed surfaces imaged by integrated SEM. Focused‐ion beam scanning EM (FIB‐SEM): Serial milling with a focused beam of high‐energy gallium ions, block‐face imaged by SEM.	SBF‐SEM and FIB‐SEM: Can image through large volumes. Cryo‐FIB‐SEM removes any fixation artefacts.	SBF‐SEM and FIB‐SEM: Expensive specialist equipment is required. Imaging internalized gold‐labelled probes is stretching the resolution. Datasets can be very large.	
	Array tomography/automated tape‐collecting ultramicrotome SEM (ATUM‐SEM): Serial sections are collected on a slide or tape for SEM imaging.	Array tomography/ ATUM‐SEM: Non‐destructive method, sections are kept on slides for future use.	Array tomography/ATUM‐SEM: Requires manual serial sectioning.	
CLEM	Information on lysosomal protein expression and dynamics gained from light microscopy is correlated with EM images.	Dynamic events and localization of fluorescent proteins or probes imaged by light microscopy can be correlated with cellular ultrastructure at EM resolutions.	Technically challenging and time consuming.	[94, 95, 96]

#### 4.2.1 Internalized probes

Coupled with morphological analyses, pulse/chase experiments with fluid phase or receptor‐targeted probes can also provide information on efficiency of lysosomal delivery. Fluid‐phase probes such as BSA or ligands/antibodies to the extracellular domains of proteins targeted for lysosomal degradation can be coupled to electron‐dense gold particles or to horseradish peroxidase (HRP) to aid lysosome visualization. HRP (Sigma, P8250) is relatively resistant to lysosomal degradation and can be readily visualized by the presence of electron‐dense 3,3′‐diaminobenzidine (DAB, Sigma, D12384) reaction product.^21^ HRP can either be internalized by fluid phase or conjugated to endocytosed proteins (*e.g*., EGF)^97^ and chased to the lysosome. Because HRP retains activity following aldehyde fixation, the DAB reaction is usually performed post‐fixation, but can also be used in living cells to inactivate lysosomes preloaded with HRP,^21^ although long‐term culture of HRP‐loaded cells can result in cytotoxicity. As the protein component of gold‐labelled probes is degraded in the lysosome, the gold particles, which mostly appear monodispersed in earlier endocytic organelles, become aggregated in acidic environments, providing a useful indicator of lysosomal fusion and degradative activity.^98^ Thus, coupled with morphological analyses, these probes provide additional information on fusion and degradation, as well as being useful for identification of endocytic organelles.

#### 4.2.2 Immuno‐electron microscopy

To facilitate lysosome identification, lysosomal proteins can be labelled with gold‐conjugated primary antibodies^99^ or unconjugated primary antibodies followed by gold‐conjugated secondary antibodies (details in Data S1) or protein‐A‐gold (available from Cell Microscopy Core Utrecht).^100^ For resin‐embedded samples, labelling can be performed pre‐ or post‐embedding. Alternatively, simultaneous labelling of up to three different proteins is possible on frozen sections by cryo‐immuno EM. Pre‐embedding labelling requires a permeabilization step for antibody detection of lysosomal proteins prior to conventional EM preparation and works well with antibodies targeting the cytoplasmic domain of proteins harboured in the lysosome limiting membrane. However, the mild permeabilization required to retain morphological preservation may compromise antigen accessibility for labelling luminal proteins. Secondary antibodies are labelled with nanogold particles, which are expanded *in situ* to electron‐dense particles visible by EM.^88^ Because of variation in nanogold enhancement rates, only one protein can be reliably labelled at a time. For post‐embedding labelling, specimens are embedded in hydrophilic low temperature resin, such as lowicryl, to preserve antigenicity.^101^ Labelling ultrathin sections overcomes potential issues with intracellular accessibility for antibodies. Alternatively, mild fixation can be coupled with freezing for preservation of hydrated samples in cryo‐immuno EM.^102^ Thawed ultrathin cryosections are labelled, removing the need for permeabilization. Whereas only one protein can be labelled at a time using pre‐embedding labelling, simultaneous labelling of up to three different proteins, distinguished by different sized gold particles, can be achieved by cryo‐immuno EM. This ability to label multiple proteins on a single section, coupled with the absence of potential morphological artefacts from conventional glutaraldehyde fixation or permeabilization makes cryo‐immuno EM a powerful technique. However, specialized equipment is required and cryo‐immuno EM can be technically challenging, although a detailed method has been described by Slot and Geuze.^102^ Immuno‐EM can also be combined with the use of an HRP probe or with the acidotropic probe *N*‐{3‐[(2,4‐dinitrophenyl)amino]propyl}‐*N*‐(3‐aminopropyl)methylamine dihydrochloride (DAMP, Sigma, D1565), which selectively accumulates in acidic organelles and, as well as emitting a fluorescent signal, can also be detected by antibodies targeting dinitrophenol (details in Data S1).^103^

#### 4.2.3 3D‐electron microscopy

While TEM enables imaging at the nanometre scale, information can be lost in two‐dimensional (2D) projections produced from three‐dimensional (3D) samples. Aligned 2D images obtained from thicker sections, tilting the sample in a series of different angles, can be digitally combined to generate 3D reconstructions in a technique known as electron tomography. The increased information provided by 3D tomographic reconstructions is especially useful where the membrane may dip in and out of a 2D projection, for example in characterizing tubular lysosomes^82^ or lysosome connections with other organelles at membrane contact sites (MCS; see Section 4.3).^104^ The 3D reconstructions can also be generated from images obtained through a series of single ultrathin sections, though manual serial sectioning is laborious and sections can be damaged during handling. Automated acquisition of serial images has been developed using serial block‐face scanning EM (SBF‐SEM)^105^ or serial milling with a focused beam of high‐energy gallium ions (focused‐ion beam SEM, FIB‐SEM).^106^ Sequential layers are removed from the block‐face, with each newly exposed surface imaged by integrated SEM.

#### 4.2.4 Correlative light electron microscopy

Lysosomes are highly dynamic organelles and while EM can capture lysosomal positioning, live cell light microscopy of fluorescent probes or ectopically expressed/gene‐edited fluorescent lysosomal proteins is ideally suited for studying lysosome dynamics. By combining information from fluorescence and EM in correlative light EM (CLEM), dynamic cellular events and localization of fluorescent proteins can be visualized in the context of the cellular ultrastructure, at resolutions that can only be achieved by EM. However, this can be a challenging technique. The focal plane needs to be considered for accurate correlation of ultrathin TEM sections with confocal images and light microscopy can be coupled with SBF‐SEM or FIB‐SEM.^94^ The ground‐breaking development of cryo‐EM technology removes issues of poor fluorescence retention and potential membrane shrinkage associated with chemical fixation by imaging frozen hydrated samples.^107^ By correlating cryo FIB‐SEM with light microscopy,^108^ multiple fluorescent proteins can be visualized within close‐to‐native state nanoscale cellular landscapes but highly specialized equipment is required.

### 4.3 Lysosome contact sites

**FIGURE 5:**
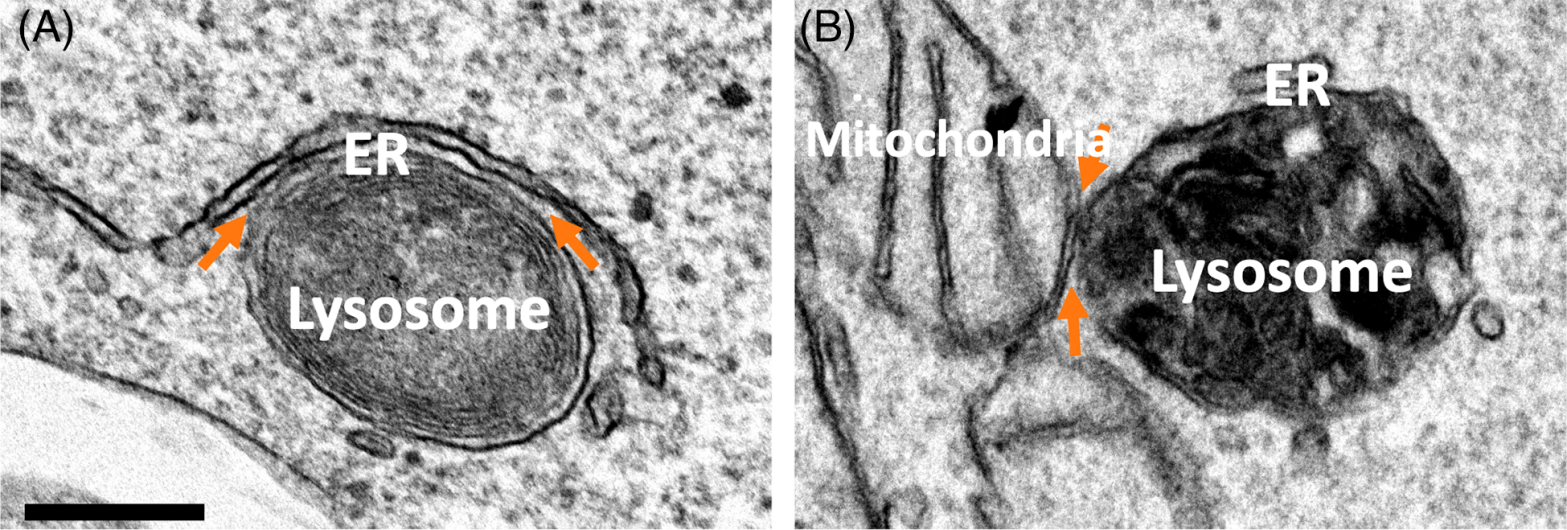
Transmission electron micrographs showing lysosome membrane contact sites (MCS). (A) Lysosome:ER MCS in HeLa cells. (B) Lysosome: mitochondria MCS in HeLa cells incubated with horseradish peroxidase for 6 hours prior to incubation with DAB and preparation for EM. Orange arrows, MCS. Scale bars, 200 nm

There is increasing evidence that lysosome biogenesis and function are strongly influenced by connections with other organelles at MCS. A surge in interest in MCS that form between the ER and late endocytic organelles has led to major advances in our understanding of their regulation and function over the last decade.^109^, ^110^ Roles for MCS in a wide range of essential cellular processes have been described, including organelle dynamics and positioning,^111^, ^112^ Ca^2+^ signalling,^113^, ^114^ endosome tubulation/fission,^115^, ^116^ growth factor signalling^117^, ^118^ and inter‐organelle cholesterol transport.^119^, ^120^ The extent of ER contact with endocytic organelles increases with maturation of the endocytic pathway with over 90% of lysosomes estimated to form an ER contact site,^121^, ^122^ suggesting physiological importance for these interactions. Lysosomes form MCS not just with the ER (Figure 5A) but also with a variety of functionally distinct organelles. Indeed, physical association at MCS between lysosomes and mitochondria has been identified (Figure 5B) and evidence of a role for these MCS in functional crosstalk between the two organelles is starting to emerge. Endosome/lysosome:mitochondria MCS facilitate endocytosed iron delivery to mitochondria for heme biosynthesis in developing erythroid cells^123^ and, in hypoxic cells, MCS are implicated in lysosome‐mediated degradation of damaged mitochondrial proteins.^124^ Lysosome:mitochondria MCS were recently shown to regulate mitochondrial Ca^2+^ dynamics,^125^ as well as being implicated in lysosomal degradation of autophagic and endocytosed cargo.^126^ Furthermore, lysosome:mitochondria MCS influence both lysosome and mitochondrial function through regulation of Rab7 activity and by marking sites of mitochondrial fission, allowing reciprocal regulation of mitochondria and lysosome dynamics at the MCS.^14^

MCS are generally defined as regions where the membranes of neighbouring organelles are 5 to 30 nm apart. As the only means to accurately resolve this distance, EM remains the gold‐standard for MCS analysis, even allowing visualization of the tethers that bridge the interface of the two organelles to stabilize the MCS. However, not all labs have access to EM and while MCS are dynamic, often transient, events, EM is limited to fixed samples. Therefore, several fluorescence microscopy techniques have been developed to study the dynamics of inter‐organelle interactions.

#### 4.3.1 Identification of inter‐organellar associations in fixed samples

Proximity ligation assay (PLA) allows *in situ* detection of protein interactions.^113^ Antibodies raised in different species are targeted to proteins on organelles of interest (*e.g*., LAMP1 for lysosomes). Oligonucleotide‐labelled secondary antibodies (PLA probes, details in Data S1) hybridize only when within close proximity to each other, acting as a primer for amplification. Labelled oligos hybridize to the complementary sequences within the amplicon, to form discrete spots (PLA signals) that can be visualized by confocal microscopy.

#### 4.3.2 Identification of inter‐organellar associations in living cells

Advances in imaging and post‐image analysis of organelle‐targeted fluorescent proteins has transformed our ability to study the dynamics of inter‐organelle associations. Live cell confocal and lattice light sheet spectral imaging has been coupled with imaging informatics to map dynamic MCS between multiple organelles in living cells.^127^ Similarly, structured illumination microscopy (SIM) coupled with single particle tracking has helped uncover the influence of lysosome contact sites on ER distribution.^128^ The advent of and advances in super‐resolution microscopy offer the possibility of being able to resolve the interface at MCS by fluorescence imaging and has been widely applied to studying lysosome MCS,^129^, ^130^ but requires specialized microscopy equipment.

Biochemical approaches have been developed that can be coupled with standard confocal microscopy imaging for studying MCS. These include complementation assay approaches that depend on the generation of a fluorescent signal when two proteins or fragments that are targeted to different organelles are in close proximity. They include fluorescence resonance energy transfer (FRET),^14^ bimolecular fluorescence complementation (BiFC)^131^, ^132^ and dimerization‐dependent fluorescent proteins (ddFP).^133^, ^134^ FRET entails proximity‐dependent energy transfer between donor and acceptor fluorescent proteins, where the emission spectrum of the donor overlaps with the excitation of the acceptor. Thus, when donor and acceptor fluorescent proteins are targeted to two organelles of interest, FRET will occur only when in very close proximity (<10 nm), at MCS. BiFC can similarly be used to visualize protein‐protein interactions in living cells. Split‐fluorescent proteins for BiFC comprise two complementary protein residues targeted to organelles of interest, which only fluoresce when reassembled by protein interaction at MCS. Typically, GFP protein is divided into GFP1‐10 fragment (residues 1‐214) and GFP11 fragment (residues 215‐230), of which GFP 1 to 10 fragment contains three residues of the fluorophore. The fluorophore fluoresces green when GFP1‐10 is complemented with the conserved residue E222 in GFP11.^131^ BiFC has been widely used to study MCS, including lysosome contacts with the ER,^135^ but requires expression of constructs that may themselves affect the MCS stability. An alternative, but similar approach to BiFC, is dimerization‐dependent fluorescent proteins (ddFP) that require binding of two weakly fluorescent monomers, which can be targeted to different organelles, for example lysosomes and ER,^136^ to form a much brighter fluorescent dimer on protein‐protein interaction at MCS.^133^, ^137^ The reversible interaction is unlikely to affect MCS stability but background signal from each weakly fluorescent monomer needs to be considered. Enzymatic complementation assays have also been developed, for example complementation of two luciferase fragments targeting the lysosome and associated organelle of interest (eg, the ER or mitochondria). When the two proteins interact, the subunits come together to form an active enzyme and generate a bright luminescent signal in the presence of the substrate.

#### 4.3.3 Identification of inter‐organellar associations *in vivo*

A splitGFP contact site sensor (SPLICS) has been developed as a one‐step approach to dynamically detect inter‐organelle juxtaposition. This approach has been successfully used to visualize ER:mitochondria MCS in zebrafish neurons,^138^ which were observed at a frequency compatible to that observed in cells in culture. The β11 fragment of the GFP (GFP11) is fused to the cytosolic domain of a lysosome resident protein (*e.g*., LAMP1) and cloned upstream of a self‐cleaving viral 2A peptide. The complementing GFP1‐10 is targeted to the lysosome‐associated organelle of interest (*e.g*., ER or mitochondria) and cloned downstream, to ensure an equimolar amount of the two transgenes. The construct is under the control of a bidirectional UAS promoter, together with a cytosolic DsRed for identification of expressing cells, to allow GAL4‐driven expression of the UAS promoter.

### 4.4 Lysosome pH

A critical aspect of lysosomal function is its acidic pH. Lysosomal acidification is achieved by the activity of the v‐ATPase, a multisubunit protein complex that uses the energy derived from ATP hydrolysis to transport protons across the lysosomal membrane into the lumen of this organelle.^139^

#### 4.4.1 Qualitative assessment of lysosome acidification using LysoTracker

LysoTracker dyes are fluorescent acidotropic probes that label acidic organelles in live cells and are available in a variety of colours and versions such as the Lysotracker Red DND‐99 (Thermo Fisher, L7528), which retains good fluorescence after aldehyde fixation, but not after fixation with other reagents like glyoxal.^140^ They consist of a fluorophore linked to a weak base that is only partially protonated at neutral pH. These probes can traverse biological membranes and label acidic organelles at nanomolar concentrations. However, it is important to note that as a weakly basic amine, LysoTracker may cause lysosome alkalinization. Therefore, in studies with live cells, the analysis should be performed shortly after staining to reduce this effect. Furthermore, these probes exhibit fluorescence that is largely independent of pH, accumulating indiscriminately in acidic intracellular organelles, thereby not accurately reporting on pH. Therefore, LysoTracker probes label lysosomes, as well as late endosomes and for this reason, the fluorescence signal should not be used as a measure of lysosome pH. Importantly, the total fluorescence of these acidotropic dyes is affected by the size, number and nature of the contents of the acidic organelles.^141^ In these cases, the effects of dye accumulation, number, size of organelles per cell and pH are difficult, if not impossible, to deconvolve.^142^

#### 4.4.2 Ratiometric assessment of lysosome pH using exogenous probes

The assessment of the lysosomal luminal pH by ratiometric fluorescence is indicative of relative differences in pH. Moreover, a calibration curve of the signal can be used to convert the fluorescence ratio in absolute pH values. LysoSensor yellow/blue DND‐160 (Thermo Fisher, L7545) exhibits a pH‐dependent increase in fluorescence intensity upon acidification, in contrast with LysoTracker probes.^143^, ^144^ The uptake of LysoSensor probes into living cells occurs within seconds and labels acidic organelles, including lysosomes, at micromolar concentrations. It has both dual‐excitation and dual‐emission spectral peaks that are pH‐dependent, allowing the ratiometric measurement of lysosomal pH using dual‐wavelength fluorescence‐based analysis. In living cells, this fluorescent dye produces yellow fluorescence in acidic environments, whereas it emits blue fluorescence in neutral environments.^143^, ^144^ This property also means that the signal is independent of dye concentration and therefore, the output is not affected by changes in lysosomal size. To ensure that accurate results are obtained, it is necessary to optimize parameters like loading time, concentration and imaging time, as well as generating a pH calibration curve. Like LysoTracker, longer incubation times with these probes can induce an increase in lysosomal pH.^145^ Therefore, images should be taken shortly after staining to reduce this effect.

Ratiometric assessment of lysosomal pH can also be performed using pulse‐chased fluid phase markers, like dextrans labelled with Oregon Green (Invitrogen, D7170), a fluorinated analog of fluorescein that is intrinsically ratiometric or with both a pH‐sensitive fluorophore (*e.g*., fluorescein isothiocyanate [FITC] and pHrodo dyes—Invitrogen, D1820 and Invitrogen, P35368, respectively) and a second, pH‐insensitive fluorophore (*e.g*., Alexa Fluor dyes—Invitrogen, D22913).^146^ The dextran labelling methods are based on the constitutive endocytic activity of cells, which allows dextran to be internalized and, following a sufficient chase time, accumulate in lysosomes (see Section 4.1). Therefore, preliminary experiments using lysine‐fixable dextran chased for various times, followed by immuno‐labelling with known markers of lysosomes should be performed to guarantee specific lysosome labelling. Oregon Green has a pKa of 4.8 and epifluorescence ratiometric imaging of Oregon Green dextran protocols to assess the lysosomal acidification have been described.^36^, ^142^ By measuring fluorescence emission at 535 nm after sequential excitation at 488 nm (a pH‐sensitive wavelength) and 440 nm (a pH‐insensitive wavelength), a ratio that is a reliable index of the luminal pH can be calculated.^30^, ^36^, ^142^ FITC, whose fluorescence decreases with pH, has a pKa of 6.5, allowing a qualitative assessment of the lysosomal pH. In contrast with fluoresceins, pHrodo dyes are weakly fluorescent at neutral pH but increasingly fluorescent as the pH drops. These dyes have a pKa of approximately 6.8. The ratio of the fluorescence emitted by the pH‐sensitive probe to that emitted by the pH‐insensitive probe, which acts as an internal standard for assessment of endocytic uptake and organellar localization, is used as a readout of the pH. Oregon Green dextran conjugates are a more appropriate choice for lysosomal luminal pH measurement because of their pKa and intrinsic ratiometric properties, avoiding differential bleaching of different dyes. However, similar data should be obtained for Oregon Green, fluorescein and pHrodo dyes.

The fluorescence ratio of both pH‐sensitive and pH‐insensitive fluorophores can be measured by flow cytometry. However, in contrast to the use of fluorescence microscopy, discrete regions of interest cannot be selected and little or no spatial or morphological information is obtained. Also, fluorescence from non‐specific extracellular ligand remaining bound to the cell surface may contribute to the overall signal if not accounted for or excluded. Importantly, the use of this method allows imaging for much longer periods than LysoSensor because it does not exhibit the ‘alkalinizing effect’ described for LysoTracker and LysoSensor probes. However, pulse/chase‐loaded fluid‐phase dyes do not always have access to the peripheral pool of lysosomes, even after overnight loading.^30^ This is critical, because the older peripheral lysosomes may have different properties than newly‐formed lysosomes. Furthermore, pulse/chased probes are washed out upon exocytosis.

Finally, to overcome dye loading, leakage, optical imprecisions or photobleaching, efforts have been done do develop new ratiometric probes to quantify lysosome pH (*e.g*., CQ‐Lyso and 2,3‐trimethy‐3‐[2‐(dimethyl‐amino‐4‐yl) vinyl]‐3H‐benzo[e]indole—BiDL^147^, ^148^). Thus, in addition to being ratiometric, the new probes have the ability to utilize a single excitation wavelength, which simplifies data acquisition, minimizes background noise associated with multiple wavelength excitations and reduces autofluorescence interference from cellular organelles and emission in the red or near infrared spectral region. This is because longer wavelength photons have superior tissue penetration and have minimal interference from autofluorescence emitted by cellular components.

#### 4.4.3 Ratiometric assessment of lysosome pH using genetically‐encoded probes

This method is based on the stable or transient exogenous expression of lysosomal proteins tagged with variants of the pH‐sensitive GFP‐based protein and a different fluorescent protein insensitive to pH, like mCherry. Three new genetically encoded pH lysosome biosensors have been recently published: (a) a tandem fusion of pHluorin‐mCherry linked to the luminal domain of LAMP1^149^, ^150^; (b) LAMP1 tagged in the luminal domain with superfolder GFP and at the cytosolic domain with mCherry^129^; and (c) LAMP1 tagged in the luminal domain with monomeric teal fluorescent protein 1 (mTFP1) and at the cytosolic domain with mCherry.^151^ Once the cells are expressing these probes, they can be immediately imaged with most fluorescence microscopes because ratiometric images can be obtained with GFP and mCherry filter sets. This ratiometric fluorescence method allows the robust measurement of lysosomal pH over extended periods of time, from hours to days. This approach also avoids some of the limitations listed for the experimental setups described above, such as dye distribution and exogenous loading of fluid‐phase markers. Importantly, the use of genetically‐encoded ratiometric probes allows the visualization of the peripheral and juxtanuclear lysosome subsets. Transfection efficiency, protein expression levels and distribution patterns can, however, present concerns and problems requiring troubleshooting. Therefore, it is necessary to assess whether overexpression of the genetically‐encoded sensors has any effect on the lysosomal size, positioning or activity.

### 4.5 Calcium signalling to and from the lysosome

Lysosomes contain free Ca^2+^ in the hundred‐micromolar range in their lumen. Furthermore, in contrast to organelles such as the ER, lysosomes and LROs display a membrane potential dependent on K^+^ channels, such as the Ca^2+^‐activated potassium channel (BK) and TMEM175, of around −20 mV (convention states that the positive lumen is considered the same as the extracellular space),^35^, ^152^ which influences the direction of ion movements across the organelle membrane and would promote cation efflux. The role of lysosomes as a Ca^2+^ storage organelle allows them to buffer and release Ca^2+^, and thus are now recognized as playing an important role in Ca^2+^ signalling and homeostasis. Because Ca^2+^ signals are highly localized due to cellular buffering mechanisms, release from small mobile or positioned organelles can deliver Ca^2+^ to a specific cellular target. Lysosomal Ca^2+^ is important for membrane fusion events and trafficking, possibly luminal Ca^2+^ for enzyme activity, and Ca^2+^ release for both distinct and more general roles in Ca^2+^ signalling and thus needs to be measured and manipulated.^153^

Ca^2+^ is taken up into the endolysosomal system from the extracellular milieu by endocytosis.^154^ In addition, there is evidence in many cell types that Ca^2+^ filling/storage is pH‐dependent. However, membrane contact sites between the ER and lysosomes have also been proposed as sites of Ca^2+^ transfer between the two organelles, and this may be bidirectional.^151^, ^152^, ^153^, ^154^ Ca^2+^ release from lysosomes may trigger a much larger Ca^2+^‐induced Ca^2+^ release (CICR) from the ER, which will greatly overestimate their Ca^2+^ content. Hence, determination of lysosomal Ca^2+^ content should be done under conditions of ER Ca^2+^ depletion (in Ca^2+^‐free media to avoid capacitative Ca^2+^ entry) or block of the major ER Ca^2+^ release channels inositol 1,4,5‐trisphosphate receptors (IP_3_Rs) and the ryanodine receptors (RyRs).^155^

A number of Ca^2+^ permeable channels have been demonstrated to be present in lysosomal membranes, which can mediate Ca^2+^ release from lysosomes in response to various stimuli, including metabolic factors. These include two‐pore channel subtype 2 (TPC2), P2X4 and the Trp channels, TRPML1, transient receptor potential melastatin‐related 2 (TRPM2) and transient receptor potential ankyrin 1 receptor (TRPA1).^156^ These channels are not Ca^2+^ selective and pass other cations such as Na^+^ and H^+^.^157^ Ca^2+^ release from lysosomes can be directly measured by expressing channel‐genetically encoded Ca^2+^ indicator (GECI) chimeras containing a lysosomal targeting sequence. This has been done for the luminescent Ca^2+^ reporter protein aequorin, which is less subject to pH influence.^158^ The GECI should be of sufficiently low affinity so that it does not detect general global Ca^2+^ but reports Ca^2+^ fluxes specifically from lysosomes. Controls should be performed comparing Ca^2+^ signals from other sources (*e.g*., the ER). Surprisingly, using this approach, it has been found that Ca^2+^ fluxes are highly compartmentalized, with flux through one class of channel not detected by another channel type bearing the probe.^159^

Luminal measurement of lysosomal free Ca^2+^ can be performed indirectly by emptying the organelle of Ca^2+^ with lysosomotropic agents such as glycyl‐l‐phenylalanine 2‐naphthylamide (GPN, Sigma, G9512; see Table 3), although it may have other mechanisms^160^ or dissipating the proton gradient as described above, and measuring cytoplasmic changes with cytoplasmic Ca^2+^ reporters. Because, as mentioned above, Ca^2+^ release from lysosomes may trigger larger releases from the ER, ER Ca^2+^ release needs to be blocked, as well as Ca^2+^ entry across the plasma membrane. A caveat is that these strategies, at least in the long term, may also lead to the Ca^2+^ depletion of lysosomal stores. Direct methods are more problematic because the acidic environment may quench fluorescent probes and protons may compete with Ca^2+^ for binding, making the unravelling of dynamic luminal Ca^2+^ changes in particular a challenge. Several strategies have been employed to try and circumvent these problems (see Table 4 for a compilation of probes to measure luminal and juxta‐lysosomal Ca^2+^). Dye‐esters do not appear to load into lysosomes sufficiently perhaps surprisingly because of lack of luminal esterases.^153^ However, lysosomes, in contrast to other organelles, are linked to the outside of the cell through the endocytic pathway, and this allows loading from the outside. Therefore, probes can be targeted specifically to lysosomes by either adding membrane‐impermeant dextran‐linked fluorescent Ca^2+^ probes to the extracellular medium and allowing them to be endocytosed and trafficked to the terminal lysosome compartment.^153^, ^173^, ^175^ Here, the same caveats apply as for lysosome pH measurement using dextran‐coupled probes. This is done in concert with a second dextran‐linked probe, which should be Ca^2+^‐insensitive. This ratiometric approach allows for a quantification of free luminal Ca^2+^. Because the acidic pH of the lysosomal lumen decreases the apparent affinity of Ca^2+^ probes, luminal pH must be simultaneously measured and corrected for. For resting lysosomal luminal Ca^2+^ measurements, the acidic pH can be advantageous, as it decreases the *in situ* affinity of probes for Ca^2+^, because low affinity probes are required because the resting levels of lysosomal luminal Ca^2+^ are around 500 μM, which would normally saturate higher affinity indicators. Another recent development is the use of DNA‐based lysosomal fluorescent Ca^2+^ probes such as CalipHluor, which is also pH correctable.^176^, ^177^ These have not been widely used beyond the group that reported them and require uptake into cells, which may be non‐trivial. Other approaches include the use of GECIs (see above) and organelle‐targeted FRET probes, which have been employed for the less acidic endosomes.^178^, ^179^ Finally, the luminal Ca^2+^ environment and the perilysosomal space can also be manipulated to dampen or remove lysosomal Ca^2+^ in a more selective way than the pharmacological approaches above. Dextran‐linked 1,2‐bis(*o*‐aminophenoxy)ethane‐*N*,*N*,*N*′,*N*′‐tetraacetic acid (BAPTA) based probes such as Cal520‐dextran (AAT Bioquest, 20 601) can traffic to the lysosomal lumen, similar to the Ca^2+^ probes mentioned above, to buffer luminal Ca^2+^. Furthermore, a rapalog system has been used to target a Ca^2+^ chelating protein like calbindin, specifically to the lysosomal surface, to chelate Ca^2+^ signals originating from the lysosome.^177^

**TABLE 3:** Lysosomal calcium modulating agents that selectively release endolysosomal calcium and can be paired with cytosolic calcium indicators for an indirect assessment of lysosomal calcium

Drug	Mechanism of action	Practical considerations	Refs.
Glycyl‐l‐phenylalanine‐naphthylamide (GPN)	Peptidase‐dependent lysosomal permeabilization	Appropriate controls are required to check for the pH‐dependent (cathepsin C independent) effects of GPN	[160, 161, 162]
Bafilomycin A_1_/Concanamycin A	v‐ATPase inhibitor	Slow acting as effect depends on H^+^ and Ca^2+^ leak from lysosomes. Effects can be lacking in cells with a minimal lysosomal Ca^2+^ ‘leak’	[153]
Nigericin	K^+^/H^+^ exchanger (Ionophore)	Indirect effect on lysosomal Ca^2+^. Poor selectivity to lysosomes	[153]
Monensin	Na^+^/H^+^ exchanger (Ionophore)		
NAADP	TPC‐mediated Ca^2+^ release	NAADP is cell impermeant. Usually microinjected. Esterified form, NAADP‐AM, not easily accessible commercially and sometimes unstable on storage	[152, 163, 164]
ML‐SA1	TRPML1‐mediated Ca^2+^ release	ML‐SA1 can evoke complex global Ca^2+^ signals in some cells, therefore effective controls are required to elucidate lysosomal contributions	[165, 166, 167]
Sphingosine	TPC1‐dependent lysosomal Ca^2+^ release	Caged sphingosine utilized but not commercially available.	[168]
TPC2A1(N)	TPC2‐dependent lysosomal Ca^2+^ release	Direct activator of TPC2, mimics NAADP	[157]
TPC2A1(P)	TPC2‐dependent lysosomal cation fluxes	Direct activator of TPC2, mimics PI(3,5)P_2_ promotes sodium fluxes and smaller Ca^2+^ fluxes	[157]

**TABLE 4:** Ca^2+^ indicators for measuring juxta‐ and intra‐lysosomal calcium

Ca^2+^ indicator	Localization	*K*_*d*_	Refs.
LAMP1‐YCaM	Juxta‐lysosomal	250 nM^a^	[169, 170]
GCaMP3‐TRPML1	Juxta‐lysosomal	660 nM^a^	[171, 172]
LAMP1‐GGECO1.2	Juxta‐lysosomal	1.2 μM^a^	[113]
TPC2‐GGECO1.2	Juxta‐lysosomal	1 μM	[159]
Fura‐2 dextran (fura‐dextran dye)	Intra‐lysosomal	200 μM	[173]
Cathepsin D‐Aequorin	Intra‐lysosomal	260 μM	[158, 174]
Oregon green BAPTA‐1 dextran	Intra‐lysosomal	500 μM	[173]
Rhod dextran (low affinity)	Intra‐lysosomal	690 μM	[175]
CalipHluor	Intra‐lysosomal	*K*_*d*_ varies with pH	[176]

^a^ Affinity for Ca^2+^ not measured *in situ*.

### 4.6 Lysosome biogenesis

Lysosome biogenesis is the process by which new lysosomes are generated and is essential to guarantee that enough lysosomes are available to support cellular metabolism, in particular catabolic pathways such as autophagy, while generating new building blocks for anabolism. Intracellular trafficking of lysosomal membrane proteins and soluble hydrolases is fundamental for the generation of new functional lysosomes.^180^, ^181^ While basal lysosome biogenesis occurs at a slower rate in steady‐state conditions, specific environmental (*i.e*., nutrient starvation) or genetic (*i.e*., LSDs) cues may boost the induction of this cellular process.^182^ Indeed, when nutrients are scarce, lysosome biogenesis is activated in order to support degradation of autophagic cargoes and hence promote cell survival. In pathological conditions, such as LSDs, lysosome biogenesis assumes greater importance because dysfunctional and storage‐filled lysosomes accumulate and thus, there is a greater need for new lysosomes.^183^

Regulation of lysosome biogenesis is operated at both transcriptional and translational levels. The existence of transcriptional regulation of lysosome biogenesis was demonstrated by the identification of TFEB as a master regulator of several genes encoding lysosomal membrane proteins and hydrolases.^184^ TFEB target genes share common features in their promoters, in particular the presence of one or multiple consensus motif(s) for TFEB binding, the so‐called coordinated lysosomal expression and regulation (CLEAR) motif.^184^, ^185^ In line with its role as transcriptional activator of many lysosomal genes, TFEB overexpression induces lysosome biogenesis and increases cellular ability to degrade macromolecules.^182^ In addition to TFEB, other transcription factors such as ZKSCAN3 have been found to be associated with the regulation, repression in this case, of lysosomal‐autophagy genes during nutrient deprivation.^186^ Furthermore, nutrient mediated activation of the AMP‐activated protein kinase (AMPK) leads to the transcriptional upregulation of the co‐activator‐associated arginine methyltransferase 1 (CARM1), which acts as co‐activator on lysosomal‐autophagy genes.^29^ Increased number of lysosomes is one of the main indicators of lysosome biogenesis. This can be assessed by (a) Western blot (WB) or immunofluorescence using anti‐LAMP1 antibodies (Hybridoma bank, H4A3 and Abcam, ab24170) or (b) measuring lysosome compartment size by analysing LysoTracker fluorescence intensity (see Section 4.4.1). Furthermore, a complementary analysis of lysosomal activity should be performed. In the case of the biogenesis of new and functional lysosomes, the increase in lysosome number and volume should be accompanied by an increase in lysosomal activity. For this reason, analysis of cargo degradation, for example using DQ‐BSA (see Section 4.1.2) is required. Given the pivotal role that TFEB has in the regulation of lysosome biogenesis, analysis of TFEB activity may be indicative that the machinery responsible for the transcription of lysosomal genes is active (see Section 4.7). Furthermore, quantitative PCR analysis of lysosomal genes harbouring CLEAR sites in their promoter^185^ (Table 5) will help determine whether the lysosome biogenesis pathway is active.

**TABLE 5:** List of TFEB target genes involved in lysosome biogenesis and autophagy whose expression can be monitored by quantitative PCR

Gene name	Protein name
*GPNMB*	Transmembrane glycoprotein NMB
*RRAGD*	Ras‐related GTP‐binding protein D
*RRAGC*	Ras‐related GTP‐binding protein C
*FLCN*	Folliculin
*FNIP1*	Folliculin‐interacting protein 1
*LAMP1*	Lysosome‐associated membrane glycoprotein 1
*SQSTM1*	Sequestosome‐1
*UVRAG*	UV radiation resistance‐associated gene protein
*WIPI‐1*	WD repeat domain phosphoinositide‐interacting protein 1
*PPARGC1A*	Peroxisome proliferator‐activated receptor gamma coactivator 1‐alpha
*ATP6V0D1*	V‐type proton ATPase subunit d 1
*ATP6V0D2*	V‐type proton ATPase subunit d 2
*ATP6V1H*	V‐type proton ATPase subunit H
*CTSD*	Cathepsin D
*CTSF*	Cathepsin F
*MCOLN1*	Mucolipin‐1

**FIGURE 6:**
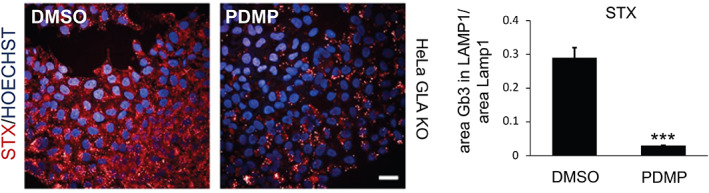
Globotriaosylceramide (Gb3) accumulation in HeLa cells knockout (KO) for α‐galactosidase, treated with 1‐phenyl‐2‐decanoylamino‐3‐morpholino‐1‐propanol (PDMP). Gb3 lipid accumulation in GLA KO Hela cells treated with PDMP (glucosylceramide synthase inhibitor) or DMSO, as a control. This read‐out has been used to develop a Gb3 lipid accumulation assay. PDMP was used as a positive control for Shiga toxin (STX). ***P ≤ .0001, as determined by Student's *t*‐test. Scale bar, 20 μm

In addition to the evaluation of lysosome number as a proxy of lysosome biogenesis, it was recently demonstrated that cell models of LSDs represent valuable tools to analyze lysosomes that have been formed through stimulus‐induced lysosome biogenesis. An assay has been developed in a cell model of Fabry disease, an LSD caused by the loss of function of α‐galactosidase gene (*GLA*), generated by CRISPR/Cas9. As expected, *GLA* knockout (KO) cells display lysosomal globotriaosylceramide (Gb3) storage in almost all LAMP1‐positive vesicles, visualized using fluorescently‐labelled subunit B of Shiga toxin, which specifically binds Gb3. *GLA* KO cell starvation triggers lysosome biogenesis, which does not induce clearance of Gb3 storage from older lysosomes but leads to a marked increase in small LAMP1‐positive vesicles free of Gb3 storage. The appearance of storage‐free lysosomes is indicative of lysosome biogenesis, because in cells in which this process is blocked, there are no storage‐free lysosomes.^187^ These assays are suitable for high throughput screening (Figure 6, further details available in Data S1).

### 4.7 TFEB and mTORC1 activity

Because of the essential role of lysosomes in metabolic signalling, the number and composition of these organelles is subject to fine transcriptional modulation in response to nutrient availability and other environmental cues. The mTOR complex 1 (mTORC1)‐TFEB signalling axis is a major hub that allows the cell to adjust lysosome number, activity and function, based on the cellular nutritional status.

#### 4.7.1 Assessing TFEB and mTORC1 activity *in vitro*

TFEB activity is strictly regulated through post‐translational modifications and spatial organization.^188^ Under nutrient‐rich conditions, TFEB is largely cytosolic and inactive. Conversely, conditions such as nutrient deprivation or lysosomal dysfunction, induce TFEB nuclear translocation and transcriptional induction of its target genes. Thus, monitoring TFEB subcellular localization represents a simple readout to evaluate its activation status.

TFEB localization can be monitored by immunofluorescence analysis using TFEB antibodies, from Cell Signaling (4240) for human cells or from Bethyl Laboratories (A303‐673A), recommended for mouse cells. The active (nuclear) TFEB is dephosphorylated in several serine residues and hence TFEB lower molecular weight can be confirmed by WB using the same antibodies mentioned above. Alternatively, the phosphorylation status of specific serine residues can be monitored by WB using phosphoantibodies, such as phospho‐TFEB (Ser211) from Cell Signaling (37681) or phospho‐TFEB (Ser142) from Millipore (ABE1971). However, because of the low abundance of TFEB in most cell lines, the use of these antibodies usually requires constitutive expression of exogenous TFEB. Furthermore, when assessing TFEB nuclear translocation, cell culture conditions should be carefully controlled because alterations in several environmental conditions (*e.g*., nutrient fluctuation) may alter TFEB activity.

Once in the nucleus, TFEB promotes the expression of several target genes associated with diverse biological functions, including lysosome biogenesis and autophagy,^182^, ^184^, ^185^ whose levels can be monitored by quantitative PCR (Table 5 and Section 4.6). Additionally, TFEB regulates the expression of genes important for activation of mTORC1 signalling, such as the Rag C/D GTPases.^189^ Because the expression levels of RagD are generally low in most cell lines/tissues and dramatically increase upon TFEB activation, RagD represents a very sensitive TFEB target and an optimal readout of its activation state. In addition to RagD, one of the most‐responsive TFEB targets is the gene encoding the glycoprotein non‐metastatic melanoma protein B (GPNMB).

TFEB phosphorylation is mainly controlled by the mTORC1 kinase.^190^, ^191^, ^192^ Thus, TFEB subcellular localization and phosphorylation status may also be used to monitor mTORC1 activity, as in most cases, TFEB dephosphorylation and nuclear localization correlate with inhibition of mTORC1. However, because mTORC1 has been shown to possess substrate‐specific activity,^193^ determining the phosphorylation of multiple mTORC1 substrates in addition to TFEB, is needed to fully assess mTORC1 activation status. These substrates include S6K (probed with phospho‐p70 S6K Thr389 antibody from Cell Signaling, 9206) and 4E‐BP1 (probed with phospho‐4E‐BP1 S65 antibody from Cell Signaling, 9456). In addition, because mTORC1 activation occurs upon its recruitment from the cytosol to the lysosomal membrane,^194^, ^195^ determining mTOR subcellular localization is also an useful readout of mTORC1 activity. This can be achieved by using the anti‐mTOR monoclonal antibody 7C10 from Cell Signaling (2983), which is suitable for use in both human and mouse cells.

#### 4.7.2 Assessing TFEB and mTORC1 activity *in vivo*

Monitoring TFEB activity *in vivo* poses some challenges. Total levels of TFEB can be assessed by WB, using the antibodies referred in Section 4.7.1. However, following TFEB molecular weight shift to monitor TFEB phosphorylation status is more difficult on tissue lysates than in cell lysates. Alternatively, nuclear/cytosolic fractionation can be a valuable method to evaluate the amount of nuclear TFEB and hence its activation status in tissues.^193^ Because of the low abundance of this protein, immunohistochemistry (IHC) techniques to assess TFEB localization in tissues are challenging, even though TFEB IHC (using the antibody from Bethyl Laboratories, A303‐673A) works well in pathological conditions characterized by high TFEB levels. On the other hand, mTORC1 signalling can be monitored by IHC using an antibody recognizing the phosphorylated form of S6 (phospho‐S6 Ser240/244 antibody from Cell Signaling, 5364), an S6K substrate and thus an indirect substrate of mTORC1. In addition, assessing the phosphorylation levels of the mTORC1 substrates S6K and 4E‐BP1 by WB on tissue extracts also allows following mTORC1 activation status *in vivo*. As mentioned above, decreased mTORC1 activity usually correlates with reduced TFEB phosphorylation and, for this reason, it can be generally used as an indicator of TFEB activity. Paradoxically, however, high mTORC1 activity and increased TFEB nuclear translocation are often observed in pathological states.^193^, ^196^ Therefore, analysing TFEB and other mTORC1 substrates is necessary to have a comprehensive characterization of this pathway. Finally, analysis of TFEB target gene expression represents an important method to assess TFEB activity *in vivo* (Table 5). While the regulation of several target genes is conserved between cells and tissues, the regulation of different TFEB targets is tissue‐specific.^188^ Hence, monitoring these specific targets can be particularly important to understand the relevance of TFEB activation in the tissue/organ of interest.

It is important to consider that TFEB is expressed at very low levels in many cell lines. Therefore, a valuable tool to overcome technical issues because of the low protein abundance is the generation of cell lines stably expressing tagged versions of TFEB.^197^, ^198^ In particular, the use of TFEB‐GFP overexpressing cells enables an immediate visualization of TFEB subcellular localization by directly monitoring GFP fluorescence and is an useful method to perform live‐cell imaging analysis of the nucleo‐cytoplasmic shuttling.^197^ In addition, the use of stable cell lines constitutively expressing exogenous TFEB is valuable to assess its phosphorylation using specific phosphoantibodies described above (see Section 4.7.1). The low abundance of TFEB and the lack of good antibodies to detect this protein in mouse cells and tissues makes the analysis of TFEB activity even more challenging in murine samples. For this reason, monitoring the activity of transcription factor E3 (TFE3), another member of the MiT/TFE family of transcription factors, which shares similar activation mechanisms with TFEB,^199^ can be useful to evaluate MiT/TFE activity. TFE3 protein abundance and subcellular localization can be assessed by WB or IHC, respectively, using an anti‐TFE3 antibody from Sigma (HPA023881).

### 4.8 Lysosome positioning

Although lysosomes are broadly distributed within cells, they can be spatially differentiated based on their positioning. Lysosomes can concentrate in the juxtanuclear region, surrounding the microtubule‐organizing centre (MTOC) or localize more peripherally and close to the plasma membrane. Juxtanuclear lysosomes are more acidic, immotile and prone to fuse with endosomes or autophagosomes, while peripheral lysosomes are more motile and can undergo exocytosis^200^ (Figure 2).

The positioning of lysosomes, labelled as described (see Section 4.1), can be evaluated by visual inspection of lysosome clustering, scoring the lysosomes as clustered or not in images from different fields chosen in an unbiased manner or in a blinded fashion.^201^ For statistical significance, more than 300 cells for each condition from at least three independent experiments should be analyzed.^201^ This is a fast method but depends on the choice of cells that show lysosome clustering.

Different strategies have been used to assess lysosome positioning in a more unbiased way. Automated measurement of lysosome clustering can be done with the Clark aggregation index (CAI)^202^ and the measure of the distance of each lysosome (object) from the nucleus, border or centroid.^203^ The CAI is defined as (sum[*D*(*x*))/*N*(*x*)] * sqrt[*N*(*x*)/*A*]), where *D*(*x*) represents the spatial distance to the next neighbour of pixels belonging to the lysosome region; *N*(*x*) the number of pixels belonging to the lysosome region; and *A* the number of pixels of the cell object. This requires lysosome automatic object segmentation (*e.g*., using free spot detector plugin ICY software).^204^ Automatic segmentation guarantees an unbiased measurement of clustering but is more time‐consuming. Another strategy is to calculate the fraction of total lysosomes (intensity) per subcellular region of interest. The subcellular regions can span the whole cell by degrading inwardly from the cell boundary, creating multiple shells of the desired thickness (shrinking cell outlines) using volocity software (https://quorumtechnologies.com/index.php/component/content/category/31-volocity-software).^30^ Alternatively, the radial signal intensity profile around the centre of the nucleus and in concentric annular regions of interest can be obtained semi‐automatically using ImageJ erosion of binary masks^205^ or automatically using an open‐source ImageJ/FIJI macro (https://github.com/PTschaikner/RadialIntensityProfile).^206^ The analysis of lysosome positioning can also be done by assessing the fraction of signal in the peri‐Golgi area, defined by a Golgi marker such as giantin, and calculating the percentage of total lysosome marker fluorescence intensity in this region.^207^

**FIGURE 7:**
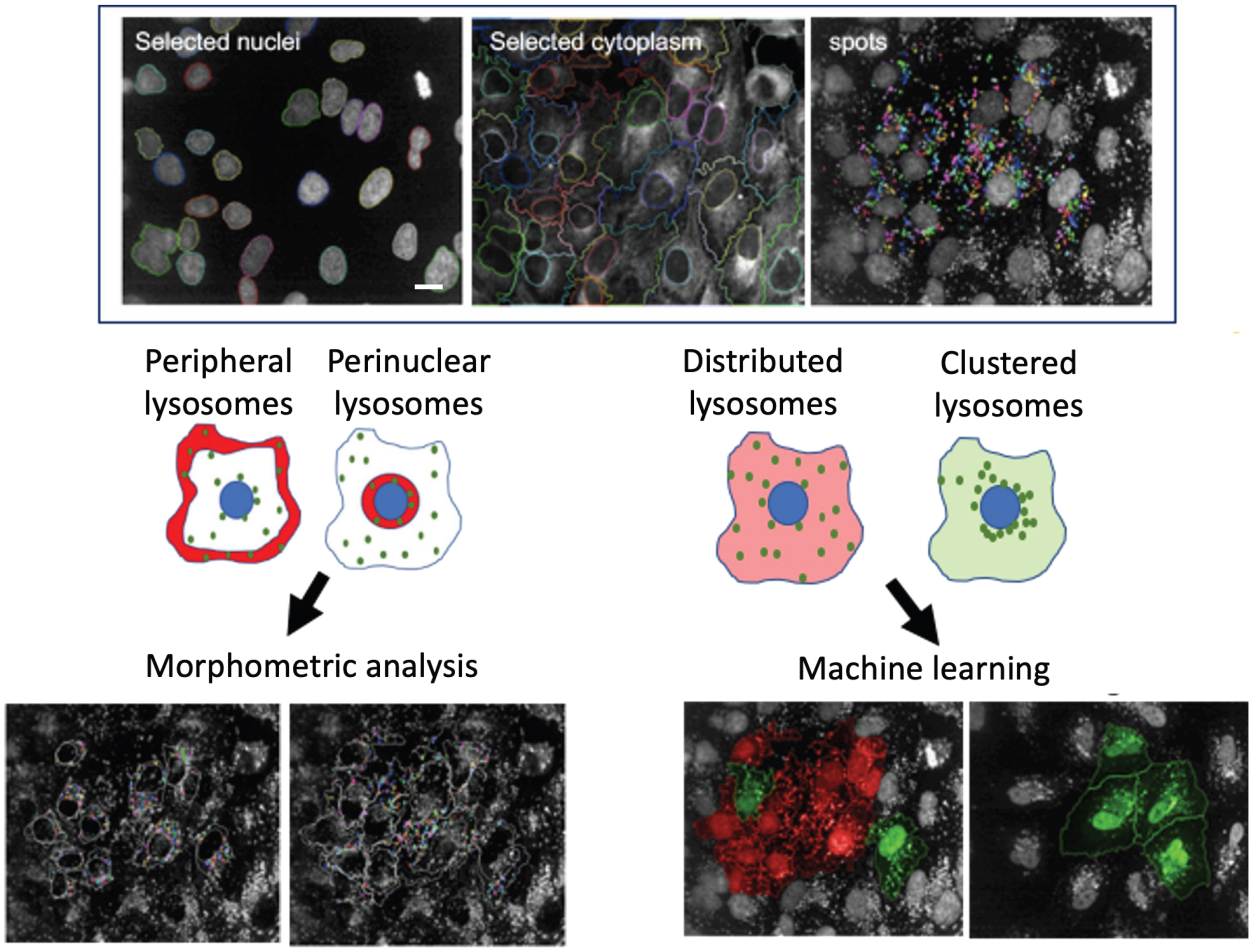
Lysosomal morphometric assay. Results were obtained by the quantitative high content image analysis of normal ARPE‐19 human retinal pigment epithelial cells (WT) and ARPE‐19 Niemann‐Pick C (NPC) 1 KO cells generated by genome editing, as a model of NPC1, a lysosomal storage disease in which lysosomes accumulate cholesterol. The increase in the number of lysosomes and the percentage of cells showing lysosomal aggregation was quantified in NPC1 KO cells and compared to WT cells. Scale bar, 20 μm

In addition, the morphometric analysis of lysosomes and their distribution in normal or pathological conditions can be assessed by HCI using Opera (The Opera Phenix, High‐Content Screening System, PerkinElmer) a phenotypic image‐based ‘machine learning’ script that calculates the percentage of cells showing a clustered lysosomal distribution vs. cells showing lysosomes correctly distributed throughout the cell (Figure 7).

### 4.9 Lysosome motility

#### 4.9.1 Measuring lysosome motility in non‐polarized cells

Lysosome transport along microtubules to the periphery (anterograde movement) is dependent on kinesin motors, while the transport toward the juxtanuclear region (retrograde movement) is dependent on dynein.^208^ In wild‐type cells, lysosomes tend to accumulate in the juxtanuclear region, indicating that the retrograde transport prevails over the anterograde.^208^

Lysosomes should be labelled by combining LysoTracker or dextran (see Sections 4.4.1 and 4.1.1, respectively) with the expression of a fluorescent marker such as GFP, which enables the visualization of the cell outline. Quantification of lysosome motility, direction of movement, displacement and velocity can be done by fluorescence microscopy, typically at 1 frame per second (1 Hz) during 1 to 5 minutes. For this, lysosomes can be labelled with LysoTracker or dextrans.^209^ Tracking of lysosome motility can be performed using Fiji's TrackMate plugin, a single‐particle tracking software, which allows for the segmentation of puncta or roughly spherical objects per image and over time.^210^, ^211^ Individual lysosomes can be followed using a differences of Gaussian (DOG) detector and generic segmentation algorithms. Each punctum is assigned a series of numerical features such as location, radius and mean pixel intensity. A quality‐based threshold can be manually established to eliminate small or dim spots. The HyperStack displayer can be used for spot visualization and the linear assignment problem (LAP) tracker for the particle‐linking algorithm.

#### 4.9.2 Measuring lysosome motility in neurons

Measuring lysosome motility in neurons is challenging because of the size and differentiation of axons and dendrites. Lysosomal movement in axons and dendrites differs because of the microtubule organization. Neuronal microtubules are acentrosomal, with long tracks oriented with the plus end toward axonal terminals and the minus end localized in the cell body. In contrast, microtubule tracks in dendrites are short and of mixed polarity.^212^

Quantification of lysosome motility, the direction of movement, displacement and velocity in axons and dendrites can be done in primary cultures of embryonic mouse or rat hippocampal or cortical neurons.^213^ Adult dorsal root ganglia (sensory neurons),^214^ embryonic peripheral motor neurons^215^ or *Drosophila melanogaster* larval motor neurons^216^ can also be used, depending on the scientific context. Lysosomes are generally identified by overexpressing LAMP1 tagged with a fluorescent protein, but LAMP1 tagged with HALO‐tag (fluorescent upon addition of a ligand^217^) and endogenous tagging of LAMP1 with GFP in induced pluripotent cells (iPSCs), which can be differentiated in neurons, have recently been developed.^218^, ^219^ Alternatively, Bodipy FL‐Pepstatin A (Invitrogen, P12271), an active cathepsin D ligand, can more accurately identify degradative lysosomes.^220^ Furthermore, LysoTracker (see Section 4.4.1) can also be used.^215^

Kymographs (plots of line profiles over time) are especially useful for visualization and quantification of movement along dendrites or axons and can be generated semi‐automatically. For this, the selection and tracing of axonal or dendritic segments is done manually in ‘straightened’ neurites or using a segmented line (Fiji/ImageJ). Then, kymographs are automatically plotted using the ‘reslice’ function (Fiji/ImageJ). If there are multiple segments, the multiple kymograph plugin can be used, enabling a quick visualization of moving lysosomes, as well as stationary ones. Moreover, the direction of movement can be easily identified. For example, if the cell body is positioned at the left, the retrograde moving vesicles are toward the left and the anterograde moving ones to the right in the kymograph. Importantly, the manual analysis of moving lysosomes may introduce biases. Therefore, the Trackmate Fiji plugin can be used to analyze lysosome movement, and parameters such as velocity and distance can be also easily obtained.^217^ Alternatively, MtrackJ (www.imagescience.org/meijering/software/mtrack), a plugin developed for the study of mitochondria movement can be used.^221^ Furthermore, kymograph clear and direct^222^ are ImageJ plugins optimized to generate kymographs and automatically extract the kinematic parameters in retrograde, anterograde and stationary lysosomes.^215^ Another interesting software is Kymoanalyser,^223^ a standalone open‐source software that automatically classifies trajectories and movement parameters that has been used successfully in the case of lysosome transport analysis.^215^

#### 4.9.3 Analysing lysosome motility *in vivo*

For *in vivo* studies (*i.e*., in whole living organisms), the best examples arise from model systems such as *Drosophila melanogaster* and zebrafish (*Danio rerio*). *Drosophila* is a tractable model system with a sophisticated genetic toolkit, ideal for *in vivo* studies, where the labelling of organelles or other vesicles, including live visualization of lysosomes, is rather accessible.^224^ To label lysosomes, there are transgenic flies with genetically‐encoded fluorescent lysosome markers, like LAMP1 and Arl8, which are commonly used for *in vivo* analysis of lysosome morphology and motility. In addition to labelling lysosomes, it is also possible to co‐label other markers of interest (*e.g*., Rab7 and Atg8, to assess endocytic flux).^225^, ^226^, ^227^, ^228^ The expression of fluorescently labelled lysosomes can be performed in virtually any cell type or tissue of interest by using binary systems like the UAS/Gal4, LexAop/LexA or QUAS/QF.^224^, ^229^, ^230^ Lysosome motility can also be analyzed and quantified by expressing fluorescently labelled lysosome markers in specific tissues and using standard methods described above (*e.g*., kymographs, see Section 4.9.2). Live imaging of dissected *ex vivo* tissues is a powerful method to study live organelle dynamics. However, some studies require prolonged imaging or imaging during different developmental timepoints. For this, methods for prolonged imaging of *Drosophila* larvae or adult flies have been developed, and it is now also possible to image intact animals during extended periods.^231^, ^232^, ^233^, ^234^ In *Drosophila* larvae, Kakanj and colleagues developed a method that allows the visualization of tissues, including epidermis, fat body, gut, imaginal discs, neurons, tracheae, muscles and hemocytes, for up to eight consecutive hours.^231^ In adult flies, methods to observe organelle dynamics in the wing or legs, through the cuticle, have also been successfully developed, further expanding the possibilities for live studies.^232^, ^233^, ^234^ The wing, in particular, offers several advantages for live imaging because it is almost transparent, flat, dispensable for survival and bilateral, allowing one side to be subjected to certain treatments while using the contralateral side as control. In summary, *in vivo* imaging of lysosomes in *Drosophila* can be performed in every life cycle stage, and be coupled to other manipulations, including genetic, pharmacological or others. The development of super‐resolution microscopy techniques is also fuelling the quality of *in vivo* analysis of intracellular trafficking.^230^

In zebrafish, valuable tools have been generated such as Rab‐based transgenic lines for Rab5, Rab7 and Rab11 to use in *in vivo* studies of endosome biology in this model organism.^235^ Additionally, specific methods for assessing *in vivo* lysosomal function are now possible, for example, the quantitative monitoring of H_2_S. H_2_S is a physiological mediator of inflammation and is generated in lysosomes of inflamed cells, although its role may be context specific. This gasotransmitter can be monitored using a luminescent probe based on a Ruthenium(II) complex, namely Ru‐MDB (Ru‐4′‐methyl‐[2,2′‐bipyridine]‐4‐yl)methyl 2‐((2,4‐dinitrophenyl)thio)benzoate), which exhibits high specificity and sensitivity for H_2_S and low cytotoxicity.^236^ This enables imaging and flow cytometry analyses of lysosomal H_2_S generation in live cells under drug stimulation. Therefore, this method can lead to a better understanding of the physiological and pathological functions of H_2_S in live organisms. Another useful tool is the generation of fusion proteins of apolipoprotein A‐1 (APOA‐I‐mCherry) to perform cholesterol trafficking studies *in vivo*. This is achieved by feeding TopFluor‐cholesterol (TF‐cholesterol; Avanti Polar Lipids, 810255), a fluorescent cholesterol analogue, to zebrafish. In this way, quantitative microscopy analysis of transgenic zebrafish expressing fluorescently tagged protein markers of late endosomes/lysosomes provides live imaging of cholesterol transport in the intestinal endosomal‐lysosomal system.^237^ Furthermore, a new technology for structured illumination microscopy and imaging applications that are lysosome specific was developed by using biocompatible graphene quantum dots obtained from extracts of neem root.^238^ As there are many similarities between zebrafish and mammalian lipid metabolism, this methodology can potentially be used for showing different aspects of lipoproteins and nutritional biology.

### 4.10 Lysosome immunopurification

The modern ‘omics’ technologies have opened the possibility of characterizing the full molecular inventory of an organelle. This specialized area of science is now referred to as organellar omics and relies on the constant optimization of purification methodologies. Organellar omics allows researchers to gain detailed insight, understand the dynamic nature of organelles and identify not only ‘permanent resident’ proteins but also temporarily associated proteins (interactors) that carry out specialized functions and transitory molecules such as cargo to be processed.

Different protocols have been developed to efficiently isolate lysosomes from other cellular components. Lysosome‐enriched fractions can be obtained by methods based on density gradient media (Iodixanol, Nycodenz, Tyloxapol)^239^ or by density shift upon Triton‐WR1339 treatment.^240^ Nevertheless, two recently developed methods appear to be particularly suitable for omics approaches. The use of superparamagnetic iron oxide nanoparticles (SPIONs) that accumulate in lysosomes allows high‐yield purification of lysosomes that can be subsequently analyzed using proteomic and lipidomic approaches (for more details see Ref. [241]). Another efficient protocol to purify lysosomes has recently been developed in the Sabatini laboratory and named LysoIP.^242^ It is based on lysosome immunopurification, inspired by a similar procedure used for rapid isolation of mitochondria.^243^ The LysoIP uses anti‐human influenza virus hemagglutinin (HA) magnetic beads to immunopurify lysosomes from a variety of cell lines that stably express the lysosomal transmembrane protein 192 (TMEM192) fused to three tandem HA tags. Remarkably, the LysoIP protocol is very fast (~10 minutes to isolate pure and intact lysosomes) and uses buffers that are compatible with liquid chromatography and mass spectrometry (LC/MS), although the overall recovery of lysosomes is low. The plasmids required to generate TMEM192‐HA stably expressing cell lines are available from Addgene (https://www.addgene.org/102930/). TMEM192‐3xHA expression can be evaluated by WB using HA antibody and its lysosomal localization confirmed by monitoring HA and LAMP1 co‐localization by immunofluorescence.^244^ LysoIP can be performed from cell lysates and WB with HA and LAMP1 antibodies used to evaluate immunoprecipitation efficiency. The quality of intact lysosomes should be assessed using an antibody against luminal enzymes such as α‐glucosidase. Lysosome purity is confirmed by the absence of cytosolic components like markers of mitochondria, Golgi apparatus and ER in the isolated lysosomes. However, in some circumstances, residues of intracellular organelles are found in the immunoprecipitated lysosomes, which might be related with organelle‐specific macroautophagy processes. Lysosomal inhibitors can be used to increase the levels of lysosomal substrate detection in the lysosomal fraction. Conversely, the use of autophagy blockers is useful to discriminate autophagy substrates from contaminants. Levels of proteins of interest can be analyzed by WB using specific antibodies, while a more global view of lysosome composition can be obtained by subjecting immunoprecipitated fractions to LC/MS. Novel lysosomal proteins/substrates identified by Lyso‐IP can be subsequently validated by double immunofluorescence analysis using HA/candidate protein antibodies in TMEM192‐3HA expressing cells treated with low doses of bafilomycin (50 nM for 6 hours).^244^

Applications of LysoIP include the characterization of lysosome proteome composition, investigation of lysosomal cargo proteins and metabolites, monitoring of dynamic changes of the lysosome proteome/metabolome upon treatments and unravelling of post‐translational modifications of lysosomal proteins. Overall, this is a rapid and highly reproducible technique. However, several factors ought to be considered before performing this technique. Firstly, the type of cells employed to isolate lysosomes. For example, rat chondrosarcoma (RCS) cells are resistant to mechanical and physical stress and, for this reason, the lysis procedure requires a cell homogenizer. For most cell lines, that is, HeLa (human cervical cancer cell line) or ARPE‐19 (human retinal pigment epithelia cell line), a micro hand‐operated Potter‐Elvehjam homogenizer with a PTFE pestle or 10 passages through a 27G syringe needle are enough to guarantee proper cell lysis and release of the intracellular components. Another factor that should be considered, especially when comparing different experimental settings, is the quantity of lysosomes that can be immunoprecipitated. Different cell lines have different amounts of lysosomes and therefore, the choice of cell line is essential for the final result. Consequently, the total amount of cell lysate used should be optimized considering the average abundance of lysosomes. This calculation becomes critical when the experimental setting employs a single cell line but different treatments that influence lysosome biogenesis or turnover. Another issue is the cross‐contamination with other organelles. This may not be linked to the technical quality of the LysoIP but rather to an increased amount of contact sites between lysosomes and other cytosolic organelles. To evaluate LysoIP efficiency, one can load the flowthrough, also referred to as unbound fraction on sodium dodecyl sulphate‐polyacrylamide gel electrophoresis (SDS‐PAGE) and perform a WB for lysosomal markers, comparing with the input and immunoprecipitated fractions. The greatest advantage of LysoIP is that it does not require sophisticated equipment and it can, therefore, be performed in any cell biology laboratory. Indeed, the available commercial kits for lysosome enrichment are based on density gradient separation, which often requires an ultracentrifugation step. Moreover, LysoIP has a higher yield in terms of lysosome concentration and a lower amount of starting material is required. Noteworthy, a new methodology was recently developed to systematically map the autophagosome content, combining proximity labelling and organelle enrichment with quantitative proteomics.^245^ The expression of a genetically engineered ascorbate peroxidase (APEX2) fused with ATG8 autophagy proteins or with autophagy receptors (p62, NBR1, TOLLIP or OPTN) allows the analysis of substrates and the mechanism of recruitment by the lysosome‐autophagy pathway.

### 4.11 Lysosomal membrane permeabilization

**FIGURE 8:**
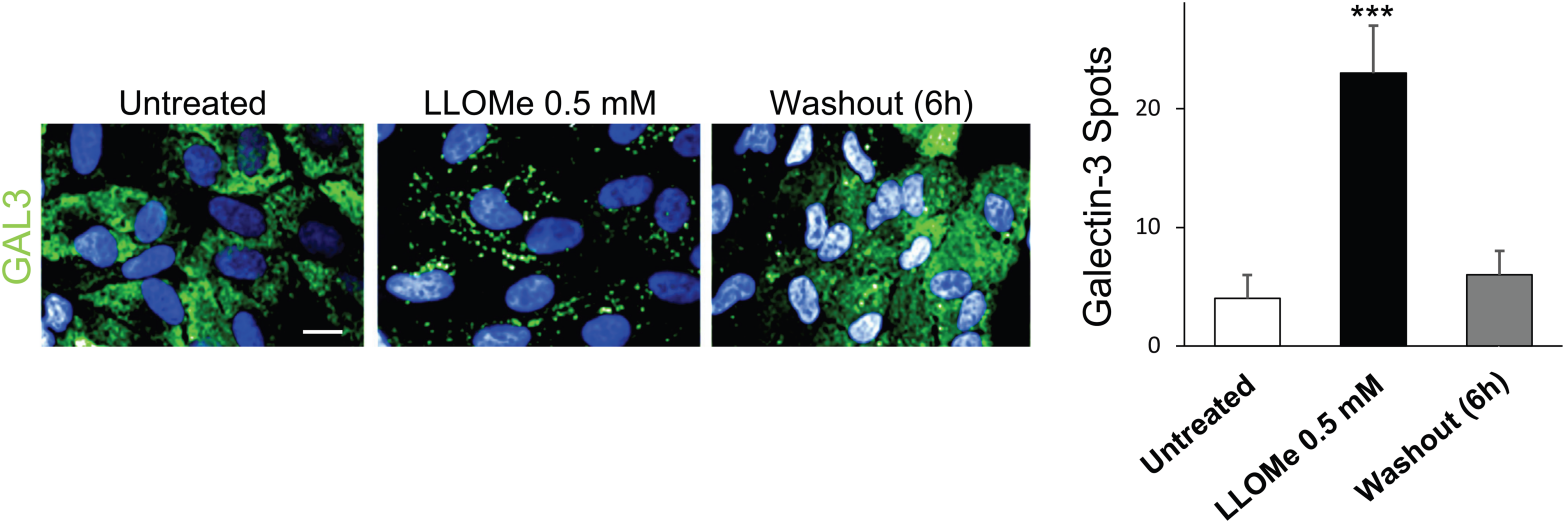
Intracellular galectin 3 (GAL3) accumulation upon treatment with LLOMe. Human ARPE‐19 retinal pigment epithelial cells were stained for GAL3 using a specific antibody (green). Untreated cells show no GAL3 puncta, whereas after 30 minutes of treatment with LLOMe (a potent inducer of lysosome membrane permeabilization), the number of GAL3 spots increase, indicating accumulation. Washout reverts the GAL3 accumulation. The graphs show the number of GAL3 spots per cell. Scale bar, 20 μm

Lysosomal insults or dysfunction often result in LMP, which causes the release of the lysosomal contents, leading to cell dysfunction or death.^246^ Galectin 3 (GAL3) has been shown to be a highly sensitive and easily accessible marker to monitor LMP.^247^ Indeed, lysosomal recruitment of GAL3 upon treatment with l‐Leucyl‐l‐Leucine methyl ester hydrochloride (LLOME, a potent inducer of LMP; MedChemExpress, HY‐129905) is significantly increased, compared to untreated cells.^248^ Therefore, the recruitment of GAL3 to lysosomes can be monitored by immunofluorescence using an antibody anti‐GAL3 from Santa Cruz (SC‐23938). Furthermore, after a 6 hour washout, GAL3‐positive puncta decrease to base levels, indicating that lysosomes can recover from LLOME‐induced damage (Figure 8). Thus, GAL3 recruitment to lysosomes is a robust and reproducible way to compare the effect of different stimuli in LMP, albeit requiring powerful lysosomal injury.^249^

**FIGURE 9:**
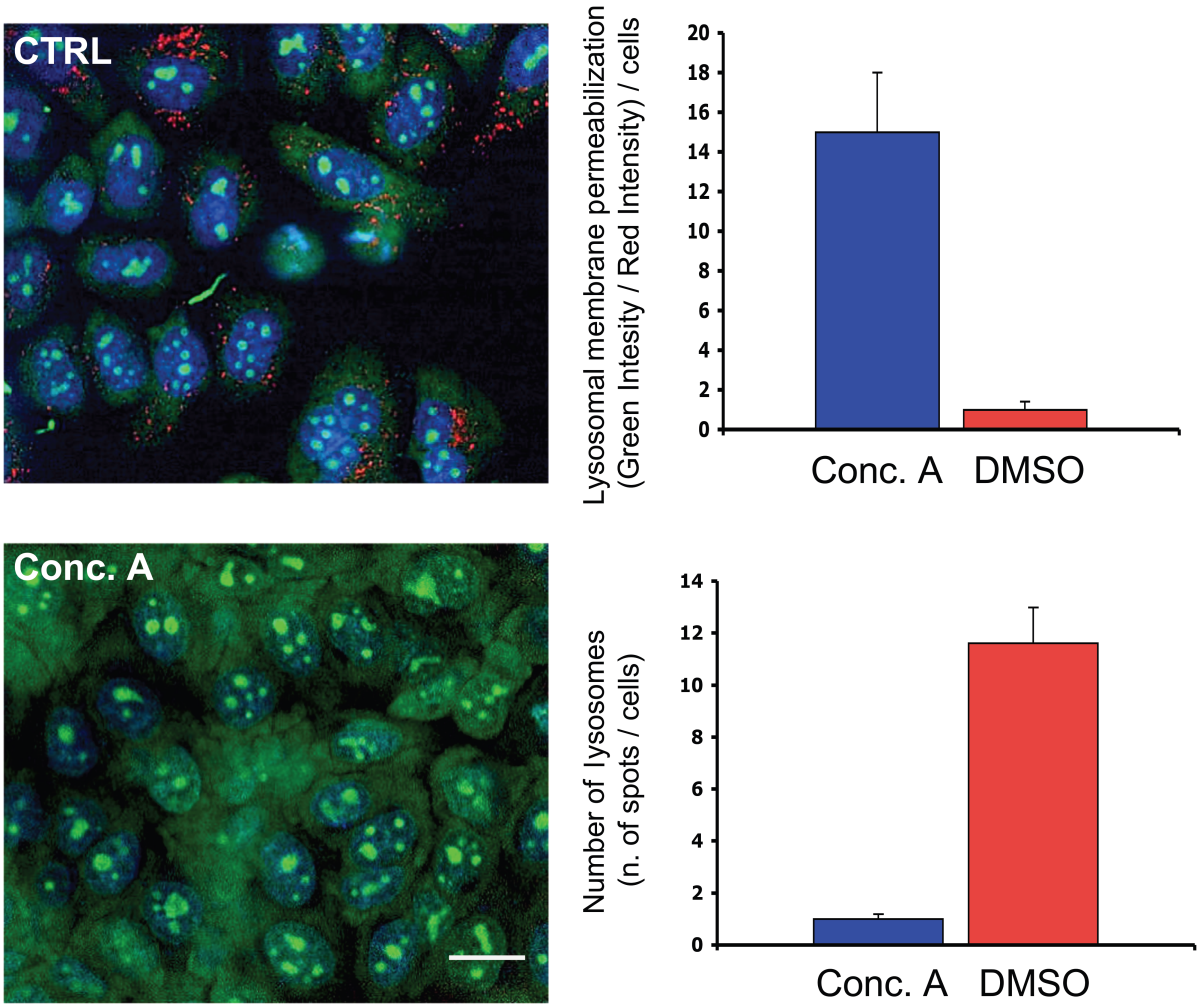
Lysosomal permeabilization assay. In control conditions (CTRL), Acridine Orange stains lysosomes (red puncta), while the treatment with concanamycin A (Conc. A, a specific inhibitor of v‐ATPase activity; 100 nM, 1 hour) dramatically reduces lysosomal staining. With the change in lysosomal pH, there is also an increase in green intensity. The plots show the number of red spots per cell, and the intensity ratio between red and green emission. The assay is developed with live cells and in order to perform a high content analysis, a stable HeLa cell clone expressing the histone protein H2B fused with cyan fluorescent protein (CFP; blue) to detect the nuclei (n = 500 cells per condition) is used. Scale bar, 20 μm

The integrity of the lysosomal membrane can also be inferred by assessing the acidification of this organelle using the lysosomotropic fluorescent probe Acridine Orange (Thermo Fisher, A1301), which emits red light in acidic compartments and green light in alkaline compartments. Thus, the quantification of the red fluorescence can be used to count acidic lysosomes, whereas the reduction in red fluorescence and the increase in green fluorescence can be used to measure lysosomal alkalinization or LMP (Figure 9). For this, live cells should be used because fixed cells are not recommended to use with this dye. The caveats of using Acridine Orange are that it stains DNA, and it is not fixable. Nevertheless, it is a fast and inexpensive assay.

### 4.12 Biochemical analysis of lysosomal hydrolase activity

Lysosomes contain over 70 different acid hydrolases that can catalyze the breakdown of proteins, lipids, polysaccharides, DNA and RNA.^250^ Mutations in the genes encoding these enzymes underlie more than 30 LSDs.^251^ Lysosomal hydrolases typically present an optimum acidic pH, being most active at lysosomal pH (4.5‐5.0) and nearly inactive at neutral cytosolic pH.^251^

Historically, the measurement of lysosomal hydrolase activity has been the gold‐standard for LSD diagnosis,^252^ being typically followed by confirmatory molecular identification of disease‐causing mutations.^16^ In the early 2000s, the concept of using dried blood spots (DBS) for testing lysosomal enzyme activity was introduced,^253^ which allowed the establishment of new‐born screening protocols with multiplex assays for simultaneous diagnosis of several LSDs.^254^ Besides their clinical application, these assays are also routinely applied in research laboratories to investigate lysosome biogenesis and dysfunction in multiple human pathologies.

#### 4.12.1 Assessing lysosomal hydrolase activity *in vitro*

Lysosomal hydrolase activity can be easily assayed in the blood (serum/plasma or leukocytes) and other biological samples when the enzyme is sufficiently abundant.^255^ Radiolabelled natural substrates were the first type of probes developed for these assays and, because of their high specificity and signal‐to‐noise ratio, they are still preferred in testing multiple hydrolases (*e.g*., galactocerebrosidase, acid sphingomyelinase and lysosomal acid lipase).^252^ Other options include tandem MS assays for multiplex detection of several enzymatic activities, employing specific substrates and internal standards, as historically used in new‐born screening protocols in DBS.^252^, ^256^ However, most lysosomal enzymes have been assayed with the widely used commercially available synthetic 4‐methylumbelliferone (4‐MU, Sigma, M1381) substrates^257^ (see Table S3). Regardless of the technique chosen, assaying lysosomal hydrolase activity requires specific optimal conditions (co‐factors, pH, detergent, inhibitors, etc.) for each enzyme. For example, the activity of some sphingolipid degrading enzymes, such as gluco‐ and galactocerebrosidase, requires the activation by small lysosomal glycoproteins (saposin C and A, respectively).^81^ This requirement must be taken into consideration when setting up an *in vitro* assay in which the enzymes are not in direct contact with these proteins. In the case of GBA, the activation can be achieved by using taurocholate in the assay buffer.^258^

In 4‐MU‐based assays, when optimal conditions are defined, the enzyme will hydrolyze a fraction of the substrate containing the 4‐methylumbelliferyl moiety, resulting in the release of the 4‐MU group, which is highly fluorogenic at basic pH (pH 10‐11). Alkaline buffers are thus routinely used to quench and stop the enzymatic reaction, improving the fluorescence yield. The enzymatic activity can then be calculated as a direct proportion of the fluorescence intensity.^259^ Generally, 4‐MU based assays are simple and sensitive, so they are widely used in clinical diagnosis, as well as in fundamental research. Alternative artificial substrates exist with other reporters, for example the chromogenic substrate p‐nitrophenol (Supelco, 48 459), which is cheaper but 1000‐fold less sensitive than 4‐MU (Table 6). The major drawback of 4‐MU based substrates is their high pKa (7.8), which requires the addition of alkaline buffers to stop the reaction, hampering the use of continuous assays.^260^ The most recent generation of fluorophores have acidic pKa values and are thus better suited for continuous assays, besides being less prone to autofluorescence and having nanomole sensitivity (high quantum yield).^261^ These include the red fluorogenic substrate resorufin (Sigma, 73144) and the green fluorescein (Sigma, F2456) (Table 6). Finally, natural substrates coupled to a fluorophore such as BODIPY are also commonly used in combination with methods that involve more labour‐intensive high‐performance liquid chromatography (HPLC) or less sensitive thin‐layer chromatography (TLC) techniques.

**TABLE 6:** Chromophores and fluorophores commonly used in artificial substrates for lysosomal hydrolases

Category	Compound
Chromophores	4‐nitrophenol (4‐NP)
	4‐nitrocatechol (4‐NC)
	2‐naphthol (2‐NP)
	p‐nitroanilide
	5‐bromo‐4‐chloro‐3‐hydroxyindole
Fluorophores	4‐methylumbelliferyl (4‐MU)
	7‐amino‐4‐methylcoumarin (AMC)
	7‐hydroxy‐9*H*‐(1,3‐dichloro‐9,9‐dimethylacridin‐2‐one)) (DDAO)
	7‐methoxycoumarin‐4‐yl)acetyl (MCA) / 2,4‐dinitrophenyl (Dnp)
	Lucifer yellow/4‐(4‐Dimethylaminophenylazo)benzenesulfonyl) (Dabsyl)
	Fluorescein Resofurin
	ELF 97
	Cresyl violet
	Nitrobenzoxadiazole (NBD)

The downsides of using artificial substrates include the fact that these assays do not fully reflect the physiological conditions that exist in cells. The *in vivo* enzymatic activity may thus be distinct from that assayed in cell or tissue preparations. Indeed, these assays are usually unable to fully mimic the local lysosomal environment regarding elements such as co‐factors, chaperones and activators, which are essential for enzyme stability, processing and structure.^252^ In LSDs, hydrolases may also be locally inhibited by the substrates abnormally accumulated inside lysosomes. Additionally, these substrates may be cleaved by other cellular enzymes present in the preparations.^262^ The use of negative controls is thus recommended when testing new samples. These may be genetic controls in which the expression of the hydrolase of interest has been downregulated or abrogated, or enzyme‐specific inhibitors.

#### 4.12.2 Assessing lysosomal hydrolase activity *in vivo*

The limitations associated with *in vitro* activity assays discussed above have triggered the development of more accurate and sensitive tools to monitor lysosomal hydrolases in their natural environment. Substrate‐based probes are broadly available tools that can penetrate the cellular membranes and allow tracking of the activity of lysosomal enzymes *in situ* ^263^ (see Table S3). These substrates are hydrolyzed inside lysosomes, leading to the accumulation of the cleaved fluorescent product in these organelles. Magic Red substrates (cathepsin‐B assay kit: Abcam, ab270772; cathepsin‐L assay kit: Abcam, ab270774) are an example of such compounds, used to assess the intracellular activity of cysteine cathepsins based on the release of a cresyl violet fluorophore upon cleavage.^264^ Substrate‐based probes with a chromogenic group are also available for some lysosomal enzymes, but their use has become less popular in comparison with fluorescent substrates (Table 3). Importantly, deficiency in cathepsin proteases causes LSDs in humans because of the accumulation of partially digested macromolecules and enlarged lysosomes.^265^

Whenever the catalytic mechanism of an enzyme is known, it is possible to develop chemical probes that covalently bind to the enzyme's active site and allow the detection of the enzyme via a reporter moiety grafted to the probe.^266^ These compounds are named activity‐based probes (ABPs), given their dependence on the enzymatic activity rather than molecular affinity alone.^267^ ABPs are generally composed by a tag, a linker and an active site binding motif incorporating a reactive group, also called ‘warhead’.^268^ For successfully monitoring the activity of lysosomal enzymes *in vivo* and *in situ*, ABPs must be able to efficiently reach lysosomes and fluoresce within the acidic environment of the lysosome lumen.^267^ Fluorophores with low pKa values (*e.g*., BODIPY, DAMP, Cy5.5, etc.) have optimal fluorescence in the low pH of the lysosome and have been commonly used for labelling lysosomal hydrolases.^263^, ^267^ Moreover, while fluorogenic substrates make it possible to detect the activity of lysosomal hydrolases in living cells or tissue sections, ABPs have a broader range of applications because of their ability to covalently bind the enzyme's active site. The sensitive and specific mechanism of binding of ABPs enables the visualization of endogenous enzymes in complex samples such as cell lysates and tissue homogenates, as well as in whole cells, tissue sections or even living organisms, in the case of ABPs with infrared tags detected by magnetic resonance imaging.^267^ By combining light microscopy and EM, it is even possible to visualize active GBA enzyme in individual lysosomes of cells exposed to the appropriate ABP.^269^ These applications have proven valuable for confirming the diagnosis of LSDs^270^ and other pathologies such as cancer,^263^ as well as in studies addressing the impact of certain mutations in the enzymatic activity or the efficacy of therapeutic approaches.^271^, ^272^ Numerous other uses can be envisioned for a specific ABP, from crystallography studies on mutant enzymes to high‐throughput screening for inhibitors or interactors.^273^ In particular, probes with a tag suited for pull‐down experiments such as biotin, can be employed for affinity‐enrichment and identification of the labelled targets and/or interactors by proteomics, a technique termed activity‐based protein profiling (ABPP).^267^

A broad range of fluorogenic substrates to monitor lysosomal hydrolase activity is now commercially available, making them accessible and easy‐to‐use tools. Magic Red substrates, in particular, have recently been adapted for use in HCI analysis protocols to monitor lysosome function in LSD models by using Opera and Operetta systems (PerkinElmer).^274^ A caveat of these tools is their relatively low specificity, as they may be cleaved by multiple proteases. Magic Red substrates may thus be useful tools to monitor general lysosomal protease activity in living cells but comparatively unsuited for accurately assessing a specific cathepsin protease. In general, fluorescence imaging may also be harmful to living cells through the generation of oxygen radicals, while coloured enzyme products are relatively harmless.^264^ ABPs, on the other hand, can be quite sensitive and specific tools for monitoring the activity of a given lysosomal hydrolase, as has been shown for multiple glycosidases and other hydrolases. On the downside, ABPs are often not commercially available, and their use may be patented, making them less readily available tools for general use. The suicide‐inhibitor mechanism of binding of these probes also implies the abrogation of the enzymatic activity, which may have a physiological impact on the model organism when sustained over longer periods. The spectrum of ABPs available is rapidly increasing (see Table S3) but sensitive and specific ABPs for some lysosomal hydrolases are yet to be developed.

### 4.13 Lysosome exocytosis

Although lysosomes are generally regarded as terminal degradative organelles, they can also undergo exocytosis, releasing their luminal contents.^15^, ^275^ Lysosome exocytosis plays a pivotal role in several biological processes such as plasma membrane repair, antigen presentation, bone resorption and tissue remodelling.^276^, ^277^ Moreover, this is a Ca^2+^‐regulated process that occurs in almost all cell types. Therefore, Ca^2+^ ionophores like ionomycin (Sigma, I9657)^201^, ^278^ or agonists of the Ca^2+^ channel present in the lysosome membrane —TRPML1— such as ML‐SA1 (Sigma, SML0627) and MK6‐83 (AbMole, M10315), are commonly used to induce lysosome exocytosis.^279^ Furthermore, the extracellular acidic environment found in tumours^280^, ^281^ and altered expression of genes that regulate lysosome positioning and motility^282^ can also lead to an increase in lysosome exocytosis.

One of the most commonly used methods to monitor lysosome exocytosis is the detection of lysosomal membrane proteins, such as LAMP1, at the plasma membrane. When lysosomes fuse with the plasma membrane, the luminal domain of LAMP1 is exposed. Thus, detection of LAMP1 with a monoclonal antibody for a luminal epitope of the protein (*e.g*., anti‐LAMP1 H4A3) provides a good readout of lysosome exocytosis.^279^, ^283^ Cell surface LAMP1 can be measured by flow cytometry^201^, ^278^ or fluorescence microscopy.^279^, ^284^ When live cells are used, cell should be kept at 4°C to avoid antibody endocytosis. To preclude the detection of the intracellular pool of LAMP1, the staining should be performed in non‐permeabilized cells^275^, ^285^ and when using live cells, these should be fixed after labelling with LAMP1 antibody at 4°C.^284^

The detection of released lysosomal enzymes in the extracellular milieu is also often used to evaluate lysosome exocytosis. The unprocessed and processed (mature) forms of lysosomal enzymes can be detected in the extracellular media, by WB, using specific antibodies that are commercially available (*e.g*., anti‐cathepsin D from Abcam, ab6313; anti‐cathepsin B from R&D Systems, AF953; and anti‐cathepsin L from R&D Systems, AF952).^286^, ^287^ The activity of secreted lysosomal enzymes, such as β‐hexosaminidase (β‐hex), acid‐phosphatase, β‐N‐acerylglucosaminidase, β‐galactosidase, acid sphingomyelinase and cathepsins can be measured, using appropriate fluorometric or colorimetric substrates.^201^, ^278^, ^288^, ^289^, ^290^ For example, the activity of secreted β‐hex can be measured by incubating cell supernatants and cell lysates with the substrate 4‐MU‐β‐d‐glucosaminide (4‐MU‐β‐D‐GlcNAc, Glycosynth, 44007). The detection of LAMP1 at the plasma membrane can be easily performed and does not require much expertise. However, LAMP1 is also present in late endosomes, which can also be exocytosed. Thus, measuring lysosomal enzyme release and activity is a more specific method to monitor lysosome exocytosis. Moreover, it is a quantitative method that can be easily implemented. The major limitation is that some cells release low amounts of lysosomal enzymes.

#### 4.13.1 Visualizing lysosome exocytosis

Fusion of lysosomes with the plasma membrane can be visualized in live cells by total internal reflection fluorescence (TIRF) microscopy. For this purpose, it is necessary to establish cell lines expressing, preferably stably, lysosomal markers conjugated with pH‐sensitive probes such as pHluorin. The fluorescence signal from pHluorin is quenched in acidic environment. As lysosomes undergo fusion with the plasma membrane, pHluorin contacts the extracellular medium neutral pH and the fluorescence signal can be detected as a flash of light. Therefore, the pHluorin signal is a good readout of lysosome exocytic events.^287^, ^291^, ^292^ TIRF microscopy allows the visualization of cellular processes that occur near the plasma membrane. However, TIRF systems are not available in all research institutions and data interpretation is not always easy. Moreover, it can only be done in live cells.

EM is also a powerful tool for the analysis of lysosome exocytosis. This approach must be combined with tannic acid treatment, which allows exocytic structures to be arrested at the stage of their docking/fusion with the plasma membrane.^288^, ^293^, ^294^ Further immuno‐EM labelling for LAMP1 helps discriminate lysosomes from other exocytic organelles captured by tannic acid close to the plasma membrane. Therefore, an increase in the density of LAMP1‐positive organelles along the plasma membrane indicates enhanced lysosome exocytosis.^288^ This method enables not only the visualization of the endolysosomal structure of interest with high resolution but also the evaluation of the degree of its maturation or dysfunction (in the case of LSDs). However, its throughput is limited to a couple of dozen cells per specimen. Moreover, EM requires specific and expensive equipment, expertise and trained users.

## 5 FUTURE DIRECTIONS OF THE RESEARCH ON LYSOSOMES

Even though lysosomes were discovered more than 70 years ago, essential concepts regarding their function and life cycle were only uncovered in recent years. This was possible because of methodological advances such as imaging techniques that allow the visualization of the dynamics of this organelle in live cells. We now have a better understanding of the heterogeneity of different lysosome populations and their characteristics. However, we are still starting to understand their functions in cell physiology. New insights will arise from future technological developments as the resolution of imaging techniques, both in time and space, further increases.

The outdated view of lysosomes as simply degradative organelles has been changed by the finding that they play a central role in several other cellular functions, such as nutrient sensing, intracellular signalling and metabolism. Indeed, lysosomes are essential for many aspects of cell physiology and failure in their functions can result in cellular ageing and age‐associated phenotypes such as cell senescence. Therefore, the reestablishment of lysosome functions (*i.e*., ‘lysosome rejuvenation’) might represent a promising anti‐ageing strategy. In addition, a deeper understanding of lysosome biology can provide novel therapeutic strategies for LSDs and other lysosomal disorders, as well as for age‐related degenerative diseases. In order to achieve these goals, lysosome research must continue, using cutting edge methodology. It seems inevitable that the lysosome will continue to surprise us for many years to come.

## CONFLICT OF INTEREST

The authors declare no potential conflict of interest.

6

### PEER REVIEW

The peer review history for this article is available at https://publons.com/publon/10.1111/tra.12839.

## Supporting information


**Data S1** Supporting Information.

## References

[1] Bainton DF . The discovery of lysosomes. J Cell Biol. 1981;91:66s‐76s. 7033245 10.1083/jcb.91.3.66s PMC2112804

[2] De Duve C . The separation and characterization of subcellular particles. Harvey Lect. 1965;59:49‐87. 5337823

[3] Castro‐Obregon S . The discovery of lysosomes and autophagy. Nat Educ. 2010;3:49.

[4] Novikoff AB , Beaufay H , De Duve C . Electron microscopy of lysosomerich fractions from rat liver. J Biophys Biochem Cytol. 1956;2:179‐184. 13357540 PMC2229688

[5] Straus W . Isolation and biochemical properties of droplets from the cells of rat kidney. J Biol Chem. 1954;207:745‐755. 13163061

[6] Cohn ZA . The fate of bacteria within phagocytic cells. I. The degradation of isotopically labeled bacteria by polymorphonuclear leucocytes and macrophages. J Exp Med. 1963;117:27‐42. 14022146 10.1084/jem.117.1.27 PMC2180432

[7] Smith RE , Farquhar MG . Lysosome function in the regulation of the secretory process in cells of the anterior pituitary gland. J Cell Biol. 1966;31:319‐347. 19866704 10.1083/jcb.31.2.319 PMC2107048

[8] Goldstein JL , Anderson RG , Brown MS . Coated pits, coated vesicles, and receptor‐mediated endocytosis. Nature. 1979;279(5715):679‐685. 221835 10.1038/279679a0

[9] Silverstein SC , Steinman RM , Cohn ZA . Endocytosis. Annu Rev Biochem. 1977;46:669‐722. 332066 10.1146/annurev.bi.46.070177.003321

[10] Caragata EP , Tikhe CV , Dimopoulos G . Curious entanglements: interactions between mosquitoes, their microbiota, and arboviruses. Curr Opin Virol. 2019;37:26‐36. 31176069 10.1016/j.coviro.2019.05.005 PMC6768729

[11] Klionsky DJ . Autophagy revisited: a conversation with Christian de Duve. Autophagy. 2008;4:740‐743. 18567941 10.4161/auto.6398

[12] Ohsumi Y . Historical landmarks of autophagy research. Cell Res. 2014;24:9‐23. 24366340 10.1038/cr.2013.169 PMC3879711

[13] Martello A , Platt FM , Eden ER . Staying in touch with the endocytic network: the importance of contacts for cholesterol transport. Traffic. 2020;21:354‐363. 32129938 10.1111/tra.12726 PMC8650999

[14] Wong YC , Ysselstein D , Krainc D . Mitochondria‐lysosome contacts regulate mitochondrial fission via RAB7 GTP hydrolysis. Nature. 2018;554:382‐386. 29364868 10.1038/nature25486 PMC6209448

[15] Andrews NW . Regulated secretion of conventional lysosomes. Trends Cell Biol. 2000;10:316‐321. 10884683 10.1016/s0962-8924(00)01794-3

[16] Platt FM , d'Azzo A , Davidson BL , Neufeld EF , Tifft CJ . Lysosomal storage diseases [Erratum in: Nat Rev Dis Primers. 2019, 17:34]. Nat Rev Dis Primers. 2018;4:27. 30275469 10.1038/s41572-018-0025-4

[17] Hers HG . α‐Glucosidase deficiency in generalized glycogen‐storage disease (Pompe's disease). Biochem J. 1963;86:11. 13954110 10.1042/bj0860011 PMC1201703

[18] Ballabio A , Bonifacino JS . Lysosomes as dynamic regulators of cell and organismal homeostasis. Nat Rev Mol Cell Biol. 2019;2:101‐118. 10.1038/s41580-019-0185-4 31768005

[19] Bouhamdani N , Comeau D , Turcotte S . A compendium of information on the lysosome. Front Cell Dev Biol. 2021;9:798262. 34977038 10.3389/fcell.2021.798262 PMC8714965

[20] Bright NA , Gratian MJ , Luzio JP . Endocytic delivery to lysosomes mediated by concurrent fusion and kissing events in living cells. Curr Biol. 2005;15:360‐365. 15723798 10.1016/j.cub.2005.01.049

[21] Futter CE , Pearse A , Hewlett LJ , Hopkins CR . Multivesicular endosomes containing internalized EGF‐EGF receptor complexes mature and then fuse directly with lysosomes. J Cell Biol. 1996;132:1011. 8601581 10.1083/jcb.132.6.1011 PMC2120766

[22] Bright NA , Davis LJ , Luzio JP . Endolysosomes are the principal intracellular sites of acid hydrolase activity. Curr Biol. 2016;26:2233‐2245. 27498570 10.1016/j.cub.2016.06.046 PMC5026700

[23] Cheng XT , Xie YX , Zhou B , Huang N , Farfel‐Becker T , Sheng ZH . Characterization of LAMP1‐labeled nondegradative lysosomal and endocytic compartments in neurons. J Cell Biol. 2018;217:3127‐3139. 29695488 10.1083/jcb.201711083 PMC6123004

[24] Swanson J , Bushnell A , Silverstein SC . Tubular lysosome morphology and distribution within macrophages depend on the integrity of cytoplasmic microtubules (phorbol ester/10‐nm filaments/cathepsin L/acid phosphatase). Cell Biol. 1987;84:1921‐1925. 10.1073/pnas.84.7.1921 PMC304553 3550801

[25] Chapel A , Kieffer‐Jaquinod S , Sagné C , et al. An extended proteome map of the lysosomal membrane reveals novel potential transporters. Mol Cell Proteomics. 2013;12:1572‐1588. 23436907 10.1074/mcp.M112.021980 PMC3675815

[26] Lübke T , Lobel P , Sleat DE . Proteomics of the lysosome. Biochim Biophys Acta Mol Cell Res. 2009;1793:625‐635. 10.1016/j.bbamcr.2008.09.018 PMC2684028 18977398

[27] Schröder BA , Wrocklage C , Hasilik A , Saftig P . The proteome of lysosomes. Proteomics. 2010;10:4053‐4076. 20957757 10.1002/pmic.201000196

[28] Cuervo AM , Dice JF . A receptor for the selective uptake and degradation of proteins by lysosomes. Science. 1996;273:501‐503. 8662539 10.1126/science.273.5274.501

[29] Shin HJR , Kim H , Oh S , et al. AMPK‐SKP2–CARM1 signalling cascade in transcriptional regulation of autophagy. Nature. 2016;534:553. 27309807 10.1038/nature18014 PMC5568428

[30] Johnson DE , Ostrowski P , Jaumouillé V , Grinstein S . The position of lysosomes within the cell determines their luminal pH. J Cell Biol. 2016;212:677. 26975849 10.1083/jcb.201507112 PMC4792074

[31] Luzio JP , Hackmann Y , Dieckmann NM , Griffiths GM . The biogenesis of lysosomes and lysosome‐related organelles. Cold Spring Harb Perspect Biol. 2014;6:a016840. 25183830 10.1101/cshperspect.a016840 PMC4142962

[32] Yang C , Wang X . Lysosome biogenesis: regulation and functions. J Cell Biol. 2021;220:e202102001. 33950241 10.1083/jcb.202102001 PMC8105738

[33] Chen Y , Yu L . Recent progress in autophagic lysosome reformation. Traffic. 2017;18:358‐361. 28371052 10.1111/tra.12484

[34] Li Y , Mcphee CK , Zheng L , et al. Autophagy termination and lysosome reformation regulated by mTOR. Nature. 2010;465:942‐946. 20526321 10.1038/nature09076 PMC2920749

[35] Steinberg BE , Touret N , Vargas‐Caballero M , Grinstein S . In situ measurement of the electrical potential across the phagosomal membrane using FRET and its contribution to the proton‐motive force. Proc Natl Acad Sci U S A. 2007;104:9523‐9528. 17517624 10.1073/pnas.0700783104 PMC1890527

[36] Steinberg BE , Huynh KK , Brodovitch A , et al. A cation counterflux supports lysosomal acidification. J Cell Biol. 2010;189:1171‐1186. 20566682 10.1083/jcb.200911083 PMC2894458

[37] Huizing M , Anikster Y , Gahl WA . Hermansky–Pudlak syndrome and related disorders of organelle formation. Traffic. 2000;1:823‐835. 11208073 10.1034/j.1600-0854.2000.011103.x

[38] Dell'Angelica EC , Shotelersuk V , Aguilar RC , Gahl WA , Bonifacino JS . Altered trafficking of lysosomal proteins in Hermansky‐Pudlak syndrome due to mutations in the β3A subunit of the AP‐3 adaptor. Mol Cell. 1999;3:11‐21. 10024875 10.1016/s1097-2765(00)80170-7

[39] Novak EK , Swank RT . Lysosomal dysfunctions associated with mutations at mouse pigment genes. Genetics. 1979;92:189. 115747 10.1093/genetics/92.1.189 PMC1213941

[40] Orlow SJ . Melanosomes are specialized members of the lysosomal lineage of organelles. J Invest Dermatol. 1995;105:3‐7. 7615972 10.1111/1523-1747.ep12312291

[41] Griscelli C , Durandy A , Guy‐Grand D , Daguillard F , Herzog C , Prunieras M . A syndrome associating partial albinism and immunodeficiency. Am J Med. 1978;65:691‐702. 707528 10.1016/0002-9343(78)90858-6

[42] Peters PJ , Borst J , Oorschot V , et al. Cytotoxic T lymphocyte granules are secretory lysosomes, containing both perforin and granzymes. J Exp Med. 1991;173:1099‐1109. 2022921 10.1084/jem.173.5.1099 PMC2118839

[43] Lui‐Roberts WW , Collinson LM , Hewlett LJ , Michaux G , Cutler DF . An AP‐1/clathrin coat plays a novel and essential role in forming the Weibel‐Palade bodies of endothelial cells. J Cell Biol. 2005;170:627‐636. 16087708 10.1083/jcb.200503054 PMC2171491

[44] Delevoye C , Marks MS , Raposo G . Lysosome related organelles as functional adaptations of the endolysosomal system. Curr Opin Cell Biol. 2019;59:147. 31234051 10.1016/j.ceb.2019.05.003 PMC6726539

[45] Bowman SL , Bi‐Karchin J , Le L , Marks MS . The road to LROs: insights into lysosome‐related organelles from Hermansky‐Pudlak syndrome and other rare diseases. Traffic. 2019;20:404. 30945407 10.1111/tra.12646 PMC6541516

[46] Le L , Sirés‐Campos J , Raposo G , Delevoye C , Marks MS . Melanosome biogenesis in the pigmentation of mammalian skin. Integr Comp Biol. 2021;61:1517‐1545. 34021746 10.1093/icb/icab078 PMC8516112

[47] Hume AN , Collinson LM , Hopkins CR , et al. The leaden gene product is required with Rab27a to recruit myosin Va to melanosomes in melanocytes. Traffic. 2002;3:193‐202. 11886590 10.1034/j.1600-0854.2002.030305.x

[48] McCormack JJ , Lopes da Silva M , Ferraro F , Patella F , Cutler DF . Weibel‐Palade bodies at a glance. J Cell Sci. 2017;130:3611‐3617. 29093059 10.1242/jcs.208033

[49] Karampini E , Bierings R , Voorberg J . Orchestration of primary hemostasis by platelet and endothelial lysosome‐related organelles. Arterioscler Thromb Vasc Biol. 2020;40:1441‐1453. 32375545 10.1161/ATVBAHA.120.314245

[50] Chen CH , Lo RW , Urban D , Pluthero FG , Kahr WH . α‐Granule biogenesis: from disease to discovery. Platelets. 2017;28:147‐154. 28277061 10.1080/09537104.2017.1280599

[51] Sharda A , Flaumenhaft R . The life cycle of platelet granules. F1000Research. 2018;7:236. 29560259 10.12688/f1000research.13283.1 PMC5832915

[52] Dupuis A , Bordet J‐C , Eckly A , Gachet C . Platelet δ‐storage pool disease: an update. J Clin Med. 2020;9:2508. 10.3390/jcm9082508 PMC7466064 32759727

[53] Albrecht S , Usmani SM , Dietl P , Wittekindt OH . Plasma membrane trafficking in alveolar type II cells. Cell Physiol Biochem. 2010;25:81‐90. 20054147 10.1159/000272053

[54] Faigle W , Raposo G , Tenza D , et al. Deficient peptide loading and MHC class II endosomal sorting in a human genetic immunodeficiency disease: the Chediak‐Higashi syndrome. J Cell Biol. 1998;141:1121‐1134. 9606205 10.1083/jcb.141.5.1121 PMC2137185

[55] Roche PA , Furuta K . The ins and outs of MHC class II‐mediated antigen processing and presentation. Nat Rev Immunol. 2015;15:203‐216. 25720354 10.1038/nri3818 PMC6314495

[56] Melo RCN , Weller PF . Contemporary understanding of the secretory granules in human eosinophils. J Leukoc Biol. 2018;104:85. 29749658 10.1002/JLB.3MR1217-476R PMC6013358

[57] Fujiwara T , Ye S , Castro‐Gomes T , et al. PLEKHM1/DEF8/RAB7 complex regulates lysosome positioning and bone homeostasis. JCI Insight. 2016;1:86330. 27777970 10.1172/jci.insight.86330 PMC5070964

[58] Matsumoto N , Sekiya M , Tohyama K , et al. Essential role of the a3 isoform of V‐ATPase in secretory lysosome trafficking via Rab7 recruitment. Sci Rep. 2018;8:1‐18. 29712939 10.1038/s41598-018-24918-7 PMC5928161

[59] Sheshachalam A , Srivastava N , Mitchell T , Lacy P , Eitzen G . Granule protein processing and regulated secretion in neutrophils. Front Immunol. 2014;5:448. 25285096 10.3389/fimmu.2014.00448 PMC4168738

[60] Khawar MB , Gao H , Li W . Mechanism of acrosome biogenesis in mammals. Front Cell Dev Biol. 2019;7:195. 31620437 10.3389/fcell.2019.00195 PMC6759486

[61] Ito C , Toshimori K . Acrosome markers of human sperm. Anat Sci Int. 2016;91:128‐142. 26748928 10.1007/s12565-015-0323-9

[62] Wankel B , Ouyang J , Guo X , et al. Sequential and compartmentalized action of Rabs, SNAREs, and MAL in the apical delivery of fusiform vesicles in urothelial umbrella cells. Mol Biol Cell. 2016;27:1621. 27009205 10.1091/mbc.E15-04-0230 PMC4865319

[63] Ellis K , Bagwell J , Bagnat M . Notochord vacuoles are lysosome‐related organelles that function in axis and spine morphogenesis. J Cell Biol. 2013;200:667‐679. 23460678 10.1083/jcb.201212095 PMC3587825

[64] Griffiths GM . Albinism and immunity: what's the link? Curr Mol Med. 2002;2:479‐483. 12125813 10.2174/1566524023362258

[65] Dieckmann NM , Frazer GL , Asano Y , Stinchcombe JC , Griffiths GM . The cytotoxic T lymphocyte immune synapse at a glance. J Cell Sci. 2016;129:2881‐2886. 27505426 10.1242/jcs.186205

[66] Saftig P , Klumperman J . Lysosome biogenesis and lysosomal membrane proteins: trafficking meets function. Nat Rev Mol Cell Biol. 2009;10:623‐635. 19672277 10.1038/nrm2745

[67] De Risi M , Torromino G , Tufano M , et al. Mechanisms by which autophagy regulates memory capacity in ageing. Aging Cell. 2020;19:e13189. 32729663 10.1111/acel.13189 PMC7511873

[68] Brown WJ , Goodhouse J , Farquhar MG . Mannose‐6‐phosphate receptors for lysosomal enzymes cycle between the Golgi complex and endosomes. J Cell Biol. 1986;103:1235‐1247. 2945825 10.1083/jcb.103.4.1235 PMC2114320

[69] Witte MD , Kallemeijn WW , Aten J , et al. Ultrasensitive in situ visualization of active glucocerebrosidase molecules. Nat Chem Biol. 2010;6:907‐913. 21079602 10.1038/nchembio.466

[70] Pols MS , Van Meel E , Oorschot V , et al. hVps41 and VAMP7 function in direct TGN to late endosome transport of lysosomal membrane proteins. Nat Commun. 2013;4:1361. 23322049 10.1038/ncomms2360

[71] Baba K , Kuwada S , Nakao A , et al. Different localization of lysosomal‐associated membrane protein 1 (LAMP1) in mammalian cultured cell lines. Histochem Cell Biol. 2020;1534:199‐213. 10.1007/s00418-019-01842-z 31907597

[72] Chaurra A , Gutzman BM , Taylor E , Ackroyd PC , Christensen KA . Lucifer yellow as a live cell fluorescent probe for imaging water transport in subcellular organelles. Appl Spectrosc. 2011;65:20‐25. 21211149 10.1366/10-06095

[73] Festa BP , Berquez M , Gassama A , et al. OCRL deficiency impairs endolysosomal function in a humanized mouse model for Lowe syndrome and Dent disease. Hum Mol Genet. 2019;28:1931‐1946. 30590522 10.1093/hmg/ddy449 PMC6548226

[74] Ellegaard AM , Jäättelä M , Nylandsted J . Visualizing lysosomal membrane permeabilization by fluorescent dextran release. Cold Spring Harb Protoc. 2015;1:900‐903. 10.1101/pdb.prot086173 26430253

[75] Liang C , Lee J , Inn K‐S , et al. Beclin1‐binding UVRAG targets the class C Vps complex to coordinate autophagosome maturation and endocytic trafficking. Nat Cell Biol. 2008;10:776. 18552835 10.1038/ncb1740 PMC2878716

[76] Neufeld EB , O'Brien K , Walts AD , et al. Cellular localization and trafficking of the human ABCG1 transporter. Biology (Basel). 2014;3:781‐800. 25405320 10.3390/biology3040781 PMC4280511

[77] Vázquez CL , Colombo MI . Assays to assess autophagy induction and fusion of autophagic vacuoles with a degradative compartment, using monodansylcadaverine (MDC) and DQ‐BSA. Methods Enzymol. 2009;452:85‐95. 19200877 10.1016/S0076-6879(08)03606-9

[78] Siddharth S , Muniraj N , Saxena NK , Sharma D . Concomitant inhibition of Cytoprotective autophagy augments the efficacy of Withaferin A in hepatocellular carcinoma. Cancers (Basel). 2019;11:453. 10.3390/cancers11040453 PMC6521104 30934990

[79] Muniraj N , Siddharth S , Shriver M , et al. Induction of STK11‐dependent cytoprotective autophagy in breast cancer cells upon honokiol treatment. Cell Death Discov. 2020;6:81. 32963809 10.1038/s41420-020-00315-w PMC7475061

[80] Bonam SR , Wang F , Muller S . Lysosomes as a therapeutic target. Nat Rev Drug Discov. 2019;18:923‐948. 31477883 10.1038/s41573-019-0036-1 PMC7097195

[81] Kolter T , Sandhoff K . Lysosomal degradation of membrane lipids. FEBS Lett. 2010;584:1700‐1712. 19836391 10.1016/j.febslet.2009.10.021

[82] Bissig C , Hurbain I , Raposo G , van Niel G . PIKfyve activity regulates reformation of terminal storage lysosomes from endolysosomes. Traffic. 2017;18:747‐757. 28857423 10.1111/tra.12525

[83] Vogler C , Rosenberg HS , Williams JC , Butler I . Electron microscopy in the diagnosis of lysosomal storage diseases. Am J Med Genet Suppl. 1987;3:243‐255. 2835968 10.1002/ajmg.1320280529

[84] Klumperman J , Raposo G . The complex ultrastructure of the endolysosomal system. Cold Spring Harb Perspect Biol. 2014;6:a016857. 24851870 10.1101/cshperspect.a016857 PMC4176003

[85] Hess MW , Huber LA . Measuring lysosomal size and frequency by electron microscopy. Methods Cell Biol. 2021;164:47‐61. 34225918 10.1016/bs.mcb.2020.10.019

[86] Bright NA , Wartosch L , Luzio JP . Lysosome fusion in cultured mammalian cells. Methods Cell Biol. 2015;126:101‐118. 25665443 10.1016/bs.mcb.2014.10.029

[87] Kilpatrick BS , Eden ER , Hockey LN , Futter CE , Patel S . Methods for monitoring lysosomal morphology. Methods Cell Biol. 2015;126:1‐19. 25665438 10.1016/bs.mcb.2014.10.018

[88] Polishchuk EV , Polishchuk RS . Pre‐embedding labeling for subcellular detection of molecules with electron microscopy. Tissue Cell. 2019;57:103‐110. 30497685 10.1016/j.tice.2018.11.002

[89] Melo RCN , Morgan E , Monahan‐Earley R , Dvorak AM , Weller PF . Pre‐embedding immunogold labeling to optimize protein localization at subcellular compartments and membrane microdomains of leukocytes. Nat Protoc. 2014;9:2382. 25211515 10.1038/nprot.2014.163 PMC4204927

[90] Tao‐Cheng JH , Crocker V , Moreira SL , Azzam R . Optimization of protocols for pre‐embedding immunogold electron microscopy of neurons in cell cultures and brains. Mol Brain. 2021;14:1‐15. 34082785 10.1186/s13041-021-00799-2 PMC8173732

[91] Möbius W , Posthuma G . Sugar and ice: Immunoelectron microscopy using cryosections according to the Tokuyasu method. Tissue Cell. 2019;57:90‐102. 30201442 10.1016/j.tice.2018.08.010

[92] Kubota Y . New developments in electron microscopy for serial image acquisition of neuronal profiles. Microscopy. 2015;64:27‐36. 25564566 10.1093/jmicro/dfu111

[93] Peddie CJ , Collinson LM . Exploring the third dimension: volume electron microscopy comes of age. Micron. 2014;61:9‐19. 24792442 10.1016/j.micron.2014.01.009

[94] Fermie J , Liv N , Ten Brink C , et al. Single organelle dynamics linked to 3D structure by correlative live‐cell imaging and 3D electron microscopy. Traffic. 2018;19:354‐369. 29451726 10.1111/tra.12557

[95] van der Beek J , de Heus C , Liv N , Klumperman J . Quantitative correlative microscopy reveals the ultrastructural distribution of endogenous endosomal proteins. J Cell Biol. 2022;221:e202106044. 34817533 10.1083/jcb.202106044 PMC8624803

[96] Sedzicki J , Tschon T , Low SH , et al. 3D correlative electron microscopy reveals continuity of Brucellacontaining vacuoles with the endoplasmic reticulum. J Cell Sci. 2018;131:jcs210799. 29361547 10.1242/jcs.210799

[97] Miller K , Beardmore J , Kanety H , Schlessinger J , Hopkins CR . Localization of the epidermal growth factor (EGF) receptor within the endosome of EGF‐stimulated epidermoid carcinoma (A431) cells. J Cell Biol. 1986;102:500‐509. 2868013 10.1083/jcb.102.2.500 PMC2114073

[98] Bright NA , Reaves BJ , Mullock BM , Luzio JP . Dense core lysosomes can fuse with late endosomes and are re‐formed from the resultant hybrid organelles. J Cell Sci. 1997;110:2027‐2040. 9378754 10.1242/jcs.110.17.2027

[99] Faulk WP , Taylor GM . An immunocolloid method for the electron microscope. Immunochemistry. 1971;8:1081‐1083. 4110101 10.1016/0019-2791(71)90496-4

[100] Hisano S , Adachi T , Daikoku S . Immunolabeling of adenohypophysial cells with protein A‐colloidal gold–antibody complex for electron microscopy: use of the freeze‐substitution technique in tissue preparation. J Histochem Cytochem. 1984;32:705‐711. 6736623 10.1177/32.7.6736623

[101] Norris RP , Terasaki M . Gap junction internalization and processing in vivo: a 3D immuno‐electron microscopy study. J Cell Sci. 2021;134:jcs252726. 33277382 10.1242/jcs.252726

[102] Slot JW , Geuze HJ . Cryosectioning and immunolabeling. Nat Protoc. 2007;2:2480‐2491. 17947990 10.1038/nprot.2007.365

[103] Anderson RG , Falck JR , Goldstein JL , Brown MS . Visualization of acidic organelles in intact cells by electron microscopy. Proc Natl Acad Sci U S A. 1984;81:4838‐4842. 6146980 10.1073/pnas.81.15.4838 PMC391586

[104] Alpy F , Rousseau A , Schwab Y , et al. STARD3 or STARD3NL and VAP form a novel molecular tether between late endosomes and the ER. J Cell Sci. 2013;126:5500‐5512. 24105263 10.1242/jcs.139295

[105] Vihinen H , Belevich I , Jokitalo E . Three dimensional electron microscopy of cellular organelles by serial block face SEM and ET. Microsc Anal. 2013;27:4.

[106] Bushby AJ , P'Ng KM , Young RD , Pinali C , Knupp C , Quantock AJ . Imaging three‐dimensional tissue architectures by focused ion beam scanning electron microscopy. Nat Protoc. 2011;6:845‐858. 21637203 10.1038/nprot.2011.332

[107] Herzik MA Jr . Cryo‐electron microscopy reaches atomic resolution. Nature. 2020;587:39‐40. 33087866 10.1038/d41586-020-02924-y

[108] Hoffman DP , Shtengel G , Xu CS , et al. Correlative three‐dimensional super‐resolution and block‐face electron microscopy of whole vitreously frozen cells. Science. 2020;367:eaaz5357. 31949053 10.1126/science.aaz5357 PMC7339343

[109] Daniele T , Schiaffino MV . Organelle biogenesis and interorganellar connections: better in contact than in isolation. Commun Integr Biol. 2014;7:e29587. 25346798 10.4161/cib.29587 PMC4203768

[110] Wu H , Carvalho P , Voeltz GK . Here, there, and everywhere: the importance of ER membrane contact sites. Science. 2018;361:eaan5835. 30072511 10.1126/science.aan5835 PMC6568312

[111] Jongsma ML , Berlin I , Wijdeven RH , et al. An ER‐associated pathway defines endosomal architecture for controlled cargo transport. Cell. 2016;166:152‐166. 27368102 10.1016/j.cell.2016.05.078 PMC4930482

[112] Rocha N , Kuijl C , van der Kant R , et al. Cholesterol sensor ORP1L contacts the ER protein VAP to control Rab7‐RILP‐p150 glued and late endosome positioning. J Cell Biol. 2009;185:1209‐1225. 19564404 10.1083/jcb.200811005 PMC2712958

[113] Atakpa P , Thillaiappan NB , Mataragka S , Prole DL , Taylor CW . IP3 receptors preferentially associate with ER‐lysosome contact sites and selectively deliver Ca2+ to lysosomes. Cell Rep. 2018;25:3180‐3193. 30540949 10.1016/j.celrep.2018.11.064 PMC6302550

[114] Morgan AJ , Davis LC , Wagner SK , et al. Bidirectional Ca(2)(+) signaling occurs between the endoplasmic reticulum and acidic organelles. J Cell Biol. 2013;200:789‐805. 23479744 10.1083/jcb.201204078 PMC3601362

[115] Allison R , Edgar JR , Pearson G , et al. Defects in ER‐endosome contacts impact lysosome function in hereditary spastic paraplegia. J Cell Biol. 2017;216:1337‐1355. 28389476 10.1083/jcb.201609033 PMC5412567

[116] Rowland AA , Chitwood PJ , Phillips MJ , Voeltz GK . ER contact sites define the position and timing of endosome fission. Cell. 2014;159:1027‐1041. 25416943 10.1016/j.cell.2014.10.023 PMC4634643

[117] Eden ER , White IJ , Tsapara A , Futter CE . Membrane contacts between endosomes and ER provide sites for PTP1B‐epidermal growth factor receptor interaction. Nat Cell Biol. 2010;12:267‐272. 20118922 10.1038/ncb2026

[118] Palande K , Roovers O , Gits J , et al. Peroxiredoxin‐controlled G‐CSF signalling at the endoplasmic reticulum‐early endosome interface. J Cell Sci. 2011;124:3695‐3705. 22045733 10.1242/jcs.089656 PMC3215578

[119] Eden ER , Sanchez‐Heras E , Tsapara A , Sobota A , Levine TP , Futter CE . Annexin A1 tethers membrane contact sites that mediate ER to endosome cholesterol transport. Dev Cell. 2016;37:473‐483. 27270042 10.1016/j.devcel.2016.05.005 PMC4906250

[120] Zhao K , Ridgway ND . Oxysterol‐binding protein‐related protein 1L regulates cholesterol egress from the endo‐lysosomal system. Cell Rep. 2017;19:1807‐1818. 28564600 10.1016/j.celrep.2017.05.028

[121] Kilpatrick BS , Eden ER , Schapira AH , Futter CE , Patel S . Direct mobilisation of lysosomal Ca2+ triggers complex Ca2+ signals. J Cell Sci. 2013;126:60‐66. 23108667 10.1242/jcs.118836 PMC4208295

[122] Friedman JR , Dibenedetto JR , West M , Rowland AA , Voeltz GK . Endoplasmic reticulum‐endosome contact increases as endosomes traffic and mature. Mol Biol Cell. 2013;24:1030‐1040. 23389631 10.1091/mbc.E12-10-0733 PMC3608491

[123] Sheftel AD , Zhang AS , Brown C , Shirihai OS , Ponka P . Direct interorganellar transfer of iron from endosome to mitochondrion. Blood. 2007;110:125‐132. 17376890 10.1182/blood-2007-01-068148

[124] Brahimi‐Horn MC , Lacas‐Gervais S , Adaixo R , et al. Local mitochondrial‐endolysosomal microfusion cleaves voltage‐dependent anion channel 1 to promote survival in hypoxia. Mol Cell Biol. 2015;35:1491‐1505. 25691661 10.1128/MCB.01402-14 PMC4387213

[125] Peng W , Wong YC , Krainc D . Mitochondria‐lysosome contacts regulate mitochondrial ca(2+) dynamics via lysosomal TRPML1. Proc Natl Acad Sci U S A. 2020;117:19266‐19275. 32703809 10.1073/pnas.2003236117 PMC7430993

[126] Munoz‐Braceras S , Tornero‐Ecija AR , Vincent O , Escalante R . VPS13A is closely associated with mitochondria and is required for efficient lysosomal degradation. Dis Model Mech. 2019;12:dmm036681. 30709847 10.1242/dmm.036681 PMC6398486

[127] Valm AM , Cohen S , Legant WR , et al. Applying systems‐level spectral imaging and analysis to reveal the organelle interactome. Nature. 2017;546:162‐167. 28538724 10.1038/nature22369 PMC5536967

[128] Lu M , van Tartwijk FW , Lin JQ , et al. The structure and global distribution of the endoplasmic reticulum network are actively regulated by lysosomes. Sci Adv. 2020;6:eabc7209. 33328230 10.1126/sciadv.abc7209 PMC7744115

[129] Chen Q , Fang H , Shao X , et al. A dual‐labeling probe to track functional mitochondria‐lysosome interactions in live cells. Nat Commun. 2020;11:6290. 33293545 10.1038/s41467-020-20067-6 PMC7722883

[130] Han Y , Li M , Qiu F , Zhang M , Zhang YH . Cell‐permeable organic fluorescent probes for live‐cell long‐term super‐resolution imaging reveal lysosome‐mitochondrion interactions. Nat Commun. 2017;8:1307. 29101340 10.1038/s41467-017-01503-6 PMC5670236

[131] Cabantous S , Terwilliger TC , Waldo GS . Protein tagging and detection with engineered self‐assembling fragments of green fluorescent protein. Nat Biotechnol. 2005;23:102‐107. 15580262 10.1038/nbt1044

[132] Magliery TJ , Wilson CG , Pan W , et al. Detecting protein‐protein interactions with a green fluorescent protein fragment reassembly trap: scope and mechanism. J Am Chem Soc. 2005;127:146‐157. 15631464 10.1021/ja046699g

[133] Alford SC , Ding Y , Simmen T , Campbell RE . Dimerization‐dependent green and yellow fluorescent proteins. ACS Synth Biol. 2012;1:569‐575. 23656278 10.1021/sb300050j PMC3653836

[134] Alford SC , Abdelfattah AS , Ding Y , Campbell RE . A fluorogenic red fluorescent protein heterodimer. Chem Biol. 2012;19:353‐360. 22444590 10.1016/j.chembiol.2012.01.006 PMC3560288

[135] Petkovic M , Oses‐Prieto J , Burlingame A , Jan LY , Jan YN . TMEM16K is an interorganelle regulator of endosomal sorting. Nat Commun. 2020;11:1‐16. 32620747 10.1038/s41467-020-17016-8 PMC7335067

[136] Özkan N , Koppers M , van Soest I , et al. ER – lysosome contacts at a pre‐axonal region regulate axonal lysosome availability. Nat Commun. 2021;12:1‐18. 34301956 10.1038/s41467-021-24713-5 PMC8302662

[137] Mitchell AC , Alford SC , Hunter SA , et al. Development of a protease biosensor based on a dimerization‐dependent red fluorescent protein. ACS Chem Biol. 2018;13:66‐72. 29125730 10.1021/acschembio.7b00715 PMC6453536

[138] Cieri D , Vicario M , Giacomello M , et al. SPLICS: a split green fluorescent protein‐based contact site sensor for narrow and wide heterotypic organelle juxtaposition. Cell Death Differ. 2018;25:1131‐1145. 29229997 10.1038/s41418-017-0033-z PMC5988678

[139] Forgac M . Vacuolar ATPases: rotary proton pumps in physiology and pathophysiology. Nat Rev Mol Cell Biol. 2007;8:917‐929. 17912264 10.1038/nrm2272

[140] Richter KN , Revelo NH , Seitz KJ , et al. Glyoxal as an alternative fixative to formaldehyde in immunostaining and super‐resolution microscopy. EMBO J. 2018;37:139‐159. 29146773 10.15252/embj.201695709 PMC5753035

[141] Stagi M , Klein ZA , Gould TJ , Bewersdorf J , Strittmatter SM . Lysosome size, motility and stress response regulated by fronto‐temporal dementia modifier TMEM106B. Mol Cell Neurosci. 2014;61:226‐240. 25066864 10.1016/j.mcn.2014.07.006 PMC4145808

[142] DiCiccio JE , Steinberg BE . Lysosomal pH and analysis of the counter ion pathways that support acidification. J Gen Physiol. 2011;137:385‐390. 21402887 10.1085/jgp.201110596 PMC3068279

[143] Diwu Z , Chen CS , Zhang C , Klaubert DH , Haugland RP . A novel acidotropic pH indicator and its potential application in labeling acidic organelles of live cells. Chem Biol. 1999;6:411‐418. 10381401 10.1016/s1074-5521(99)80059-3

[144] Ma L , Ouyang Q , Werthmann GC , Thompson HM , Morrow EM . Live‐cell microscopy and fluorescence‐based measurement of luminal pH in intracellular organelles. Front Cell Dev Biol. 2017;5:71. 28871281 10.3389/fcell.2017.00071 PMC5566985

[145] Guha S , Coffey EE , Lu W , et al. Approaches for detecting lysosomal alkalinization and impaired degradation in fresh and cultured RPE cells: evidence for a role in retinal degenerations. Exp Eye Res. 2014;126:68‐76. 25152362 10.1016/j.exer.2014.05.013 PMC4143779

[146] Domingues N , Estronca LMBB , Silva J , et al. Cholesteryl hemiesters alter lysosome structure and function and induce proinflammatory cytokine production in macrophages. Biochim Biophys Acta Mol Cell Biol Lipids. 1862;2017:210‐220. 10.1016/j.bbalip.2016.10.009 27793708

[147] Zhang Y , Zhao Y , Wu Y , Zhao B , Wang L , Song B . Hemicyanine based naked‐eye ratiometric fluorescent probe for monitoring lysosomal pH and its application. Spectrochim Acta A Mol Biomol Spectrosc. 2020;227:117767. 31707017 10.1016/j.saa.2019.117767

[148] Liu X , Su Y , Tian H , et al. Ratiometric fluorescent probe for lysosomal pH measurement and imaging in living cells using single‐wavelength excitation. Anal Chem. 2017;89:7038‐7045. 28553716 10.1021/acs.analchem.7b00754

[149] Ponsford AH , Ryan TA , Raimondi A , et al. Live imaging of intra‐lysosome pH in cell lines and primary neuronal culture using a novel genetically encoded biosensor. Autophagy. 2021;17:1500‐1518. 32515674 10.1080/15548627.2020.1771858 PMC8205096

[150] Webb BA , Aloisio FM , Charafeddine RA , Cook J , Wittmann T , Barber DL . pHLARE: A new biosensor reveals decreased lysosome pH in cancer cells. Mol Biol Cell. 2021;32:131‐142. 33237838 10.1091/mbc.E20-06-0383 PMC8120692

[151] Chin MY , Patwardhan AR , Ang KH , et al. Genetically encoded, pH‐sensitive mTFP1 biosensor for probing lysosomal pH. ACS Sens. 2021;6:2168‐2180. 34102054 10.1021/acssensors.0c02318 PMC8240087

[152] Morgan AJ , Platt FM , Lloyd‐Evans E , Galione A . Molecular mechanisms of endolysosomal Ca2+ signalling in health and disease. Biochem J. 2011;439:349‐378. 21992097 10.1042/BJ20110949

[153] Morgan AJ , Davis LC , Galione A . Imaging approaches to measuring lysosomal calcium. Methods Cell Biol. 2015;126:159‐195. 25665446 10.1016/bs.mcb.2014.10.031

[154] Gerasimenko JV , Tepikin AV , Petersen OH , Gerasimenko OV . Calcium uptake via endocytosis with rapid release from acidifying endosomes. Curr Biol. 1998;8:1335‐1338. 9843688 10.1016/s0960-9822(07)00565-9

[155] Calcraft PJ , Ruas M , Pan Z , et al. NAADP mobilizes calcium from acidic organelles through two‐pore channels. Nature. 2009;459:596‐600. 19387438 10.1038/nature08030 PMC2761823

[156] Yang J , Zhao Z , Gu M , Feng X , Xu H . Release and uptake mechanisms of vesicular Ca 2+ stores. Protein Cell. 2019;10:8‐19. 29549599 10.1007/s13238-018-0523-x PMC6321814

[157] Gerndt S , Chen CC , Chao YK , et al. Agonist‐mediated switching of ion selectivity in TPC2 differentially promotes lysosomal function. Elife. 2020;9:e54712. 32167471 10.7554/eLife.54712 PMC7108868

[158] Ronco V , Potenza DM , Denti F , et al. A novel Ca2+‐mediated cross‐talk between endoplasmic reticulum and acidic organelles: implications for NAADP‐dependent Ca2+ signalling. Cell Calcium. 2015;57:89‐100. 25655285 10.1016/j.ceca.2015.01.001

[159] Davis LC , Morgan AJ , Galione A . NAADP‐regulated two‐pore channels drive phagocytosis through endo‐lysosomal Ca 2+ nanodomains, calcineurin and dynamin. EMBO J. 2020;39:1‐23. 10.15252/embj.2019104058 PMC7360967 32510172

[160] Atakpa P , van Marrewijk LM , Apta‐Smith M , Chakraborty S , Taylor CW . GPN does not release lysosomal Ca 2+ but evokes Ca 2+ release from the ER by increasing the cytosolic pH independently of cathepsin C. J Cell Sci. 2019;132:jcs223883. 30617110 10.1242/jcs.223883 PMC6382017

[161] Berg TO , Strømhaug E , Løvdal T , Seglen O , Berg T . Use of glycyl‐l‐phenylalanine 2‐naphthylamide, a lysosome‐disrupting cathepsin C substrate, to distinguish between lysosomes and prelysosomal endocytic vacuoles. Biochem J. 1994;300:229‐236. 8198538 10.1042/bj3000229 PMC1138146

[162] Yuan Y , Kilpatrick BS , Gerndt S , et al. The lysosomotrope GPN mobilises Ca2+ from acidic organelles. J Cell Sci. 2021;134:jcs256578. 33602742 10.1242/jcs.256578 PMC7972315

[163] Gunaratne GS , Brailoiu E , He S , et al. Essential requirement for JPT2 in NAADP‐evoked Ca 2+ signaling. Sci Signal. 2021;14:eabd5605. 33758061 10.1126/scisignal.abd5605 PMC8315109

[164] Parkesh R , Lewis AM , Aley PK , et al. Cell‐permeant NAADP: A novel chemical tool enabling the study of Ca2+ signalling in intact cells. Cell Calcium. 2008;43:531‐538. 17935780 10.1016/j.ceca.2007.08.006

[165] Schmiege P , Fine M , Blobel G , Li X . Human TRPML1 channel structures in open and closed conformations. Nature. 2017;550:366–370. 29019983 10.1038/nature24036 PMC5920536

[166] Kilpatrick BS , Eden ER , Hockey LN , Yates E , Futter CE , Patel S . An endosomal NAADP‐sensitive two‐pore Ca2+ channel regulates ER‐endosome membrane contact sites to control growth factor Signaling. Cell Rep. 2017;18:1636‐1645. 28199837 10.1016/j.celrep.2017.01.052 PMC5318655

[167] Garrity AG , Wang W , Collier CM , Levey SA , Gao Q , Xu H . The endoplasmic reticulum, not the pH gradient, drives calcium refilling of lysosomes. Elife. 2016;5:e15887. 27213518 10.7554/eLife.15887 PMC4909396

[168] Höglinger D , Haberkant P , Aguilera‐Romero A , et al. Intracellular sphingosine releases calcium from lysosomes. Elife. 2015;4:e10616. 26613410 10.7554/eLife.10616 PMC4744193

[169] McCue HV , Wardyn JD , Burgoyne RD , Haynes LP . Generation and characterization of a lysosomally targeted, genetically encoded Ca(2+)‐sensor. Biochem J. 2013;449:449‐457. 23098255 10.1042/BJ20120898 PMC3526116

[170] Nagai T , Yamada S , Tominaga T , Ichikawa M , Miyawaki A . Expanded dynamic range of fluorescent indicators for Ca(2+) by circularly permuted yellow fluorescent proteins. Proc Natl Acad Sci U S A. 2004;101:10554‐10559. 15247428 10.1073/pnas.0400417101 PMC490022

[171] Shen D , Wang X , Li X , et al. Lipid storage disorders block lysosomal trafficking by inhibiting a TRP channel and lysosomal calcium release. Nat Commun. 2012;3:731. 22415822 10.1038/ncomms1735 PMC3347486

[172] Tian L , Hires SA , Mao T , et al. Imaging neural activity in worms, flies and mice with improved GCaMP calcium indicators. Nat Methods. 2009;6:875‐881. 19898485 10.1038/nmeth.1398 PMC2858873

[173] Christensen KA , Myers JTSJA . pH‐dependent regulation of lysosomal calcium in macrophages. J Cell Sci. 2002;115:599‐607. 11861766 10.1242/jcs.115.3.599

[174] Kendall JM , Sala‐Newby G , Ghalaut V , Dormer RL , Campbell AK . Engineering the CA(2+)‐activated photoprotein aequorin with reduced affinity for calcium. Biochem Biophys Res Commun. 1992;187:1091‐1097. 1530606 10.1016/0006-291x(92)91309-e

[175] Lloyd‐Evans E , Morgan AJ , He X , et al. Niemann‐Pick disease type C1 is a sphingosine storage disease that causes deregulation of lysosomal calcium. Nat Med. 2008;14:1247‐1255. 18953351 10.1038/nm.1876

[176] Narayanaswamy N , Chakraborty K , Saminathan A , et al. A pH‐correctable, DNA‐based fluorescent reporter for organellar calcium. Nat Methods. 2019;16:95. 30532082 10.1038/s41592-018-0232-7 PMC7107693

[177] Saminathan A , Noyola VS , Krishnan Y . Chemically resolving lysosome populations in live cells. Trends Biochem Sci. 2020;45:365‐366. 32169175 10.1016/j.tibs.2019.12.006

[178] Duman JG , Chen L , Palmer AE , Hille B . Contributions of intracellular compartments to calcium dynamics: implicating an acidic store. Traffic. 2006;7:859‐872. 16787398 10.1111/j.1600-0854.2006.00432.x

[179] Albrecht T , Zhao Y , Nguyen TH , Campbell RE , Johnson JD . Fluorescent biosensors illuminate calcium levels within defined beta‐cell endosome subpopulations. Cell Calcium. 2015;57:263‐274. 25682167 10.1016/j.ceca.2015.01.008

[180] Luzio JP , Poupon V , Lindsay MR , Mullock BM , Piper RC , Pryor PR . Membrane dynamics and the biogenesis of lysosomes. Mol Membr Biol. 2003;20:141‐154. 12851071 10.1080/0968768031000089546

[181] Griffiths G , Hoflack B , Simons K , Mellman I , Kornfeld S . The mannose 6‐phosphate receptor and the biogenesis of lysosomes. Cell. 1988;52:329‐341. 2964276 10.1016/s0092-8674(88)80026-6

[182] Settembre C , Di MC , Polito VA , et al. TFEB links autophagy to lysosomal biogenesis. Science. 2011;332:1429. 21617040 10.1126/science.1204592 PMC3638014

[183] Bajaj L , Lotfi P , Pal R , di Ronza A , Sharma J , Sardiello DRM . Lysosome biogenesis in health and disease. J Neurochem. 2019;148:573. 30092616 10.1111/jnc.14564 PMC6368902

[184] Sardiello M , Palmieri M , di Ronza A , et al. A gene network regulating lysosomal biogenesis and function. Science. 2009;325:473‐477. 19556463 10.1126/science.1174447

[185] Palmieri M , Impey S , Kang H , et al. Characterization of the CLEAR network reveals an integrated control of cellular clearance pathways. Hum Mol Genet. 2011;20:3852‐3866. 21752829 10.1093/hmg/ddr306

[186] Chauhan S , Goodwin JG , Chauhan S , et al. ZKSCAN3 is a master transcriptional repressor of autophagy. Mol Cell. 2013;50:16‐28. 23434374 10.1016/j.molcel.2013.01.024 PMC3628091

[187] Gambardella G , Staiano L , Moretti MN , et al. GADD34 is a modulator of autophagy during starvation. Sci Adv. 2020;6:eabb0205. 32978159 10.1126/sciadv.abb0205 PMC7518873

[188] Napolitano G , Ballabio A . TFEB at a glance. J Cell Sci. 2016;129:2475‐2481. 27252382 10.1242/jcs.146365 PMC4958300

[189] Di Malta C , Siciliano D , Calcagni A , et al. Transcriptional activation of RagD GTPase controls mTORC1 and promotes cancer growth. Science. 2017;356:1188‐1193. 28619945 10.1126/science.aag2553 PMC5730647

[190] Settembre C , Zoncu R , Medina DL , et al. A lysosome‐to‐nucleus signalling mechanism senses and regulates the lysosome via mTOR and TFEB. EMBO J. 2012;31:1095‐1108. 22343943 10.1038/emboj.2012.32 PMC3298007

[191] Martina JA , Chen Y , Gucek M , Puertollano R . MTORC1 functions as a transcriptional regulator of autophagy by preventing nuclear transport of TFEB. Autophagy. 2012;8:903‐914. 22576015 10.4161/auto.19653 PMC3427256

[192] Roczniak‐Ferguson A , Petit CS , Froehlich F , et al. The transcription factor TFEB links mTORC1 signaling to transcriptional control of lysosome homeostasis. Sci Signal. 2012;5:228. 10.1126/scisignal.2002790 PMC3437338 22692423

[193] Napolitano G , Di Malta C , Esposito A , de Araujo MEG , Pece S , Bertalot G , Matarese M , Benedetti V , Zampelli A , Stasyk T , Siciliano D , Venuta A , Cesana M , Vilardo C , Nusco E , Monfregola J , Calcagnì A , Di Fiore PP , Huber LA , Ballabio A . A substrate‐specific mTORC1 pathway underlies Birt–Hogg–Dubé syndrome. Nature. 2020;585:597‐602. 32612235 10.1038/s41586-020-2444-0 PMC7610377

[194] Sancak Y , Peterson TR , Shaul YD , et al. The rag GTPases bind raptor and mediate amino acid signaling to mTORC1. Science. 2008;320:1496‐1501. 18497260 10.1126/science.1157535 PMC2475333

[195] Sancak Y , Bar‐Peled L , Zoncu R , Markhard AL , Nada S , Sabatini DM . Ragulator‐rag complex targets mTORC1 to the lysosomal surface and is necessary for its activation by amino acids. Cell. 2010;141:290‐303. 20381137 10.1016/j.cell.2010.02.024 PMC3024592

[196] Perera RM , Stoykova S , Nicolay BN , et al. Transcriptional control of autophagy‐lysosome function drives pancreatic cancer metabolism. Nature. 2015;524:361‐365. 26168401 10.1038/nature14587 PMC5086585

[197] Napolitano G , Esposito A , Choi H , et al. mTOR‐dependent phosphorylation controls TFEB nuclear export. Nat Commun. 2018;9:1‐10. 30120233 10.1038/s41467-018-05862-6 PMC6098152

[198] Medina DL , Di Paola S , Peluso I , et al. Lysosomal calcium signalling regulates autophagy through calcineurin and TFEB. Nat Cell Biol. 2015;17:288‐299. 25720963 10.1038/ncb3114 PMC4801004

[199] Martina JA , Diab HI , Lishu L , et al. The nutrient‐responsive transcription factor TFE3 promotes autophagy, lysosomal biogenesis, and clearance of cellular debris. Sci Signal. 2014;7:ra9. 24448649 10.1126/scisignal.2004754 PMC4696865

[200] Cabukusta B , Neefjes J . Mechanisms of lysosomal positioning and movement. Traffic. 2018;19:761‐769. 29900632 10.1111/tra.12587 PMC6175085

[201] Encarnação M , Espada L , Escrevente C , et al. A Rab3a‐dependent complex essential for lysosome positioning and plasma membrane repair. J Cell Biol. 2016;213:631‐640. 27325790 10.1083/jcb.201511093 PMC4915190

[202] Clark PJ , Evans FC . Distance to nearest neighbor as a measure of spatial relationships in populations. Ecology. 1954;35:445‐453.

[203] Schwenk BM , Lang CM , Hogl S , et al. The FTLD risk factor TMEM106B and MAP6 control dendritic trafficking of lysosomes. EMBO J. 2014;33:450‐467. 24357581 10.1002/embj.201385857 PMC3989627

[204] de Chaumont F , Dallongeville S , Chenouard N , et al. Icy: an open bioimage informatics platform for extended reproducible research. Nat Methods. 2012;97:690‐696. 10.1038/nmeth.2075 22743774

[205] Hilverling A , Szegö EM , Dinter E , Cozma D , Saridaki T , Falkenburger BH . Maturing autophagosomes are transported towards the cell periphery. Cell Mol Neurobiol. 2021;42:155‐171. 34106361 10.1007/s10571-021-01116-0 PMC8732932

[206] Filipek PA , de Araujo MEG , Vogel GF , et al. LAMTOR/Ragulator is a negative regulator of Arl8b‐ and BORC‐dependent late endosomal positioning. J Cell Biol. 2017;216:4199‐4215. 28993467 10.1083/jcb.201703061 PMC5716276

[207] Starling GP , Yip YY , Sanger A , Morton PE , Eden ER , Dodding MP . Folliculin directs the formation of a Rab34–RILP complex to control the nutrient‐dependent dynamic distribution of lysosomes. EMBO Rep. 2016;17:823‐841. 27113757 10.15252/embr.201541382 PMC4893818

[208] Hollenbeck PJ , Swanson JA . Radial extension of macrophage tubular lysosomes supported by kinesin. Nature. 1990;346:864‐866. 1697403 10.1038/346864a0

[209] Huynh KK , Eskelinen E‐L , Scott CC , Malevanets A , Saftig P , Grinstein S . LAMP proteins are required for fusion of lysosomes with phagosomes. EMBO J. 2007;26:313‐324. 17245426 10.1038/sj.emboj.7601511 PMC1783450

[210] Tinevez JY , Perry N , Schindelin J , et al. TrackMate: an open and extensible platform for single‐particle tracking. Methods. 2017;115:80‐90. 27713081 10.1016/j.ymeth.2016.09.016

[211] Schindelin J , Arganda‐Carreras I , Frise E , et al. Fiji: an open‐source platform for biological‐image analysis. Nat Methods. 2012;9:676‐682. 22743772 10.1038/nmeth.2019 PMC3855844

[212] Baas PW , Deitch JS , Black MM , Banker GA . Polarity orientation of microtubules in hippocampal neurons: uniformity in the axon and nonuniformity in the dendrite. Proc Natl Acad Sci. 1988;85:8335‐8339. 3054884 10.1073/pnas.85.21.8335 PMC282424

[213] Roney JC , Li S , Farfel‐Becker T , et al. Lipid‐mediated motor‐adaptor sequestration impairs axonal lysosome delivery leading to autophagic stress and dystrophy in Niemann‐pick type C. Dev Cell. 2021;56:1452‐1468. 33878344 10.1016/j.devcel.2021.03.032 PMC8137671

[214] Nirschl JJ , Magiera MM , Lazarus JE , Janke C , Holzbaur ELF . α‐Tubulin tyrosination and CLIP‐170 phosphorylation regulate the initiation of dynein‐driven transport in neurons. Cell Rep. 2016;14:2637‐2652. 26972003 10.1016/j.celrep.2016.02.046 PMC4819336

[215] Fellows AD , Rhymes ER , Gibbs KL , Greensmith L , Schiavo G . IGF1R regulates retrograde axonal transport of signalling endosomes in motor neurons. EMBO Rep. 2020;21:e49129. 32030864 10.15252/embr.201949129 PMC7054680

[216] Vukoja A , Rey U , Petzoldt AG , et al. Presynaptic biogenesis requires axonal transport of lysosome‐related vesicles. Neuron. 2018;99:1216‐1232.e7. 30174114 10.1016/j.neuron.2018.08.004

[217] Liao YC , Fernandopulle MS , Wang G , et al. RNA granules hitchhike on lysosomes for long‐distance transport, using Annexin A11 as a molecular tether. Cell. 2019;179:147‐164.e20. 31539493 10.1016/j.cell.2019.08.050 PMC6890474

[218] Gowrishankar S , Lyons L , Rafiq NM , Roczniak‐Ferguson A , De Camilli P , Ferguson SM . Overlapping roles of JIP3 and JIP4 in promoting axonal transport of lysosomes in human iPSC‐derived neurons. Mol Biol Cell. 2021;32:1094‐1103. 33788575 10.1091/mbc.E20-06-0382 PMC8351540

[219] Boecker CA , Olenick MA , Gallagher ER , Ward ME , Holzbaur ELF . ToolBox: live imaging of intracellular organelle transport in induced pluripotent stem cell‐derived neurons. Traffic. 2020;21:138‐155. 31603614 10.1111/tra.12701 PMC7061308

[220] Farfel‐Becker T , Roney JC , Cheng XT , Li S , Cuddy SR , Sheng ZH . Neuronal soma‐derived degradative lysosomes are continuously delivered to distal axons to maintain local degradation capacity. Cell Rep. 2019;28:51‐64.e4. 31269450 10.1016/j.celrep.2019.06.013 PMC6696943

[221] Lee S , Sato Y , Nixon RA . Lysosomal proteolysis inhibition selectively disrupts axonal transport of degradative organelles and causes an Alzheimer's‐like axonal dystrophy. J Neurosci. 2011;31:7817‐7830. 21613495 10.1523/JNEUROSCI.6412-10.2011 PMC3351137

[222] Mangeol P , Prevo B , Peterman EJG . KymographClear and KymographDirect: two tools for the automated quantitative analysis of molecular and cellular dynamics using kymographs. Mol Biol Cell. 2016;27:1948‐1957. 27099372 10.1091/mbc.E15-06-0404 PMC4907728

[223] Neumann S , Chassefeyre R , Campbell GE , Encalada SE . KymoAnalyzer: a software tool for the quantitative analysis of intracellular transport in neurons. Traffic. 2017;18:71‐88. 27770501 10.1111/tra.12456 PMC5473519

[224] Bier E . Drosophila, the golden bug, emerges as a tool for human genetics. Nat Rev Genet. 2005;6:9‐23. 15630418 10.1038/nrg1503

[225] Kimura S , Noda T , Yoshimori T . Dissection of the autophagosome maturation process by a novel reporter protein, tandem fluorescent‐tagged LC3. Autophagy. 2007;3:452‐460. 17534139 10.4161/auto.4451

[226] Lund VK , Lycas MD , Schack A , Andersen RC , Gether U , Kjaerulff O . Rab2 drives axonal transport of dense core vesicles and lysosomal organelles. Cell Rep. 2021;35:108973. 33852866 10.1016/j.celrep.2021.108973

[227] Fujita N , Huang W , Lin TH , et al. Genetic screen in drosophila muscle identifies autophagy‐mediated T‐tubule remodeling and a Rab2 role in autophagy. Elife. 2017;6:e23367. 28063257 10.7554/eLife.23367 PMC5249261

[228] Rosa‐Ferreira C , Sweeney ST , Munro S . The small G protein Arl8 contributes to lysosomal function and long‐range axonal transport in Drosophila. Biol Open. 2018;7:bio035964. 30115618 10.1242/bio.035964 PMC6176938

[229] del Rodríguez AV , Didiano D , Desplan C . Power tools for gene expression and clonal analysis in Drosophila. Nat Methods. 2012;9:47. 10.1038/nmeth.1800 PMC3574576 22205518

[230] Dunst S , Tomancak P . Imaging flies by fluorescence microscopy: principles, technologies, and applications. Genetics. 2019;211:15‐34. 30626639 10.1534/genetics.118.300227 PMC6325693

[231] Kakanj P , Eming SA , Partridge L , Leptin M . Long‐term in vivo imaging of Drosophila larvae. Nat Protoc. 2020;15:1158‐1187. 32042177 10.1038/s41596-019-0282-z

[232] Vagnoni A , Bullock SL . A simple method for imaging axonal transport in aging neurons using the adult Drosophila wing. Nat Protoc. 2016;11:1711‐1723. 27560175 10.1038/nprot.2016.112 PMC5027916

[233] Fang Y , Soares L , Bonini NM . Design and implementation of in vivo imaging of neural injury responses in the adult Drosophila wing. Nat Protoc. 2013;8:810‐819. 23589940 10.1038/nprot.2013.042 PMC4032490

[234] Guan W , Venkatasubramanian L , Baek M , Mann RS , Enriquez J . Visualize drosophila leg motor neuron axons through the adult cuticle. J Vis Exp. 2018;140:e58365. 10.3791/58365 PMC6544026 30451217

[235] Clark BS , Winter M , Cohen AR , Link BA . Generation of Rab‐based transgenic lines for in vivo studies of endosome biology in zebrafish. Dev Dyn. 2011;240:2452‐2465. 21976318 10.1002/dvdy.22758 PMC3197968

[236] Du Z , Song B , Zhang W , et al. Quantitative monitoring and visualization of hydrogen sulfide in vivo using a luminescent probe based on a ruthenium(II) complex. Angew Chem Int Ed. 2018;57:3999‐4004. 10.1002/anie.201800540 29393999

[237] Otis JP , Shen MC , Caldwell BA , Reyes Gaido OE , Farber SA . Dietary cholesterol and apolipoprotein A‐I are trafficked in endosomes and lysosomes in the live zebrafish intestine. Am J Physiol Gastrointest Liver Physiol. 2019;316:G350‐G365. 30629468 10.1152/ajpgi.00080.2018 PMC6415739

[238] Singh H , Sreedharan S , Tiwari K , et al. Two photon excitable graphene quantum dots for structured illumination microscopy and imaging applications: lysosome specificity and tissue‐dependent imaging. Chem Commun. 2019;55:521‐524. 10.1039/c8cc08610a 30556083

[239] Jadot M , Boonen M , Thirion J , et al. Accounting for protein subcellular localization: A compartmental map of the rat liver proteome. Mol Cell Proteomics. 2017;16:194. 27923875 10.1074/mcp.M116.064527 PMC5294208

[240] Della Valle MC , Sleat DE , Zheng H , Moore DF , Jadot M , Lobel P . Classification of subcellular location by comparative proteomic analysis of native and density‐shifted lysosomes. Mol Cell Proteomics. 2011;10:M110.006403. 10.1074/mcp.M110.006403 PMC3069348 21252268

[241] Tharkeshwar AK , Demedts D , Annaert W . Superparamagnetic nanoparticles for lysosome isolation to identify spatial alterations in lysosomal protein and lipid composition. STAR Protoc. 2020;1:100122. 33377016 10.1016/j.xpro.2020.100122 PMC7756972

[242] Abu‐Remaileh M , Wyant GA , Kim C , et al. Lysosomal metabolomics reveals V‐ATPase‐ and mTOR‐dependent regulation of amino acid efflux from lysosomes. Science. 2017;358:807‐813. 29074583 10.1126/science.aan6298 PMC5704967

[243] Chen WW , Freinkman E , Sabatini DM . Rapid immunopurification of mitochondria for metabolite profiling and absolute quantification of matrix metabolites. Nat Protoc. 2017;12:2215. 29532801 10.1038/nprot.2017.104 PMC5851482

[244] Cinque L , De Leonibus C , Iavazzo M , et al. MiT/TFE factors control ER‐phagy via transcriptional regulation of FAM134B. EMBO J. 2020;39:e105696. 32716134 10.15252/embj.2020105696 PMC7459426

[245] Zellner S , Nalbach K , Behrends C . Autophagosome content profiling using proximity biotinylation proteomics coupled to protease digestion in mammalian cells. STAR Protoc. 2021;2:100506. 33997820 10.1016/j.xpro.2021.100506 PMC8102175

[246] Ingemann L , Kirkegaard T . Lysosomal storage diseases and the heat shock response: convergences and therapeutic opportunities. J Lipid Res. 2014;55:2198‐2210. 24837749 10.1194/jlr.R048090 PMC4617124

[247] Aits S , Kricker J , Liu B , et al. Sensitive detection of lysosomal membrane permeabilization by lysosomal galectin puncta assay. Autophagy. 2015;11:1408‐1424. 26114578 10.1080/15548627.2015.1063871 PMC4590643

[248] Jia J , Claude‐Taupin A , Gu Y , et al. Galectin‐3 coordinates a cellular system for lysosomal repair and removal. Dev Cell. 2020;52:69‐87. 31813797 10.1016/j.devcel.2019.10.025 PMC6997950

[249] Radulovic M , Schink KO , Wenzel EM , et al. ESCRT‐mediated lysosome repair precedes lysophagy and promotes cell survival. EMBO J. 2018;37:e99753. 30314966 10.15252/embj.201899753 PMC6213280

[250] Saftig P , Puertollano R . How lysosomes sense, integrate, and cope with stress. Trends Biochem Sci. 2021;46:97‐112. 33012625 10.1016/j.tibs.2020.09.004 PMC7855039

[251] Cooper GM . The Cell: A Molecular Approach. 2nd ed. Sinauer Associates; 2000.

[252] Yu C , Sun Q , Zhou H . Enzymatic screening and diagnosis of lysosomal storage diseases. N Am J Med Sci. 2013;6:186. 10.7156/najms.2013.0604186 PMC4902264 27293520

[253] Chamoles NA , Blanco M , Gaggioli D . Diagnosis of α‐l‐iduronidase deficiency in dried blood spots on filter paper: the possibility of newborn diagnosis. Clin Chem. 2001;47:780‐781. 11274042

[254] Parenti G , Medina DL , Ballabio A . The rapidly evolving view of lysosomal storage diseases. EMBO Mol Med. 2021;13:e12836. 33459519 10.15252/emmm.202012836 PMC7863408

[255] Zhang XK , Elbin CS , Turecek F , et al. Multiplex lysosomal enzyme activity assay on dried blood spots using tandem mass spectrometry. Methods Mol Biol. 2010;603:339. 20077085 10.1007/978-1-60761-459-3_32 PMC3442156

[256] Li Y , Scott CR , Chamoles NA , et al. Direct multiplex assay of lysosomal enzymes in dried blood spots for newborn screening. Clin Chem. 2004;50:1785‐1796. 15292070 10.1373/clinchem.2004.035907 PMC3428798

[257] Verma J , Thomas DC , Kasper DC , et al. Inherited metabolic disorders: efficacy of enzyme assays on dried blood spots for the diagnosis of lysosomal storage disorders. JIMD Rep. 2017;31:15‐27. 27008195 10.1007/8904_2016_548 PMC5388645

[258] Aerts JMFG , Sa Miranda MC , Brouwer‐Kelder EM , Van Weely S , Barranger JA , Tager JM . Conditions affecting the activity of glucocerebrosidase purified fom spleens of control subjects and patients with type 1 Gaucher disease. Biochim Biophys Acta. 1990;1041:55‐63. 2223847 10.1016/0167-4838(90)90122-v

[259] Mokhtariye A , Hagh‐Nazari L , Varasteh AR , Keyfi F . Diagnostic methods for lysosomal storage disease. Rep Biochem Mol Biol. 2019;7:119‐128. 30805390 PMC6374068

[260] Briciu‐Burghina C , Heery B , Regan F . Continuous fluorometric method for measuring β‐glucuronidase activity: comparative analysis of three fluorogenic substrates. Analyst. 2015;140:5953‐5964. 26225370 10.1039/c5an01021g

[261] Smith EL , Bertozzi CR , Beatty KE . An expanded set of fluorogenic sulfatase activity probes. Chembiochem. 2014;15:1101‐1105. 24764280 10.1002/cbic.201400032 PMC4084507

[262] Motabar O , Goldin E , Leister W , et al. A high throughput glucocerebrosidase assay using the natural substrate glucosylceramide. Anal Bioanal Chem. 2012;402:731‐739. 22033823 10.1007/s00216-011-5496-z PMC3351006

[263] Löser R , Pietzsch J . Cysteine cathepsins: their role in tumor progression and recent trends in the development of imaging probes. Front Chem. 2015;3:37. 26157794 10.3389/fchem.2015.00037 PMC4477214

[264] Van Noorden CJF . Imaging enzymes at work: metabolic mapping by enzyme histochemistry. J Histochem Cytochem. 2010;58:481‐497. 20124092 10.1369/jhc.2010.955518 PMC2874181

[265] Cermak S , Kosicek M , Mladenovic‐Djordjevic A , Smiljanic K , Kanazir S , Hecimovic S . Loss of cathepsin B and L leads to lysosomal dysfunction, NPC‐like cholesterol sequestration and accumulation of the key Alzheimer's proteins. PLoS One. 2016;11:e0167428. 27902765 10.1371/journal.pone.0167428 PMC5130271

[266] Schröder SP , Van De Sande JW , Kallemeijn WW , et al. Towards broad spectrum activity‐based glycosidase probes: synthesis and evaluation of deoxygenated cyclophellitol aziridines. Chem Commun. 2017;53:12528‐12531. 10.1039/c7cc07730k 29116266

[267] Fang H , Peng B , Ong SY , Wu Q , Li L , Yao SQ . Recent advances in activity‐based probes (ABPs) and affinity‐based probes (A f BPs) for profiling of enzymes. Chem Sci. 2021;12:8288‐8310. 34221311 10.1039/d1sc01359a PMC8221178

[268] Wu L , Armstrong Z , Schröder SP , et al. An overview of activity‐based probes for glycosidases. Curr Opin Chem Biol. 2019;53:25‐36. 31419756 10.1016/j.cbpa.2019.05.030

[269] van Meel E , Bos E , van der Lienden MJC , et al. Localization of active endogenous and exogenous β‐glucocerebrosidase by correlative light‐electron microscopy in human fibroblasts. Traffic. 2019;20:346‐356. 30895685 10.1111/tra.12641 PMC6519279

[270] Kuo CL , van Meel E , Kytidou K , et al. Activity‐based probes for glycosidases: profiling and other applications. Methods Enzymol. 2018;598:217‐235. 29306436 10.1016/bs.mie.2017.06.039

[271] Kallemeijn WW , Scheij S , Hoogendoorn S , et al. Investigations on therapeutic glucocerebrosidases through paired detection with fluorescent activity‐based probes. PLoS One. 2017;12:e0170268. 28207759 10.1371/journal.pone.0170268 PMC5313132

[272] Kallemeijn WW , Witte MD , Voorn‐Brouwer TM , et al. A sensitive gel‐based method combining distinct cyclophellitol‐based probes for the identification of acid/base residues in human retaining β‐glucosidases. J Biol Chem. 2014;289:35351‐35362. 25344605 10.1074/jbc.M114.593376 PMC4271221

[273] Kuo CL , van Meel E , Kytidou K , et al. Activity‐based probes for glycosidases: profiling and other applications. Methods in Enzymology. Academic Press Inc.; 2018:217‐235. 10.1016/bs.mie.2017.06.039 29306436

[274] Bellomo F , Medina DL , De Leo E , Panarella A , Emma F . High‐content drug screening for rare diseases. J Inherit Metab Dis. 2017;40:601‐607. 28593466 10.1007/s10545-017-0055-1

[275] Rodríguez A , Webster P , Ortego J , Andrews NW . Lysosomes behave as Ca2+‐regulated exocytic vesicles in fibroblasts and epithelial cells. J Cell Biol. 1997;137:93‐104. 9105039 10.1083/jcb.137.1.93 PMC2139854

[276] Jaiswal JK , Andrews NW , Simon SM . Membrane proximal lysosomes are the major vesicles responsible for calcium‐dependent exocytosis in nonsecretory cells. J Cell Biol. 2002;159:625‐635. 12438417 10.1083/jcb.200208154 PMC2173094

[277] Tancini B , Buratta S , Delo F , et al. Lysosomal exocytosis: the extracellular role of an intracellular organelle. Membranes (Basel). 2020;10:1‐21. 10.3390/membranes10120406 PMC7764620 33316913

[278] Escrevente C , Bento‐Lopes L , Ramalho JS , Barral DC . Rab11 is required for lysosome exocytosis through the interaction with Rab3a, Sec15 and GRAB. J Cell Sci. 2021;134:jcs246694. 34100549 10.1242/jcs.246694 PMC8214760

[279] Tsunemi T , Perez‐Rosello T , Ishiguro Y , et al. Increased lysosomal exocytosis induced by lysosomal Ca2+ channel agonists protects human dopaminergic neurons from α‐synuclein toxicity. J Neurosci. 2019;39:5760‐5772. 31097622 10.1523/JNEUROSCI.3085-18.2019 PMC6636071

[280] Steffan JJ , Snider JL , Skalli O , Welbourne T , Cardelli JA . Na+/H+ exchangers and RhoA regulate acidic extracellular pH‐induced lysosome trafficking in prostate cancer cells. Traffic. 2009;10:737‐753. 19302267 10.1111/j.1600-0854.2009.00904.x

[281] Damaghi M , Tafreshi NK , Lloyd MC , et al. Chronic acidosis in the tumour microenvironment selects for overexpression of LAMP2 in the plasma membrane. Nat Commun. 2015;6:8752. 26658462 10.1038/ncomms9752 PMC4682176

[282] Pu J , Guardia CM , Keren‐Kaplan T , Bonifacino JS . Mechanisms and functions of lysosome positioning. J Cell Sci. 2016;129:4329‐4339. 27799357 10.1242/jcs.196287 PMC5201012

[283] Samie MA , Xu H . Lysosomal exocytosis and lipid storage disorders. J Lipid Res. 2014;55:995‐1009. 24668941 10.1194/jlr.R046896 PMC4031951

[284] Andrews NW . Detection of lysosomal exocytosis by surface exposure of Lamp1 luminal epitopes. Methods in Molecular Biology. Humana Press Inc.; 2017:205‐211. 10.1007/978-1-4939-6934-0_13 28456985

[285] Reddy A , Caler EV , Andrews NW . Plasma membrane repair is mediated by Ca(2+)‐regulated exocytosis of lysosomes. Cell. 2001;106:157‐169. 11511344 10.1016/s0092-8674(01)00421-4

[286] Zhitomirsky B , Assaraf YG . Lysosomal accumulation of anticancer drugs triggers lysosomal exocytosis. Oncotarget. 2017;8:45117‐45132. 28187461 10.18632/oncotarget.15155 PMC5542171

[287] Ghosh S , Dellibovi‐Ragheb TA , Kerviel A , et al. β‐Coronaviruses use lysosomes for egress instead of the biosynthetic secretory pathway. Cell. 2020;183:1520‐1535. 33157038 10.1016/j.cell.2020.10.039 PMC7590812

[288] Medina DL , Fraldi A , Bouche V , et al. Transcriptional activation of lysosomal exocytosis promotes cellular clearance. Dev Cell. 2011;21:421‐430. 21889421 10.1016/j.devcel.2011.07.016 PMC3173716

[289] Castro‐Gomes T , Corrotte M , Tam C , Andrews NW . Plasma membrane repair is regulated extracellularly by proteases released from lysosomes. PLoS One. 2016;11:1‐26. 10.1371/journal.pone.0152583 PMC4814109 27028538

[290] Appelqvist H , Wäster P , Kågedal K , Öllinger K . The lysosome: from waste bag to potential therapeutic target. J Mol Cell Biol. 2013;5:214‐226. 23918283 10.1093/jmcb/mjt022

[291] Wang G , Nola S , Bovio S , et al. Biomechanical control of lysosomal secretion via the VAMP7 hub: a tug‐of‐war between VARP and LRRK1. iScience. 2018;4:127‐143. 30240735 10.1016/j.isci.2018.05.016 PMC6147023

[292] Cheng X , Zhang X , Gao Q , et al. The intracellular Ca2+ channel MCOLN1 is required for sarcolemma repair to prevent muscular dystrophy. Nat Med. 2014;20:1187‐1192. 25216637 10.1038/nm.3611 PMC4192061

[293] Polishchuk R , Di Pentima A , Lippincott‐Schwartz J . Delivery of raft‐associated, GPI‐anchored proteins to the apical surface of polarized MDCK cells by a transcytotic pathway. Nat Cell Biol. 2004;6:297‐307. 15048124 10.1038/ncb1109

[294] Newman TM , Tian M , Gomperts BD . Ultrastructural characterization of tannic acid‐arrested degranulation of permeabilized Guinea pig eosinophils stimulated with GTP‐γ‐S. Eur J Cell Biol. 1996;70:209‐220. 8832205

