# Supplementary material for: Current methods to analyze lysosome morphology, positioning, motility and function
Source: Traffic. 2022 Apr 24;23(5):238–69. doi: 10.1111/tra.12839 (PMC9323414; doi:10.1111/tra.12839)
Supplement: Supplementary file 1 — Data S1 Supporting Information. [file TRA-23-238-s001.docx]

**Supplementary Information**

**Table of Contents**

[**Supplementary Table 1** 3](#_Toc95421719)

[**Supplementary Table 2** 4](#_Toc95421720)

[**Supplementary Table 3** 5](#_Toc95421721)

[**Protocols** 12](#_Toc95421722)

[**1. High content imaging** 12](#_Toc95421723)

[**1.1. Dextran assay** 12](#_Toc95421724)

[**1.2. DQ-BSA assay** 12](#_Toc95421725)

[**1.3. GB3 assay** 13](#_Toc95421726)

[**1.4. Magic Red assay** 14](#_Toc95421727)

[**1.5. Lysosome membrane permeabilization assay** 14](#_Toc95421728)

[**2. Proximity ligation assay** 16](#_Toc95421729)

[**3. Immuno-EM: Pre-embedding labelling** 18](#_Toc95421730)

[**4. Loading lysosomes with BSA-gold for EM** 20](#_Toc95421731)

[**5. Ratiometric measurement of fluorescent conjugates of dextran to quantify lysosome luminal pH** 21](#_Toc95421732)

[**6. Monitoring of endo-lysosomal Ca^2+^ nanodomains** 24](#_Toc95421733)

[**7. Use of an LSD model (GLA-KO) to assess lysosome biogenesis** 30](#_Toc95421734)

[**8. Analysis of TFEB subcellular localization** 32](#_Toc95421735)

[**9. Lysosome immunopurification (LysoIP)** 34](#_Toc95421736)

[**10. Determination of cathepsin D activity in multiwell format using an artificial MCA-Dnp substrate** 37](#_Toc95421737)

[**11. *In situ* labelling with Magic Red^TM^ Cathepsin B/L kits** 39](#_Toc95421738)

[**12. LAMP1 cell surface detection by flow cytometry** 40](#_Toc95421739)

[**13. β-hexosaminidase release assay to assess lysosome exocytosis** 42](#_Toc95421740)

[**14. Measurement of β-hexosaminidase activity** 44](#_Toc95421741)

[**References** 45](#_Toc95421742)

**Supplementary Table 1** **– Cited antibodies used to study lysosomal morphology, function and signalling.**

| **Antigen** | **Host** | **Reactivity** | **Clonality** | **Application** | **Supplier** | **Cat. no.** |
| --- | --- | --- | --- | --- | --- | --- |
| LAMP1 | Mouse | Human | Monoclonal | IF | Hybridoma bank | H4A3 |
| LAMP1 | Rabbit | Human, Mouse | Polyclonal | IF, WB | Abcam | ab24170 |
| LAMP1 | Rat | Mouse | Monoclonal | IHC-P | Santa-Cruz | sc-19992 |
| CD63 | Mouse | Human | Monoclonal | IF | Hybridoma bank | H5C6 |
| Cation-independent MPR | Mouse | Human (predicted in mouse and rat) | Monoclonal | IF | Abcam | ab2733 |
| Cathepsin B | Rabbit | Mouse, Rat | Monoclonal | IF, IHC-P | Abcam | ab214428 |
| Cathepsin B | Goat | Human | Polyclonal | WB, IHC | R&D | AF953 |
| Cathepsin D | Rabbit | Mouse, Human | Monoclonal | IF, IHC-P | Abcam | ab75852 |
| Cathepsin D | Mouse | Human | Monoclonal | WB | Abcam | ab6313 |
| Cathepsin L | Goat | Human | Polyclonal | WB | R&D | AF952 |
| TFEB | Rabbit | Human | Polyclonal | IF, WB | Cell Signaling | 4240 |
| TFEB | Rabbit | Mouse | Polyclonal | IF, IHC-P, WB | Bethyl Laboratories | A303-673A |
| Phospho-TFEB (Ser211) | Rabbit | Human | Monoclonal | IF, WB | Cell Signaling | 37681 |
| Phospho-TFEB (Ser142) | Rabbit | Human | Monoclonal | WB | Millipore | ABE1971 |
| Phospho-p70 S6 Kinase (Thr389) (1A5) | Mouse | Mouse, Human | Monoclonal | WB | Cell Signaling | 9206 |
| Phospho-4E-BP1 (Ser65) (174A9) | Rabbit | Human | Monoclonal | WB | Cell Signaling | 9456 |
| mTOR (7C10) | Rabbit | Mouse, Human | Monoclonal | IF,WB | Cell Signaling | 2983 |
| Phospho-S6 ribosomal protein (Ser240/244) | Rabbit | Mouse, Human | Monoclonal | IF,WB, IHC-P | Cell Signaling | D68F8 |
| TFE3 | Rabbit | Mouse, Human | Polyclonal | IF,WB | Sigma | HPA023881 |
| Galectin-3 | Mouse | Mouse, Human | Monoclonal | IF,WB | Santa-Cruz | sc-23938 |
| Hemagglutinin | Rabbit |  | Polyclonal | IF, WB | Covance | PRB-101P |
| Acid α-glucosidase (GAA) | Rabbit | Mouse, Human | Polyclonal | WB | Abcam | Ab102815 |

IF - Immunofluorescence; WB - Western blot; IHC - Immunohistochemistry; IHC-P - Immunofluorescence on tissues

**Supplementary Table 2** **– Cited reagents and dyes used to study endolysosomal trafficking, lysosomal morphology, and function.**

| **Reagent** | **Supplier** | **Cat. no.** |
| --- | --- | --- |
| Bodipy FL-Pepstatin A | ThermoFisher | P12271 |
| Acridine Orange | ThermoFisher | A1301 |
| Dextran, Alexa Fluor 568 | ThermoFisher  ThermoFisher | D22912 |
| Dextran, Oregon Green 488 |  | D7173 |
| Dextran, Fluorescein | ThermoFisher | D1820 |
| Dextran, Lucifer Yellow | ThermoFisher | D1825 |
| pHrodo green dextran | ThermoFisher | P35368 |
| Cal-520-dextran conjugate | AAT Bioquest | 20601 |
| DQ Red BSA | ThermoFisher | D12051 |
| BSA, Alexa Fluor 488 | ThermoFisher | A13100 |
| Epidermal growth factor Alexa Fluor 555-conjugated | ThermoFisher | E35350 |
| LysoTracker Red DND-99 | ThermoFisher | L7528 |
| LysoTracker Deep Red | ThermoFisher | L12492 |
| LysoSensor Yellow/Blue DND-160 | ThermoFisher | L7545 |
| Cathepsin B assay kit (Magic Red) | Abcam | ab270772 |
| Fluorescein-5-isothiocyanate (FITC, pH-sensitive fluorophore) | Sigma | 3326-32-7 |
| pHrodo green AM intracellular pH indicator | ThermoFisher | P35373 |
| TopFluor-cholesterol | AvantiPolar Lipids | 810255 |
| Bafilomycin A1 (from *Streptomyces griseus*) | Sigma | B1793 |
| Gly-Phe β-naphthylamide (GPN) | Sigma | G9512 |
| 3,3′-Diaminobenzidine | Sigma | D12384 |
| Horseradish peroxidase (HRP) | Sigma | P8250 |
| LLOMe (L-Leucyl-L-Leucine methyl ester hydrochloride) | MedChemExpress | HY-129905 |

**Supplementary Table 3** – **Artificial substrates and probes for *in vitro* and *in vivo* monitoring of the activity of known lysosomal hydrolases**. For each enzyme the family, sub-family, Enzyme Commission (EC) number and chromosomal location (cytogenetics) are indicated.

| **Family** | **Sub-Family** | **Enzyme** | **Gene** | **EC Number** | **Family** | **Cytogenetics** | ***In vitro* substrates** | ***In vivo* substrates & ABPS** |
| --- | --- | --- | --- | --- | --- | --- | --- | --- |
| Glycosidase |  | α-galactosidase A | GLA | 3.2.1.22 | GH27 | Xq22.1 | X-α-D-galactopyranoside | [1] |
|  |  | α-L-fucosidase | FUCA1 | 3.2.1.51 | GH29 | 1-p36.11 | X-α-D-fucoside | [2] |
|  |  | α-L-iduronidase | IDUA | 3.2.1.76 | GH39 | 4p16.3 | X-α-L-iduronide | [3] |
|  |  | α-mannosidase | MAN2B1 | 3.2.1.24 | GH38 | 19p13.13 | X-α-D-mannopyranoside | [4] |
|  |  | Acid α-glucosidase | GAA | 3.2.1.20/3 | GH31 | 17q25.3 | X-α-D-glucopyranoside;  X-α-D-maltoheptaoside | [5] |
|  |  | α-N-acetyl-galactosaminidase | NAGA | 3.2.1.49 | GH27 | 22q13.2 | X-α-D-acetylgalactosamine;  X-α-galactose | [6] |
|  |  | N-acetyl-α-glucosaminidase | NAGLU | 3.2.1.50 | GH89 | 17q21.2 | X-2-acetamide-2-deoxy-α-D-glucopyranoside; X-N-acetyl-α-D-glucosaminide |  |
|  |  | β-galactosidase | GLB1 | 3.2.1.23 | GH35 | 3p22.3 | X-β-D-galactopyranoside | [7]  2',7'-dichlorofluorescein;  Di-ß-D-galactopyranoside |
|  |  | β-glucuronidase | GUSB | 3.2.1.31 | GH2 | 7q11.21 | X-β-D-glucuronide | [8] |
|  |  | β-hexosaminidase HexA | HEXA | 3.2.1.52 | GH20 | 15q23 | X-β-D-N-acetylglucosamine-6-sulfate |  |
|  |  | β-Hexosaminidases HexA/HexB | HEXB | 3.2.1.52 | GH20 | 5q13.3 | X-N-acetyl-β-D-glucosaminide | Fluorescein mono-β-D-N-acetylglucosamine [9] |
|  |  | β-mannosidase | MANBA | 3.2.1.25 | GH2 | 4q24 | X-β-D-mannopyranoside |  |
|  |  | Chitotriosidase1 | CHIT1 | 3.2.1.14 | GH18 | 1q32.1 | X-β-D-N,N′,N′′-triacetylchitotrioside |  |
|  |  | Di-N-acetylchitobiase | CTBS | 3.2.1.- | GH18 | 1p22.3 |  |  |
|  |  | Galactocerebrosidase | GALC | 3.2.1.46 | GH59 | 14q31.3 | X-β-D-galactopyranoside | [10]  2',7'-dichlorofluorescein; Di-ß-D-galactopyranoside |
|  |  | Glucosylceramidase (β-glucocerebrosidase) | GBA | 3.2.1.45 | GH30 | 1q22 | X-β-D-glucopyranoside | [11]  5-(Pentafluorobenzoylamino) Fluorescein Di-β-D-glucopyranoside [12] |
|  |  | Heparanase | HPSE | 3.2.1.166 | GH79 | 4q21.23 | HADP [13] |  |
|  |  | Hyaluronidase 1 | HYAL1 | 3.2.1.35 | GH56 | 3p21.31 | Hyaluronic acid & acidic albumin;  Hyaluronic acid-FRET [14]  Hyaluronic acid-azadioxatriangulenium (ADOTA) [15] |  |
|  |  | Sialidase1 | NEU1 | 3.2.1.18 | GH33 | 6p21.33 | X-N-acetyl-α-D-neuraminic acid [16] | BTP3-Neu5Ac [17] |
|  |  | Sialidase4 | NEU4 | 3.2.1.18 | GH33 | 2q37.3 | X-N-acetyl-α-D-neuraminic acid [16] | BTP3-Neu5Ac [17] |
| Lipase |  | Acid lipase | LIPA | 3.1.1.13 | Triacylglycerol lipase | 10q23.31 | X-palmitate | [18] |
|  |  | Phospholipase A2 Group XV | PLA2G15 | 3.1.1.32 | Lipase | 16q22.1 | 1-palmitoyl-2-[^14^C]oleoyl-sn-glycero-3-phosphocholine [19] |  |
| Nuclease | Endo-nuclease | RNaseT2 | RNASET2 | 4.6.1.19 | Ribonuclease | 6q27 | Yeast RNA [20] |  |
|  |  | DNAse II | DNASE2A | 3.1.22.1 | Deoxyribonuclease | 19p13.13 | Lambda DNA;  Plasmid DNA [21] |  |
|  | Exo-nuclease | PLD3 | PLD3 | 3.1.16.1 | Exonuclease | 19q13.2 | [22] |  |
| Protease | Cysteine Protease | Cathepsin B | CTSB | 3.4.22.1 | C01.060 | 8p23.1 | Z-Arg-Arg-AMC | N-Cbz-Ala-Arg-Arg-4-methoxy-2-naphthylamide [23]  (Z-Arg-Arg)_2_-cresyl violet  (Z-Arg)_2_-cresyl violet  [24–26] |
|  |  | Cathepsin C | CTSC | 3.4.14.1 | C01.070 | 11q14.1-q14.3 | H-Gly-Phe-AMC | [24,27] |
|  |  | Cathepsin F | CTSF | 3.4.22.41 | C01.018 | 11q13.1-q13.3 | Z-Phe-Arg-AMC | [24] |
|  |  | Cathepsin H | CTSH | 3.4.22.16 | C01.040 | 15q24-q25 | Z-(L-Arg)-AMC | [24] |
|  |  | Cathepsin K | CTSK | 3.4.22.38 | C01.036 | 1q21 | Z-Gly-Pro-Arg-AMC | [24,28,29]  (Z-Leu-Arg)_2_-cresyl violet |
|  |  | Cathepsin L | CTSL1 | 3.4.22.15 | C01.032 | 9q21-q22 | Z-Phe-Arg-AMC | [24,25,30,31]  (Z-Phe-Arg)_2_-cresyl violet |
|  |  | Cathepsin O | CTSO | 3.4.22.42 | C01.035 | 4q31q32 |  |  |
|  |  | Cathepsin S | CTSS | 3.4.22.27 | C01.034 | 1q21 | Z-Val-Val-Arg-AMC | [24,25,32,33]  (Z-Val-Val)_2_-cresyl violet |
|  |  | Cathepsin V (L2) | CTSV | 3.4.22.43 | C01.009 | 9q22.2 | Z-Phe-Arg-AMC | [24] |
|  |  | Cathepsin W | CTSW | 3.4.22.- | C01.037 | 11q13.1 | Z-Phe-Arg-AMC |  |
|  |  | Cathepsin Z/X | CTSZ | 3.4.18.1 | C01.013 | 20q13.32 | MCA-Arg-Pro-Pro-Gly-Phe-Ser-Ala-Phe-Lys(Dnp)-OH | [25,34] |
|  |  | Legumain | LGMN | 3.4.22.34 | C13.004 | 14q32.12 | Z-Ala-Ala-Asn-AMC | [35] |
|  |  | Gamma-glutamyl hydrolase | GGH | 3.4.19.9 | C26.001 | 8q12.3 | 2,4-diamino-10-methyl-pteroylglutamyl-gamma-glutamate [36] |  |
|  | Serine Protease | Protective protein (cathepsin A) | CTSA | 3.4.16.5 | S10.002 | 20q13.12 | MCA-Arg-Pro-Pro-Gly-Phe-Ser-Ala-Phe-Lys(Dnp)-OH |  |
|  |  | Cathepsin G | CTSG | 3.4.21.20 | S01.133 | 14q12 | N-Succinyl-Ala-Ala-Pro-Phe-AMC | [37] |
|  |  | Pro-Xaa carboxypeptidase | PRCP | 3.4.16.2 | S28.001 | 11q14.1 | MCA-Ala-Pro-Lys(Dnp)-OH |  |
|  |  | Tripeptidyl-peptidase I | TPP1 | 3.4.14.9 | S53.003 | 11p15.4 | L-Ala-Ala-Phe-AMC |  |
|  | Aspartic protease | Cathepsin D | CTSD | 3.4.23.5 | A01.009 | 11p15.5 | MCA-Gly-Lys-Pro-Ile-Leu-Phe-Phe-Arg-Leu-Lys(Dnp)-D-Arg-NH_2_ | Pepstatin A−BODIPY FL  [38] |
|  |  | Cathepsin E | CTSE | 3.4.23.34 | A01.010 | 1q32.1 | MCA-Gly-Lys-Pro-Ile-Leu-Phe-Phe-Arg-Leu-Lys(Dnp)-D-Arg-NH_2_ |  |
|  |  | Napsin | NAPSA |  | A01.046 | 19q13.33 | K(Dabsyl)-TSVLMAAPQ-Lucifer yellow (DS3) [39] |  |
| Aryl Sulphatase |  | N-acetylgalactosamine 6-sulfatase | GALNS | 3.1.6.4 | Arylsulphatase | 16q24.3 | X-N-acetyl-α-D-galactoseaminide-6-sulfate |  |
|  |  | N-acetylglucosamine-6-sulfatase | GNS | 3.1.6.14 | Sulphatase | 12q14.3 | X-N-acetyl-α-D-glucosaminide-6-sulfate; X-β-D-6-sulpho-2-acetamido-2-deoxy-glucopyranoside |  |
|  |  | Arylsulphatase A | ASRA | 3.1.6.8 | Arylsulphatase | 22q13.33 | X-sulfate [40] |  |
|  |  | Arylsulphatase B | ARSB | 3.1.6.12 | Arylsulphatase | 5q14.1 | X-sulfate;  X-N-acetyl-α-D-galactoseaminide-4-sulfate [41] |  |
|  |  | Arylsulphatase G | ARSG | 3.1.6.- | Arylsulphatase | 17q24.2 | X-sulfate |  |
|  |  | Arylsulfatase K | ARSK | 3.1.6.- | Arylsulphatase | 5q15 | X-sulfate |  |
|  |  | N-sulphoglucosamine sulphohydrolase | SGSH | 3.10.1.1 | Sulphatase | 17q25.3 | X-N-sulpho-α-D-glucosaminide |  |
|  |  | Iduronate 2-sulfatase | IDS | 3.1.6.13 | Sulphatase | Xq28 | X-α-l-iduronide-2-sulfate | LysoLive™ lysosomal sulfatase assay kit |
| Thioesterase |  | Palmitoylprotein thioesterase 1 | PPT1 | 3.1.2.22 | Palmitoylprotein thioesterase | 1-p34.2 | X-6-thiopalmitoyl-β-D-glucoside | [42] |
|  |  | Palmitoylprotein thioesterase 2 | PPT2 | 3.1.2.22 | Palmitoylprotein thioesterase | 6p21.32 | X-6-thiopalmitoyl-β-D-glucoside |  |
| Acid ceramidase |  | N-acetylsphingosine amidohydrolase (acid ceramidase) | ASAH1 | 3.5.1.23 | Acid ceramidase | 8p22 | NBD C12 ceramide;  Rbm14-12 [43] | [44,45] |
| Acid Phosphatase |  | Acid phosphatase | ACP2 | 3.1.3.2 | Histidine acid phosphatase | 11p11.2 | X-phosphate; 6-chloro-8-fluoro-4-MU phosphate | [46] |
|  |  | Tartrate-resistant acid phosphatase type 5 | ACP5 | 3.1.3.2 | Histidine acid phosphatase | 19p13.2 | X-phosphate |  |
| Acetyltransferase |  | Heparan-α-glucosaminide N-acetyltransferase | HGSNAT | 2.3.1.78 | Acetyltransfersases | 8p11.21-p11.1 | X-2-amino-deoxy glucose;  BODIPY-Glucosamine [47] |  |
| Monoamine Oxidase |  | L-Aminoacid oxidase | IL4I1 | 1.4.3.2 | Flavin monoamine oxidase | 19q13.33 | Release of H_2_O_2_ against 10 mM L-amino acids [48] |  |
| Sphingomyelinase |  | Sphingomyelin phosphodiesterase | SMPD1 | 3.1.4.12 | Acid sphingomyelise | 11-p15.4 | BODIPY-FL-C12-sphingomyelin [49];  NBD-sphingomyelin;  6-hexadecanoylamino-4MU-phosphorylcholine [50] |  |
| Thiol reductase |  | Gamma-interferon-inducible lysosomal thiol reductase | IFI30 | 1.8.-.- | GILT | 19p13.11 | SDS-denatured affinity-purified rabbit anti-mouse F(ab′)2 [51] |  |

X - chromogen or fluorophore group, used when more than one option of reporter group is available.

# **Protocols**

## High content imaging

In this section, we include information on assays developed for high-content imaging. This approach has several advantages in terms of reproducibility, robustness, obtained information, and analysis. For each assay, we provide the appropriate controls, and considered the possibility of using the assay to screen compound or RNAi libraries. This can be done by using the automated confocal microscopes Opera Fenix and Operetta (PerkinElmer), and the Harmony and Columbus software (PerkinElmer) for image analysis. For more details and information, see the web site of TIGEM High Content Screening Facility: <https://www.tigem.it/research/facilities/core-facilities/high-content-screening>.

### 1.1. Dextran assay

Lysosomes can be loaded with dextran, a fluid phase cargo not degraded by cells, in order to follow endocytic or clearance pathways.

**REAGENTS**

- Dextran-568 (Thermo Fisher, D22912)

**METHOD**

- Seed cells in 96- or 384-well plates and let them grow for 24 h (for compound screening: pre-treat cells with the drugs for 3 h).
- Add Dextran-568 in complete medium (50 ug/ml) for 15 min or 3 h (for clearance assay)
- Wash 2 times in PBS.
- For clearance assay: Incubate cells for 3 h (with the drugs, for compound screening) in a dextran-free medium; compare the number of dextran spots in pulse and chase conditions.

### 1.2. DQ-BSA assay

DQ-BSA is a fluorogenic substrates for lysosomal proteases, useful to measure lysosomal overall degradative capacity.

**REAGENTS**

- DQ-BSA (Thermo Fisher, D12051)

**METHOD**

- Seed cells in 96- or 384-well plates and let them grow for 24 h.
- For compound screening: pre-treat cells with the drugs for 3 h.
- Load cells with 10 µg/ml of DQ-BSA in complete medium for 3 h; when comparing different cell types, lower incubation time points are recommended to evaluate differences in their endocytic capacity; for some disease models, longer incubation times are required to ensure that DQ-BSA reaches lysosomes.

Torin-1, a selective inhibitor of the mTOR kinase, can be used as a positive control. Inhibitors of the vacuolar-type H^+^-ATPase (v-ATPase) such as concanamycin can be used to inhibit lysosomal degradation [52].

### 1.3. GB3 assay

Shiga toxins are a family of protein toxins secreted by certain types of bacteria. Shiga toxin (STX) subunit B recognizes and binds globotriaosylceramide (Gb3). This method uses a Cy3-labelled STX and a LAMP1 antibody to detect Gb3 accumulation in lysosomes.

**REAGENTS**

- Cy3-labelled STX subunit B
- Anti-LAMP1 antibody (Santa Cruz Biotechnology, sc-20011)
- Blocking buffer [0.1% (w/v) saponin, 0.5% (w/v) BSA and 50 mM NH_4_Cl in PBS]

**METHOD**

- Seed cells in 96- or 384-well plates and let them grow for 24 h.
- For compound screening: pre-treat cells with the drugs (10 μM or DMSO 0.1% in complete medium and incubate 48 h).
- Wash cells in PBS and fix in 4% PFA in PBS for 10 min at RT.
- Wash twice in PBS and block for 1 h in blocking buffer at RT.
- Incubate Cy3-labelled STX subunit B to detect Gb3 with primary antibody anti-LAMP1 for 2 h in blocking buffer at RT.
- Incubate cells with secondary antibodies for 60 min.
- Assess the accumulation of Gb3 by analysing the area of GB3 spots (detected with STX) within lysosome spots (detected by LAMP1 staining) normalized to the total area of lysosome spots [52].

PDMP (glucosylceramide synthase inhibitors) can be used as a positive control for STX reduction.

### 1.4. Magic Red assay

Magic Red cathepsin assay kits enable the *in vitro* quantitation and monitoring of intracellular cathepsin B activity over time. The Magic Red reagent is a substrate that emits red fluorescence upon cleavage by active cathepsins.

**REAGENTS**

- Bafilomycin (Sigma, B1793)
- Magic Red cathepsin B kit (ImmunoChemistry Technologies, 937)
- Hoechst 33342 (Sigma, B2261)

**METHOD**

- Seed cells in 96- or 384-well plates and let them grow for 24 h.
- For compound screening: pre-treat cells with compounds in complete medium for 3-12 h.
- Stain cells with Magic Red cathepsin B kit for 30 min at 37°C.
- Rinse cells briefly with PBS.
- Stain cells with 1 µg/ml Hoechst for 10 min.
- Cathepsin B activity is measured by analyzing the number of spots and their intensity.

Bafilomycin (vacuolar-type H^+^-ATPase inhibitor) can be used as a negative control for cathepsin activity.

### 1.5. Lysosome membrane permeabilization assay

In this assay, Acridine Orange is used to evaluate both the integrity of the lysosomal membrane and the acidification of this compartment by a direct imaging approach. AO is a lysosomotropic fluorescent probe that emits red light in acidic compartments and switches to green light in alkaline compartments.

**REAGENTS**

- Acridine Orange (Thermo Fisher, A1301)

**METHOD**

- Seed HeLa cells in 384-well plates and let them grow for 24 h.
- Stain cells with 5 g/ml Acridine Orange in complete medium for 15 min.
- Wash cells 3 times with PBS (with Ca^2+^ and Mg^2+^).
- After 15 min, several parameters can be measured: red puncta (lysosomes), intensity. of red fluorescence and intensity of green fluorescence; the ratio of red/green emission can be used as a measure of lysosomal membrane permeabilization [53].

As a positive control of lysosomal membrane permeabilization, control cells are treated with the vacuolar-type H^+^-ATPase inhibitor concanamycin A.

## Proximity ligation assay

The method uses antibodies from two species (mouse and rabbit) to recognize proteins on two different organelles (lysosome and endoplasmic reticulum). If the two antibodies are in very close apposition (less than ∼40 nm; *e.g.*, at membrane contact sites), secondary antibodies conjugated to complementary oligonucleotides are amplified, incorporating a fluorescent nucleotide (Texas Red), to generate a fluorescent signal visible by light microscopy (excitation: 594 nm, emission: 624 nm).

**REAGENTS**

- Paraformaldehyde (PFA): 36% formaldehyde (TAAB, F003)
- Triton X-100 (Sigma, T8787)
- Rabbit anti-LAMP1 antibody (Cell Signaling, 9091)
- Mouse anti-VAPA antibody (Santa Cruz, sc-293278)
- Duolink in Situ Red Starter Kit Mouse/Rabbit (Sigma, DUO92101)

**METHOD**

Day 1

- Wash cells in PBS and fix in 4% PFA in PBS for 20 min at RT.
- Wash 2 times in PBS and quench in 15 mM glycine for 10 min at RT.
- Permeabilise in 0.1% Triton X-100 in PBS for 10 min at RT.
- Wash 2 times in PBS and block for 1h in 1% BSA in PBS at RT.
- Incubate in primary antibody [*e.g.*, rabbit anti-LAMP1 (1:160) and mouse anti-VAPA (1: 8] diluted in 1% BSA, overnight at 4°C.

Day 2

- Wash 2 x 5 min in Wash Buffer A at RT.
- Incubate with Duolink PLA probe (anti-rabbit PLUS and anti-mouse MINUS), diluted 1:5 in Duolink antibody diluent in a humidity chamber at 37°C for 1 h.
- Wash 2 x 5 min in Wash Buffer A at RT.
- Incubate with ligase, diluted 1:40 in 1 x ligation buffer in a humidity chamber at 37°C for 30 min.
- Wash 2 x 5 min in Wash Buffer A at RT.
- Incubate with polymerase, diluted 1:80 in 1 x amplification buffer in a humidity chamber at 37°C for 100 min.
- Wash 2 x 10 min in 1x Wash Buffer B at RT.
- Wash 1 x 1 min in 0.01x Wash Buffer B at RT.
- Mount onto slide for imaging or storage in the dark at 4°C.

## Immuno-electron microscopy: pre-embedding labelling

Like immunofluorescence, this method involves antibody labelling of permeabilized cells, but instead of labelling with fluorescence, the secondary antibody is conjugated to a nanogold particle, which is later expanded in an enhancement step, during preparation for conventional EM. To balance morphological preservation with antibody accessibility, this method is best suited to labelling the cytoplasmic domain of lysosomal membrane proteins.

**REAGENTS**

- Paraformaldehyde (PFA): 36% formaldehyde (TAAB, F003)
- Triton X-100 (Sigma, T8787)
- Nanogold secondary antibodies [Universal Biologicals, NP-2002 (anti-mouse); NP-2004 (anti-rabbit)]
- 25% glutaraldehyde (Agar Scientific, AGR1012)
- Nanoprobes gold enhance EM plus kit (Universal Biologicals, 2114)
- Sodium cacodylate (Agar Scientific, AGR1501)
- Osmium tetroxide (Agar Scientific, AGR1019)
- Potassium ferricyanide (TAAB, F003)
- UAzero (Agar Scientific, AGR1000)
- Propylene Oxide (PO, Agar Scientific, AGR1080)
- TAAB-812 (TAAB, T022)
- Dodenyl succinic anhydride (DDSA; Agar Scientific, R1051)
- Methyl nadic anhydride (MNA; Agar Scientific, R1081)
- 2,4,6-Tri(dimethylaminomethyl) phenol (DMP30; Agar Scientific, AGR1065)
- PHEM buffer (60 mM PIPES, 25 mM HEPES, 10 mM EGTA, 2 mM MgCl_2_ pH 7)
- Block buffer (1% BSA/0.1% Ac-BSA in PHEM)

**METHOD**

- Fix cells in 4% PFA/PHEM for 1 h at RT and store in 0.5% PFA/PHEM in the fridge
- Wash in PHEM and quench 2x in 20 mM glycine/PHEM.
- Permeabilise in 0.05% Triton X-100/PHEM in for 5 min on ice.
- Wash 2x in PHEM and block in block buffer for 45 min at RT.
- Incubate with primary antibody in block for 1 h at RT.
- Wash in block buffer 4x.
- Incubate in nanoAu-secondary antibody (1:200) in block buffer for 45 min at RT.
- Wash in block buffer and then 3x in PBS.
- Fix in 2% PFA/2% glutaraldehyde in 0.1 M cacodylate buffer for 30 min at RT (in the fume hood).
- Remove fixative to aldehyde waste bottle and wash 3x in PBS.
- Quench 2x in 200 mM glycine/PBS and wash 2x in water.
- Prepare gold enhance mix : Mix 1xA:5xB at RT x 5min

Add 3xC+3xD.

- Invert coverslips onto 30 μl drops enhance mix on parafilm on ice for 8-10 min.
- Stop reaction with 1% acetic acid, wash in water, then 3x PBS, then 0.05M cacoldylate buffer.
- Incubate with 1% osmium tetroxide/1.5% potassium ferricyanide on ice for 1 h (in the fume hood).
- Prepare TAAB-812 resin: Mix 19 ml TAAB-812 with 9 ml DDSA, 12 ml MNA and 0.8 ml DMP-30 for 1-4 h rotating at RT.
- Remove osmium plus first wash into osmium waste bottle (in the fume hood).
- Wash 3x in PBS (store in fridge if convenient).
- Wash 3x in water.
- Incubate in UAzero in dark at RT for 1 h.
- Wash 3x in water.
- Dehydrate in ethanol: 2 min in 70% ethanol, 2 min in 90% ethanol, 2x 10 min in 100% ethanol.
- Incubate in PO:TAAB-812 resin (1:1) for 1 h at RT (in the fume hood).
- Incubate in TAAB-812 resin for 1 h at RT in the fume hood.
- Invert beam capsule freshly filled with TAAB-812 resin onto coverslips and bake at 65°C overnight.
- Plunge baked stub/coverslip into liquid nitrogen directly from oven to remove coverslip.
- Remove beam capsule and cut sections to image by EM.

## Loading lysosomes with BSA-gold for electron microscopy

Similar to dextran loading (see 1.1), lysosomes can be loaded with BSA-gold conjugate via fluid phase endocytosis for identifying the terminal endocytic organelles by EM. Depending on the cell type, a 1-4 h loading of BSA-gold reagent is performed in complete medium. After washing cells thoroughly, the BSA-gold should clear intermediate endocytic compartments and be totally confined to lysosomes within a 2-4 h chase, although this is also somewhat cell-type dependent. The loaded lysosomes can be identified in EM by the presence of aggregated electron-dense gold particles. BSA-gold conjugates are available in different gold sizes, although efficiency of uptake is inversely proportional to the size of the gold selected. Therefore, 5 nm and 10 nm gold sizes are generally preferred for fluid-phase loading.

**REAGENTS**

- BSA-gold conjugate, 5 nm (UMC Utrecht CMC, BSAG 5 nm)
- Paraformaldehyde (PFA): 36% formaldehyde (TAAB, F003)
- 25% glutaraldehyde (Agar Scientific, AGR1012)

**METHOD**

- Remove buffering BSA by ultracentrifugation/dialysis and dilute to optical density = 5 in complete media, both as per manufacturer’s instructions.
- Add diluted BSA-gold to cells for 1-4 h at 37^o^C to load the endocytic pathway.
- Wash 3 times in PBS/media to remove non-internalised BSA-gold.
- Chase cells for 2-4 h in media free of BSA-gold at 37^o^C to allow BSA-gold to reach the lysosomal compartment.
- Fix in 2% PFA/2% glutaraldehyde in 0.1 M cacodylate buffer and proceed with specimen processing for EM.

Lysosomes are identified in EM as relatively electron dense organelles (membrane whorls may be present) containing aggregated gold particles.

## Ratiometric measurement of fluorescent conjugates of dextran to quantify lysosome luminal pH

This method is based on the ratiometric measurement of pH-sensitive and pH-insensitive fluorescent conjugates of dextran. The ratiometric fluorescence is indicative of relative differences in pH. An *in situ* calibration curve of the signal should be used to convert the fluorescence ratio in absolute pH values.

**CELL LINES**

- RAW264.7 murine macrophage-like cells (ATCC TIB-71)

**REAGENTS**

- pHrodo™ Green Dextran, 10,000 M.W. (Thermo Fisher) and Alexa Fluor 647-dextran 10,000 M.W. (Thermo Fisher) dissolved in sterile 1X PBS at a concentration of 25 mg/ml.
- Hank’s balanced salt solution (HBSS) with Ca^2+^ and Mg^2+^, without phenol red (Thermo Fisher, 14025092)
- Nigericin (free acid, Thermo Fisher, N1495)

**METHOD**

**Loading cells with pHrodo- and Alexa Fluor 647-dextran**

- Forty-eight hours prior to pH assessment, seed RAW264.7 cells in a Nunc™ Lab-Tek™ Chamber Slide (Thermo Fisher) containing complete medium.
- Add pHrodo-dextran (final concentration: 50 μg/ml) and Alexa Fluor 647-dextran (final concentration: 50 μg/ml) to each chamber and incubate for 16 h at 37^o^C.
- Wash the cells three times with 1X PBS containing Ca^2+^ and Mg^2+^ to remove any residual fluorescent dextran.
- Incubate the cells in complete medium for 3 h at 37^o^C to allow the fluorescent-dextrans to traffic to lysosomes.

A sufficient chase time is a key aspect of this assay, as imaging too soon after loading may result in detection of compartments that are not lysosomes. Therefore, for each cell type, prior to pH assessment, it is necessary to optimize the chase time. For this purpose, preliminary experiments using lysine-fixable dextran chased for various times and immuno-labelled with known markers of lysosomes, such as LAMP1 must be performed.

**Lysosome pH measurement**

- Remove the culture medium and add HBSS with Ca^2+^ and Mg^2+^ and without phenol red to RAW264.7 cells.
- Measure the fluorescence intensity ratio between pHrodo- and Alexa Fluor 647-dextran.

***In situ* calibration**

The most straightforward procedure is to perform a calibration (*i.e*., establish the relationship between fluorescence ratio and the pH of the surrounding medium) of the free dye in buffers of varying pH, *in vitro*. However, this approach assumes that the dye behaves identically in the cellular compartment of interest as it does in solution; this is often not the case, due to interactions of the fluorophore with cellular constituents and/or with itself in the confined space of the organellar lumen. Therefore, it is more accurate to perform an *in situ* calibration.

**CALIBRATION BUFFERS**

Calibration buffers for measurement of lysosomal pH consist of 140 mM KCl, 1 mM MgCl2, 1 mM CaCl_2_, 5 mM glucose, 20 mM HEPES for buffers at pH 7.0–8.0, 20 mM MES (Sigma) for buffers pH 4–6.5. Nigericin, an H+ ionophore (dissolved in ethanol at a concentration of 10 mg/ml) should be added immediately prior to calibration.

**METHOD**

- Wash the cells once with the most alkaline of the pH calibration curve buffers and incubate with an appropriate volume of the calibration buffer in presence of Nigericin, at a final concentration of 10 µM. All solutions must be preheated and maintained at 37°C.
- After 2 min incubation, image cells.
- Rinse cells once with 1X PBS and twice with the next pH calibration curve buffer.
- Repeat steps 3–5 until images have been collected for cells incubated in at least five of the pH calibration curve buffers to construct a full calibration curve where the experimental values will be interpolated. Ensure to proceed from most alkaline (pH 8) to most acidic (pH 3.5).
- Calculate the average pHrodo signal:Alexa Fluor 647 signal ratio for each pH calibration curve buffer.
- Fit the data to generate a pH calibration curve for use in determining pH-values based on experimental data.
- The experimentally determined lysosome pH can then be determined by interpolating the ratio in the calibration curve generated.

## Monitoring of endo-lysosomal Ca^2+^ nanodomains

Because of the small size of the endo-lysosomal Ca^2+^ store and the limited diffusion of cytoplasmic Ca^2+^, the local Ca^2+^ nanodomains produced by endo-lysosomal Ca^2+^ channels can be almost invisible in global cytoplasmic recordings and require specific approaches to monitor them. A favoured strategy is to fuse a genetically encoded Ca^2+^ indicator (GECI) to the ion channel of interest, thus locating the GECI at the very site of the privileged high Ca^2+^ nanodomains around the mouth of the channel.

Although a technically straightforward approach, there are pitfalls in assuming that this alone will specifically monitor lysosomal Ca^2+^ nanodomains. A number of technical considerations need to be taken into account first.

- GECI fusion at the N or C terminus? Two considerations:
  - From the known topology of the channel, select a terminus that is on the cytosolic face of the endo-lysosomal membrane.
  - Check that the GECI fusion protein retains function. For example, fusion of fluorescent proteins to the N terminus of TPC1 [56] inhibits channel activation, whereas C-terminal fusions remain fully functional.
- Select a GECI with a K_d_ that matches the locally high [Ca^2+^] of the local domain. A low-affinity GECI is preferred, and one that is bright and with a decent dynamic range.
- Confirm that the K_d_ of the GECI is not altered by fusion with the channel.
- Channel-GECIs will also detect Ca^2+^ emanating from other, larger Ca^2+^ sources (*e.g*., the ER, Ca^2+^ influx), and this bystander ‘spillover’ may contaminate the endo-lysosomal Ca^2+^ signal. The contribution of this component must be determined empirically for each cell type and stimulus by:
  - Comparing with unfused, cytosolic GECI (to determine the degree to which global Ca^2+^ signals bleed into the GECI signal).
  - Reciprocally manipulating Ca^2+^ release from the endo-lysosomal channel versus other Ca^2+^ sources (see below).
- The illustrative example outlined below is from an investigation of the role of endolysosomal Ca^2+^ signaling during phagocytosis in macrophages [57].

**CELL LINES**

- RAW 264.7 murine macrophage cells
- COS-7 African green monkey kidney fibroblast-like cell line
- HeLa human cervical cancer cell line

**REAGENTS**

*DNA Plasmids and Transfection*

- C-terminus tagged TPCs fused to low-affinity green indicator, G-GECO1.2 (K_d_ = 1.2 µM ) (Addgene, 32446 [58])
- High-purity DNA for transfection (macrophages are sensitive to endotoxin)
- JetPRIME (Polyplus Transfection, 101000027)

*Ca^2+^ Imaging*

- CellView 4‐compartment glass‐bottom dishes (Greiner Bio‐One, 627871)
- Extracellular medium (ECM) composition is prepared with or without Ca^2+^. Core composition: 121 mM NaCl, 5.4 mM KCl, 0.8 mM MgCl_2_, 6 mM NaHCO_3_, 25 mM HEPES, 10 mM Glucose, pH 7.4
  - Ca^2+^-replete ECM is supplemented with 1.8 mM CaCl_2_ and RPMI Essential Amino Acids (Sigma)
  - Ca^2+^-free ECM contains no added Ca^2+^ and is supplemented either with 1 mM EGTA or 100 µM EGTA
- Red Ca^2+^ dye: Calbryte-590/AM (AAT Bioquest, 20701)
- Ionomycin (free acid, Merck, **407950**)
- Trans Ned-19 (Enzo Life Sciences, ALX-270-503-MC05 or Cayman Chemical, 17527)
- CPA (Merck) and thapsigargin (Merck, **T9033**)
- EGTA/AM (Merck, **324628**)

*Stimuli*

- For F_c_-receptor (FcR) stimulation, silica beads (3- or 6-µm diameter) are opsonized with mouse IgG using standard protocols [59] and stored at 4°C in PBS containing 2% sodium azide. FcR recruits both endo-lysosomal channels (TPCs, TRPMLs) and IP_3_Rs [59].
- Purinoceptor agonist, UTP, 100 mM in water (Merck). Only recruits IP_3_Rs [59].

**METHODS**

**Cell transfection**

- RAW 264.7 cells are seeded into 4-chamber dishes (~2% of a confluent T25 flask per dish) and left overnight in 500 µl of DMEM
- The following morning, cells are transfected. Per quarter chamber, 0.4-0.5 µg of DNA encoding the GECI is mixed with JetPRIME reagent in a ratio of 2:1, according to the manufacturer’s protocol, and delivered dropwise in 50 µl. Cells are returned to the incubator.
- After 4-6 hours, medium is replaced with fresh DMEM and left overnight.
- Transfection efficiency is typically ~30%.

**Ca^2+^ imaging**

- If simultaneously monitoring G-GECO1.2 and cytosolic Ca^2+^, load cells with a red chemical indicator, Calbryte-590/AM.
- Remove DMEM and replace with ECM (+ Ca^2+^) containing 2 µM Calbryte-590/AM (2 mM stock in DMSO) together with 0.03% Pluronic F127 (10% w/v in DMSO). Incubate for 50 min at RT.
- Wash with ECM (+Ca^2+^) and leave for a further 10-15 min to de-esterify.

Most Ca^2+^ imaging experiments are conducted in Ca^2+^-free medium to eliminate Ca^2+^ influx and thereby monitor intracellular Ca^2+^ release in isolation. All imaging media are pre-warmed in a 37°C water bath.

- Immediately before use, wash cells 1x in 300 µl of ECM (1 mM EGTA).
- Wash cells 3x in ECM (100 µM EGTA). Final volume of 300 µl per quarter chamber.
- The chamber is mounted in an in-house thermostatically regulated stage-holder (chamber ECM temperature ~32°C).
- Cells are imaged fluorescently using standard green/red filter sets and a 20x or 60x objective. A resonant scanning confocal microscope (Nikon A1R) can be used to capture a single-or dual-channel image every 500-564 ms. This rate allows accurate determination of the peak of the rapid Ca^2+^ transients observed in macrophages.

Ca^2+^ signals are stimulated either by activating FcRs or purinoceptors. For FcR activation, 5-15 µl IgG-coated beads are added in a total volume of 30 µl ECM (+100 µM EGTA) and allowed to drop onto the cells; for purinoceptors, UTP is added in 200 µl ECM (+100 µM) at 100 µM final.

- Amplitudes are corrected for the variation in G-GECO1.2 expression by determining the maximum fluorescence (F_max_) at the end of each run. The GECI is saturated by the addition of 1-2 µM ionomycin plus 10 mM CaCl_2_.
- The responses of each single cell are expressed as a percentage of the maximum excursion between the initial fluorescence (F_0_) and the F_max_. i.e. 100 x (F_t_-F_0_)/(F_max_-F_0_) where F_t_ is the fluorescence at any time.
- Measure the peak amplitude of up to the first 3 spikes (although not all cells show multiple transients).

**Measuring the K_d_ of GECIs**

The K_d_ for Ca^2+^ of G‐GECO1.2 proteins can be determined *in situ* in permeabilized cells that are easy to transfect (*e.g*., HeLa or Cos-7). In brief, the cytoplasm is equilibrated with an intracellular‐like media (ICM) buffered to different free [Ca^2+^]. The basic ICM is: 10 mM NaCl, 140 mM KCl, 20 mM HEPES, pH 7.2 at RT. To generate different free [Ca^2+^], a constant excess of 5 mM Ca^2+^ chelator (DiBrBAPTA, BAPTA, HEDTA or nitrilotriacetic acid) is added to ICM and different amounts of total Ca^2+^ (0-4 mM) are added. All solutions are readjusted to pH 7.2 since Ca^2+^ binding displaces H^+^ from the chelator. The free [Ca^2+^] is calculated from known total concentrations of chelator and Ca^2+^ using programs such as Winmax chelator (Dr C. Patton, Stanford, USA). A range of Ca^2+^ chelators are required because each has a different Ca^2+^ affinity and buffers Ca^2+^ over different [Ca^2+^] ranges. We typically prepare Ca^2+^ solutions a decade either side of the K_d_ using half-log values.

- Transiently transfected cells expressing cytosolic G‐GECO1.2 or TPC2‐G‐GECO1.2 are washed 3× with an ICM at a fixed free [Ca^2+^], and the final ICM addition is supplemented with 30 μM sulforhodamine B (extracellular) as a permeabilization marker.
- Cells are imaged with standard green and red channels on a confocal laser-scanning microscope. An image is acquired every 2-3 s.
- The plasma membrane is discretely permeabilized by the addition of 50-60 μg/ml β‐escin for ~5 min, breaching indicated by the entry of small MW marker, sulforhodamine B, while the large MW protein G‐GECO1.2 is retained (even the cytosolic form).
- As extracellular Ca^2+^ equilibrates, the GECI fluorescence reaches a plateau when it is in equilibrium with the extracellular ICM and this value was normalized to the basal fluorescence prior to permeabilization (*F*_0_). A plot of the calculated free [Ca^2+^] versus *F*/*F*_0_ values is fitted to a sigmoidal dose–response to determine the *in situ K* _d_ of G‐GECO1.2 either cytosolic or fused to TPC2.
- Fusion of G‐GECO1.2 to the C-terminus of TPC2 does not significantly affect its Ca^2+^ binding [59].

#### **Controls**

##### Global versus local components

- Stimulate cells with beads.
- To determine if the *position* of the GECI is important, compare cytosolic G-GECO1.2 and the TPC2-G-GECO1.2 fusion.
- Only the TPC2-G-GECO1.2 should detect bigger Ca^2+^ nanodomains and be larger than the unfused G-GECO1.2 (typically twice the amplitude).

##### **Ca^2+^ sources**

Verify which Ca^2+^ source(s) the TPC2-G-GECO1.2 is detecting. That is, are the bigger TPC2-G-GECO1.2 responses due to lysosomal Ca^2+^ release or ER spillover? Recall that beads (but not UTP) activate the TPC2 pathway.

- If TPC2 activation is occurring, then the response should be reduced by inhibiting TPC2 function:
  - Pre-incubate cells with the NAADP antagonist trans Ned-19 (10-20 µM for 30-50 min).
  - Use a pore-dead TPC2 mutant (D276K) [59] [60].
- To assess the contribution of ER spillover:
  - Stimulate Ca^2+^ release from the ER only. Add the purinoceptor agonist, UTP (to only recruit the IP_3_ pathway; no activation of the TPC pathway). In contrast to beads, the TPC2- G-GECO1.2 response should be the same amplitude as the global response measured with cytosolic G-GECO1.2 (and should be unaffected by Ned-19).
  - Conversely, remove the ER component in two ways:
- Pre-deplete the ER with SERCA inhibitors (30-100 µM CPA or 1 µM thapsigargin) for 5-10 min in Ca^2+^-free medium (ECM + 100 µM EGTA). Imaging must also be performed in Ca^2+^-free medium to eliminate the substantial store-operated Ca^2+^ entry. In parallel, it is essential to verify that ER depletion has indeed occurred (*e.g.*, with 1 µM ionomycin in Ca^2+^-free medium) [59] .
- Buffer the global (ER) Ca^2+^ signal by loading the cytosol with the slow Ca^2+^ buffer, EGTA/AM. This should inhibit the global rise but leave the lysosomal Ca^2+^ nanodomains untouched [59].
- Neither strategy should greatly affect the amplitude of the TPC2-G-GECO1.2 activated by beads.

**Acknowledgement**

This protocol was compiled by Dr. Anthony J. Morgan and Dr Lianne C. Davis, Department of Pharmacology, University of Oxford OX1 3QT. Email: anthony.morgan@pharm.ox.ac.uk

## Use of an LSD model (GLA-KO) to assess lysosome biogenesis

A cell model of Fabry Disease (Hela-*GLA* KO) is used as a tool to analyse lysosomes that have been formed through lysosome biogenesis. *GLA*-KO cells display lysosomal Gb3 storage that can be visualized by using fluorescently-labelled Shiga Toxin subunit B. Upon lysosome biogenesis induction (by prolonged amino acid deprivation), old lysosomes contain Gb3 are labelled with Cy5-conjugated Shiga Toxin, whereas newly formed lysosomes that have not accumulated Gb3, are not labelled with Cy5-conjugated Shiga Toxin. This method strictly relies on the possibility of labelling and visualising the storage material within lysosomes and thus, cannot be translated to other LSD cell models for which microscopic visualisation of the stored material is not possible.

**CELL LINES**

- HeLa (*GLA*-KO) cells

**REAGENTS**

- HBSS (Gibco, #14025092)
- Paraformaldehyde-Aqueous Solution EM Grade (Electron Microscopy Sciences, 157-8)
- Anti-LAMP1 antibody (Developmental Studies Hybridoma Bank, H4A3)
- Blocking buffer [0.1% saponin, 0.5% BSA and 50 mM NH_4_Cl in PBS]
- Cy5-labelled Shiga Toxin subunit B
- Goat anti-Mouse IgG (H+L) cross-adsorbed secondary aAntibody, Alexa Fluor 488 (Invitrogen, A-11001)

**METHOD**

- Grow HeLa (*GLA*-KO) cells in either growth medium or HBSS (to stimulate lysosome biogenesis) for 24 hours on glass coverslips.
- Fix in PFA 4% for 10 min.
- Permeabilize with blocking buffer.
- Incubate with anti-LAMP1 antibody (1:2,000 or 30 ng/ml) in blocking buffer for 2 h.
- Wash 3x with PBS and incubate with Alexa Fluor 488-conjugated anti-mouse antibody (4μg/ml) and Cy5-conjugated Shiga Toxin (1:50,000 or 150 ng/ml) for 45 min.

For the evaluation of old and new lysosomes (generated by lysosomal biogenesis), LAMP1- or Shiga Toxin-positive structures larger than 0.01 μm^2^ are identified and counted. LAMP1-positive and Shiga Toxin-negative structures increase in conditions in which lysosomal biogenesis is stimulated (*i.e*., incubation with HBSS) and represent *bona fide* newly-formed lysosomes.

## Analysis of TFEB subcellular localization

This method describes how to perform immunofluorescence analysis of endogenous TFEB and determine its subcellular localization, which is a readout of TFEB activity. It involves the use of a TFEB antibody that works well in most human cell lines, although TFEB expression levels should be taken into consideration. It is also important to consider that TFEB localization and activity are strictly dependent on nutrient availability and stress conditions. Therefore, cell culture conditions have to be carefully controlled. Nutrient synchronization (*e.g.*, starvation/re-feeding) may help reducing variability.

**REAGENTS**

- Paraformaldehyde (PFA): 36% Formaldehyde (TAAB, F003)
- Triton X-100 (Sigma, T8787)
- Rabbit anti-TFEB antibody (Cell Signaling, 4240)
- Donkey anti-Rabbit, Alexa Fluor Plus 488 (Thermo Fisher, A32790)
- Blocking buffer (3% BSA, 0.02% saponin in PBS)

**METHOD**

Day 1

- Seed cells in a Nunc Lab-Tek Chamber Slide (Thermo Scientific) at 70-80% confluency.

Day 2

- Fix cells in 4% PFA for 10 min at RT.
- Wash twice with PBS.
- Permeabilize with 0.1% triton X-100 for 7 min (increasing this incubation time may affect membrane integrity of other cellular organelles).
- Wash twice with blocking buffer.
- Incubate cells with blocking buffer for 1 h at RT.
- Incubate with primary anti-TFEB antibody (Cell Signaling, 4240, diluted 1:100 in blocking solution) at 4°C overnight.

Day 3

- Wash cells 3 x with blocking buffer.
- Incubate with secondary antibody (diluted 1:1,000 or 2 ug/ml in blocking solution) for 1 h at RT.
- Wash cells 3 x with blocking buffer.

## Lysosome immunopurification (LysoIP)

This technique allows rapid and highly reproducible isolation of intact and pure lysosomes. LysoIP relies on the overexpression of a lysosomal resident protein, known as TMEM192 (transmembrane protein 192), fused to 3x-HA tag (anti-human influenza virus hemagglutinin). Anti-HA magnetic beads allow the isolation of lysosomes from whole cell lysates for further processing, including Western blot (WB) and mass spectrometry analysis.

**CELL LINES**

- RCS Swarm chondrosarcoma chondrocyte line [53] stably expressing TMEM192-3xHA
- HeLa human cervical cancer cell line stably expressing TMEM192-3xHA
- ARPE-19 human retinal pigment epithelium cell line stably expressing TMEM192-3xHA

**REAGENTS**

- PhosphoSTOP (Roche, 4906837001)
- EDTA-free protease inhibitor tablets (Roche, 11873580001)
- Pierce anti-HA-magnetic beads (Thermo Fisher, 88837)
- Colorimetric BCA protein assay kit (Pierce, 23227)
- LysoIP Buffer (25 mM KCl, 50 mM KH_2_PO_4_, 50 mM K_2_HPO_4_, pH 7.2)
- LysoIP Washing Buffer (25 mM KCl, 50 mM KH_2_PO_4_, 300 mM NaCl, pH 7.2)
- LysoIP Elution Buffer I (25 mM KCl, 50 mM KH_2_PO_4_, 0,5% IGEPAL - Sigma, I3021 - pH 7.2)
- LysoIP Elution Buffer II (50 mM Tris HCl, 2% SDS, pH 7.5)

**METHOD**

- Forthy-eight hours prior to LysoIP, seed approximately 20 x 10^6^ control and stably expressing TMEM192-3xHA cells in a 150 mm dish (Corning) containing complete medium.
- Wash twice with 1X PBS containing Ca^2+^ and Mg^2+^.
- Detach cells with trypsin-EDTA.
- Pellet cells and wash with ice cold PBS supplemented with PhosphoSTOP and EDTA-free protease inhibitor tablets at 1x final concentration and resuspend in 1 ml ice-cold LysoIP buffer.
- Gently homogenize cells on ice with ~40 strokes, using a cell homogenizer (#H8 cell homogenizer, #S8 4-10 and #S8 12-18 tungsten carbide standard ball, Isobiotech - size 10 of the tungsten carbide ball for RCS cell lysis); hand-potter (PerkinElmer, 07-358029) for ARPE-19 cell lysis or 27G syringe needle (BD Microlance, 23747) for HeLa cell lysis.
- Centrifuge for 10 min at 1,500 x*g* and 4°C to pellet nuclei and cell debris.
- Collect the post-nuclear supernatant (PNS) and precipitate ~1.5 mg of lysate using pre-washed LysoIP buffer and anti-HA-magnetic beads for 90 min at 4°C (with rotation).
- Wash precipitate twice in LysoIP buffer for 10 min, at 4°C using a DynaMag Magnet; the collection tube should be changed after the first wash to minimize undesirable impurities without affecting the yield of purified lysosomes.
- Wash 4 times with LysoIP washing buffer for 5 min and at RT to disrupt non-specific binding.
- Wash twice with LysoIP buffer to revert the low salt concentration that might interfere with subsequent procedures.
- Resuspend whole cell lysate and precipitated lysosomes in 1 v/v 2x Laemmli sample buffer and denature at 95°C for 5 min. Alternatively, bound lysosomes could be eluted by incubation with 3x-HA peptide.
- Resolve samples in 4-15% SDS-PAGE.
- Perform WB for the proteins of interest.

**Mass spectrometry analysis**

- For protein extraction from lysosomes, resuspend beads with bound lysosomes in LysoIP elution buffer I for 30 min at 4°C (rotating).
- Remove beads with DynaMag Magnet and resuspend in LysoIP elution buffer II.
- Resolve 4-15% SDS-PAGE and stain gel with Instant Blue staining to check the quality of the LysoIP.
- Precipitate proteins obtained from elution I with 4 volumes of ice-cold acetone to 1 volume of sample and store overnight at -20°C.
- Resuspend precipitated proteins in 30 μl of GnHCl buffer (6 M guanidine hydrochloride, 50 mM Tris pH 8.5, 5 mM TCEP, 20 mM chloro-iodoacetamide).
- Incubate at 95°C for 10 min to reduce and alkylate the proteins.
- For label-free quantification-based proteome analysis, dilute samples 1:2 in 50 mM ABC (ammonium bicarbonate) buffer.
- Digest proteins in-solution with endopeptidase sequencing-grade Lys-C (1:100 ratio) for 3 h at 37°C.
- Dilute samples 1:3 in 50 mM ABC buffer.
- Digest proteins with trypsin (1:100 ratio) overnight at 37°C.
- Block enzymatic activity with 1% (final concentration) of trifluoroacetic acid (TFA) and collect peptide mixtures concentrated and desalted using the Stop and Go Extraction (STAGE) technique [54].
- Perform LC MS/MS analysis.

## Determination of cathepsin D activity in multiwell format using an artificial MCA-Dnp substrate

Cathepsin D is one of the most abundant lysosomal hydrolases and is often used as a lysosomal marker. The activity of this protease can be easily assayed in cellular/tissue lysates in a multiwell format by using an internally quenched peptide substrate. The MCA group of the substrate becomes fluorescent following the hydrolysis of the quencher Dnp group.

**REAGENTS**

- Lysis and reaction buffer (sodium acetate 50 mM pH 5.5, 0.1 M NaCl, 1 mM EDTA and 0.2% Triton X-100)
- Leupetin (cysteine protease inhibitor; Sigma, L2884)
- MCA-Gly-Lys-Pro-Ile-Leu-Phe-Phe-Arg-Leu-Lys(Dnp)-D-Arg-NH_2_ substrate (Enzo Life Sciences, P-145)
- 7-Methoxycoumarin-4-acetic acid (MCA; Sigma, M5140)

**METHOD**

- Lyse the cells or tissues for 30 min at 4°C in lysis buffer (with shaking).
- Clear the lysates by centrifugation (10 min at 16,000 x*g*) and use the supernatant directly for determination of proteolytic activity.
- Dilute the sample in lysis buffer to the desired final protein concentration (protein input typically varies between 1-10 µg of total protein depending on the sample).
- In a 96-well plate (Costar 96-Well Black Polystyrene Plate, 734-1663), incubate 2 μl of diluted lysate at 37°C for 60-120 min in lysis buffer (100 μl) containing 10 µM of MCA-Gly-Lys-Pro-Ile-Leu-Phe-Phe-Arg-Leu-Lys(Dnp)-D-Arg-NH_2_ and 25 µM leupeptin (to eliminate residual hydrolysis by cysteine proteases).
- Measure MCA-emitted fluorescence at 460 nm (with an excitation wavelength of 322 nm).
- Subtract to the measured activities the background (without the biological sample).
- For relative enzymatic activity, normalize the fluorescence to the protein content and to the control values. For determination of specific enzymatic activity (nmol/µg/h) an MCA calibration curve should be performed (*e.g.*, 0.1 – 10 nmol) to determine the fluorescence emitted by 1 nmol of MCA

Positive controls, such as recombinant cathepsin D may be used. Negative controls can also be used, namely cathepsin D knock-out/down samples or specific inhibitors like pepstatin A. This substrate is also cleaved by the less abundant cathepsin E and therefore manufacturer instructions recommend the use of the *Ascaris* pepsin inhibitor.

## *In situ* labelling with Magic Red cathepsin B/L kits

Cathepsin B and L are both lysosomal cysteine proteases and their *in situ* activity can be monitored within living cells using commercially available kits. These assays are based on the generation of cresyl violet fluorophores upon enzymatic cleavage of peptide substrates. The substrates are based on the preference of the proteases to cleave specific amino-acid sequences but they do not guarantee absolute specificity. Fluorescent dextran is used as a control for the normalization of the fluorescent signal generated within lysosomes.

**REAGENTS**

- Alexa Fluor 647dextran (Invitrogen, D22914)
- Magic Red cathepsin B/L substrate (ImmunoChemistry Technologies, ICT-938/942)

**METHOD**

- Seed cells in a Nunc Lab-Tek Chambered Coverglass (ThermoFisher, 155411).
- Label lysosomes by incubating cells overnight with 50 μg/ml dextran - Alexa Fluor 647 followed by a chase of 3 h.
- Incubate the cells with Magic Red cathepsin B/L substrate for 15 min, wash and image.

Cathepsin B/L activity is assessed by normalizing the Magic Red to dextran fluorescent signal. As negative controls, 100 nM bafilomycin (vacuolar-type H^+^-ATPase inhibitor or 25 µM of the cysteine protease inhibitor leupeptin) can be added 1 h prior to Magic Red.

## LAMP1 cell surface detection by flow cytometry

When late endosomes/lysosomes fuse with the plasma membrane, epitopes present on the luminal domain of the lysosomal membrane proteins are exposed. Thus, lysosomal proteins like LAMP1 can be detected at the plasma membrane and provide a read-out for lysosome exocytosis. Flow cytometry is a quantitative method to monitor the presence of LAMP1 at the plasma membrane. This technique can be performed in adherent or suspension cells and allows the analysis of a high number of cells in a short period of time.

**CELL LINES**

- HeLa human cervical cancer cell line

**REAGENTS**

- Anti-human LAMP1 (CD107a) luminal epitope antibody, clone H4A3, Alexa Fluor 488 (BioLegend, 328610)
- Propidium iodide (PI, Invitrogen, P3566) 1 mg/ml
- FACS buffer (1% FBS, 2 mM EDTA in PBS)
- HBSS [-]CaCl_2_, [-]MgCl_2_ (Gibco, LTID 14175-053)
- Ionomycin from *Streptomyces conglobatus* (Sigma, I9657)
- 0.1 M CaCl_2_ (Sigma, 21115) diluted in HBSS

**METHOD**

- Seed HeLa cells in complete medium, in 24-well plates. Cells should be 80-90% confluent at the time of the assay.
- Remove medium and incubate cells with ice-cold HBSS and 10 µM ionomycin in the presence of 4 mM CaCl_2_, for 10 min at 37°C, to trigger lysosome exocytosis. Cells incubated with HBSS alone are used as control.
- Place cells immediately on ice and collect them in 500 μl of ice-cold FACS buffer by pipetting up and down to resuspend cells. Transfer the cells to an eppendorf tube.
- Centrifuge the cells at 300 x*g* for 5 min at 4°C.
- Wash the cells 2x with FACS buffer.
- Incubate cells with 50 μl of anti-LAMP1 Alexa Fluor 488 antibody in FACS buffer (1:1,000), for 30 min on ice. Protect the cells from the light to avoid fluorescence loss.
- Wash the cells 2x with FACS buffer and then resuspend the cell pellet in 200 μl FACS buffer. Transfer the cell suspension to flow cytometry tubes.
- Add to each sample 100 μl of 1.5 μg/ml PI (final concentration 0.5 μg/ml), immediately before analysis.
- Calculate the percentage of LAMP1-positive/PI-negative cells or mean intensity fluorescence (MFI) of LAMP1 detected in PI-negative cells.

## β-hexosaminidase release assay to assess lysosome exocytosis

One of the most common methods to assess lysosome exocytosis is to measure the activity of released lysosomal hydrolytic enzymes such as β-hexosaminidase, in the extracellular milieu. This method uses a specific substrate that becomes fluorescent upon cleavage.

**CELL LINES**

- HeLa human cervical cancer cell line

**REAGENTS**

- β-hexosaminidase substrate (4-methyl-umbelliferyl-N-acetyl-β-d-glucosaminide, Glycosynth, 44007)
- Substrate buffer (40 mM sodium citrate, 88 mM Na_2_PO_4_ pH 4.5)
- Micro BCA protein assay kit (Pierce, 23235)
- FACS buffer (1% FBS and 2 mM EDTA in PBS)
- 1 % IGEPAL CA-630 (Sigma, I8896)
- HBSS [-]CaCl_2_, [-]MgCl_2_ (Gibco, LTID 14175-053)
- Ionomycin from Streptomyces conglobatus (Sigma, I9657)
- 0.1 M CaCl_2_ (Sigma, 21115) in HBSS

**METHOD**

- Seed HeLa cells in complete medium, in 24-well plates. Cells should be 80-90% confluent at the time of the assay.
- Remove medium and incubate cells with ice-cold HBSS and 10 µM ionomycin in the presence of 4 mM CaCl_2_, for 10 min at 37°C, to trigger lysosome exocytosis. Cells incubated with HBSS alone are used as control.
- Place cells immediately on ice. Collect supernatant, containing the released β-hexosaminidase, to an eppendorf tube and store on ice.
- Lyse cells in 500 µl of 1% IGEPAL in dH_2_O and transfer cell lysate to an Eppendorf tube. Store on ice.
- Centrifuge cell supernatants and cell lysates for 5 min at 11,000 x*g*, at 4°C. Collect supernatants and discard the pellets.
- Supernatants should be used directly and cell lysates can be diluted 1:5 in dH_2_O. HBSS and 5x diluted 1% IGEPAL should be used as controls to subtract the background.
- Distribute 100 µl of sample: supernatant and diluted cell lysate in a black 96-well plate (Greiner, 655209) to measure β-hexosaminidase activity, and a standard 96-well plate (Costar, 3370), for protein quantification, in duplicate.
- To measure β-hexosaminidase activity, add to each well 100 µl of 6 mM 4-methyl-umbelliferyl-N-acetyl-β-d-glucosaminide, previously diluted in substrate buffer and filtered, and incubate for 15 min at 37°C.
- Measure fluorescence at 365 nm (excitation) and 450 nm (emission).
- For protein quantification, add to each well 100 µl of micro BCA protein reagent kit, as described by the manufacturer. A BSA calibration curve must be performed. Incubate for 30 min at 37°C. Measure absorbance at 560 nm.
- β-hexosaminidase (β-hex) activity can be calculated for each sample normalizing to total protein amount as following: β-hex activity in supernatant = [fluorescence (365/450) - HBSS alone]/protein µg. β-hex activity in cell lysate = [fluorescence (365/450) – lysis buffer alone]/protein µg. Total β-hex activity = β-hex activity in supernatant + 5x β-hex activity in cell lysate. Finally, the percentage of β-hex secretion is calculated as following: β-hex secretion (% of total) = 100 x (β-hex activity in the supernatant/total β-hex activity).

## Measurement of β-hexosaminidase activity

The glycosidase β-hexosaminidase is one of the most abundant lysosomal hydrolases. Its activity is often used to monitor lysosomal biogenesis and exocytosis. This assay allows the determination of the enzymatic activity in cellular and tissue lysates (as well as in other biological samples) following the cleavage of the fluorescent group 4-Methylumbelliferone (4-MU) in a multiwell format.

**REAGENTS**

- Lysis buffer (PBS 25 mM pH 6.5, 0.1% Triton X-100 and protease inhibitors)
- Reaction buffer [McIlvaine buffer (150 mM citrate-Na_2_HPO_4_, pH 4.0), 0.1% BSA]
- Substrate 4-methylumbelliferone-N-acetyl-β-D-glucosaminide (Sigma, 69585)
- Stop buffer (1 M NaOH-glycine pH 10.3)
- 4-MU (Sigma, M1381)

**METHOD**

- Lyse the cells or tissues in lysis buffer.
- Dilute the sample in McIlvaine buffer to a final volume of 25 µl. Protein input will depend on the relative abundance of the enzyme in the biological sample, typically between 1-10 µg of protein.
- Pipette the samples and controls (25 µl) into a 96-well plate (Costar 96-Well Black Polystyrene Plate, 734-1663).
- Incubate the samples with 100 µl of 1.97 mM substrate diluted in reaction buffer.
- After 15-30 min, stop the reaction with 200 µl of stop buffer.
- Measure fluorescence at 366 nm (excitation) and 445 nm (emission).
- Subtract background values (without the biological sample) to measured activities.
- To calculate the relative enzymatic activity, normalize the fluorescence to the protein content and to the control values. For determination of specific enzymatic activity (nmol/µg/h), a 4-MU calibration curve should be done (*e.g*., 0.1 – 10 nmol) in order to determine the fluorescence emitted by 1 nmol of free 4-MU.

Positive controls such as recombinant β-hexosaminidase may be used. Negative controls can also be used, namely *HEXB* knock-out/down samples or specific inhibitors such as iminocyclitol [55].

# **References**

1. Willems LI, Beenakker TJM, Murray B, Scheij S, Kallemeijn WW, Boot RG, Verhoek M, Donker-Koopman WE, Ferraz MJ, Van Rijssel ER, Florea BI, Codée JDC, Van Der Marel GA, Aerts JMFG, Overkleeft HS. Potent and selective activity-based probes for GH27 human retaining $α$-galactosidases. J Am Chem Soc. 2014;136:11622–11625.

2. Jiang J, Kallemeijn WW, Wright DW, Van Den Nieuwendijk AMCH, Rohde VC, Folch EC, Van Den Elst H, Florea BI, Scheij S, Donker-Koopman WE, Verhoek M, Li N, Schürmann M, Mink D, Boot RG, Codée JDC, Van Der Marel GA, Davies GJ, Aerts JMFG, Overkleeft HS. In vitro and in vivo comparative and competitive activity-based protein profiling of GH29 α-L-fucosidases. Chem Sci 2015;6:2782–2789.

3. Artola M, Kuo CL, McMahon SA, Oehler V, Hansen T, van der Lienden M, He X, van den Elst H, Florea BI, Kermode AR, van der Marel GA, Gloster TM, Codée JDC, Overkleeft HS, Aerts JMFG. New Irreversible α-l-Iduronidase Inhibitors and Activity-Based Probes. Chem - A Eur J 2018;24:19081–19088.

4. Armstrong Z, Kuo CL, Lahav D, Liu B, Johnson R, Beenakker TJM, De Boer C, Wong CS, Van Rijssel ER, Debets MF, Florea BI, Hissink C, Boot RG, Geurink PP, Ovaa H, Van Der Stelt M, Van Der Marel GM, Codée JDC, Aerts JMFG, Wu L, Overkleeft HS, Davies GJ. Manno- epi-cyclophellitols Enable Activity-Based Protein Profiling of Human $α$-Mannosidases and Discovery of New Golgi Mannosidase II Inhibitors. J Am Chem Soc. 2020;142:13021–13029.

5. Jiang J, Kuo CL, Wu L, Franke C, Kallemeijn WW, Florea BI, Van Meel E, Van Der Marel GA, Codée JDC, Boot RG, Davies GJ, Overkleeft HS, Aerts JMFG. Detection of active mammalian GH31 α-glucosidases in health and disease using in-class, broad-spectrum activity-based probes. ACS Cent Sci 2016;2:351–358.

6. Willems LI, Beenakker TJM, Murray B, Scheij S, Kallemeijn WW, Boot RG, Verhoek M, Donker-Koopman WE, Ferraz MJ, Van Rijssel ER, Florea BI, Codée JDC, Van Der Marel GA, Aerts JMFG, Overkleeft HS. Potent and selective activity-based probes for GH27 human retaining α-galactosidases. J Am Chem Soc 2014;136:11622–11625.

7. Schröder SP, Van De Sande JW, Kallemeijn WW, Kuo CL, Artola M, Van Rooden EJ, Jiang J, Beenakker TJM, Florea BI, Offen WA, Davies GJ, Minnaard AJ, Aerts JMFG, Codée JDC, Van Der Marel GA, Overkleeft HS. Towards broad spectrum activity-based glycosidase probes: Synthesis and evaluation of deoxygenated cyclophellitol aziridines. Chem Commun. 2017;53:12528–12531.

8. Wu L, Jiang J, Jin Y, Kallemeijn WW, Kuo CL, Artola M, Dai W, Van Elk C, Van Eijk M, Van Der Marel GA, Codée JDC, Florea BI, Aerts JMFG, Overkleeft HS, Davies GJ. Activity-based probes for functional interrogation of retaining β-glucuronidases. Nat Chem Biol 2017;13:867–873.

9. Tanaka Y, Okuda S, Sawai A, Suzuki S. Development of a N-acetyl-β-D-glucosaminidase (NAG) assay on a centrifugal lab-on-a-compact-disc (Lab-CD) platform. Anal Sci 2012;28:33–38.

10. Marques ARA, Willems LI, Herrera Moro D, Florea BI, Scheij S, Ottenhoff R, van Roomen CPAA, Verhoek M, Nelson JK, Kallemeijn WW, Biela-Banas A, Martin OR, Cachón-González MB, Kim NN, Cox TM, Boot RG, Overkleeft HS, Aerts JMFG. A Specific Activity-Based Probe to Monitor Family GH59 Galactosylceramidase, the Enzyme Deficient in Krabbe Disease. ChemBioChem. 2017;18:402–412.

11. Witte MD, Kallemeijn WW, Aten J, Li KY, Strijland A, Donker-Koopman WE, Van Den Nieuwendijk AMCH, Bleijlevens B, Kramer G, Florea BI, Hooibrink B, Hollak CEM, Ottenhoff R, Boot RG, Van Der Marel GA, Overkleeft HS, Aerts JMFG. Ultrasensitive in situ visualization of active glucocerebrosidase molecules. Nat Chem Biol. 2010;6:907–913.

12. Cuddy LK, Mazzulli JR. Analysis of lysosomal hydrolase trafficking and activity in human iPSC-derived neuronal models. STAR Protoc 2021;2:100340.

13. Liu J, Schleyer KA, Bryan TL, Xie C, Seabra G, Xu Y, Kafle A, Cui C, Wang Y, Yin K, Fetrow B, Henderson PKP, Fatland PZ, Liu J, Li C, Guo H, Cui L. Ultrasensitive small molecule fluorogenic probe for human heparanase. Chem Sci. 2021;12:239–246.

14. Zhang LS, Mummert ME. Development of a fluorescent substrate to measure hyaluronidase activity. Anal Biochem. 2008;379:80–85.

15. Chib R, Mummert M, Bora I, Laursen BW, Shah S, Pendry R, Gryczynski I, Borejdo J, Gryczynski Z, Fudala R. Fluorescent biosensor for the detection of hyaluronidase: intensity-based ratiometric sensing and fluorescence lifetime-based sensing using a long lifetime azadioxatriangulenium (ADOTA) fluorophore. Anal Bioanal Chem. 2016;408:3811–3821.

16. Yuan L, Zhao Y, Sun XL. Sialidase substrates for Sialdiase assays - activity, specificity, quantification and inhibition. Glycoconj. J. 2020;37:513–531.

17. Minami A, Kurebayashi Y, Takahashi T, Otsubo T, Ikeda K, Suzuki T. The function of sialidase revealed by sialidase activity imaging probe. Int. J. Mol. Sci. 2021;22:1–14.

18. Schwaid AG, Ruangsiriluk W, Reyes AR, Cabral S, Rajamohan F, Tu M, Ward J, Carpino PA. Development of a selective activity-based probe for glycosylated LIPA. Bioorganic Med Chem Lett 2016;26:1993–1996.

19. Shayman JA, Tesmer JJG. Lysosomal phospholipase A2. Biochim. Biophys. Acta - Mol. Cell Biol. Lipids. 2019;1864:932–940.

20. Henneke M, Diekmann S, Ohlenbusch A, Kaiser J, Engelbrecht V, Kohlschütter A, Krätzner R, Madruga-Garrido M, Mayer M, Opitz L, Rodriguez D, Rüschendorf F, Schumacher J, Thiele H, Thoms S, Steinfeld R, Nürnberg P, Gärtner J. RNASET2-deficient cystic leukoencephalopathy resembles congenital cytomegalovirus brain infection. Nat Genet. 2009;41:773–775.

21. Kawane K, Nagata S. Chapter Fourteen Nucleases in Programmed Cell Death. Methods Enzymol. 2008;442:271–287.

22. Cappel C, Gonzalez AC, Damme M. Quantification and characterization of the 50 exonuclease activity of the lysosomal nuclease PLD3 by a novel cell-based assay. J Biol Chem. 2021;296.

23. Van Noorden CJF. Imaging enzymes at work: Metabolic mapping by enzyme histochemistry. J. Histochem. Cytochem. 2010;58:481–497.

24. Greenbaum D, Baruch A, Hayrapetian L, Darula Z, Burlingame A, Medzihradszky KF, Bogyo M. Chemical approaches for functionally probing the proteome. Mol Cell Proteomics 2002;1:60–68.

25. Verdoes M, Oresic Bender K, Segal E, Van Der Linden WA, Syed S, Withana NP, Sanman LE, Bogyo M. Improved quenched fluorescent probe for imaging of cysteine cathepsin activity. J Am Chem Soc. 2013;135:14726–14730.

26. Poreba M, Groborz K, Vizovisek M, Maruggi M, Turk D, Turk B, Powis G, Drag M, Salvesen GS. Fluorescent probes towards selective cathepsin B detection and visualization in cancer cells and patient samples. Chem Sci. 2019;10:8461–8477.

27. Yuan F, Verhelst SHL, Blum G, Coussens LM, Bogyo M. A selective activity-based probe for the papain family cysteine protease dipeptidyl peptidase I/cathepsin C. J Am Chem Soc. 2006;128:5616–5617.

28. Kozloff KM, Quinti L, Patntirapong S, Hauschka P V, Tung CH, Weissleder R, Mahmood U. Non-invasive optical detection of cathepsin K-mediated fluorescence reveals osteoclast activity in vitro and in vivo. Bone. 2009;44:190–198.

29. Frizler M, Yampolsky I V., Baranov MS, Stirnberg M, Gütschow M. Chemical introduction of the green fluorescence: Imaging of cysteine cathepsins by an irreversibly locked GFP fluorophore. Org Biomol Chem 2013;11:5913–5921.

30. Dana D, Garcia J, Bhuiyan AI, Rathod P, Joo L, Novoa DA, Paroly S, Fath KR, Chang EJ, Pathak SK. Cell penetrable, clickable and tagless activity-based probe of human cathepsin L. Bioorg Chem 2019;85:505–514.

31. Poreba M, Rut W, Vizovisek M, Groborz K, Kasperkiewicz P, Finlay D, Vuori K, Turk D, Turk B, Salvesen GS, Drag M. Selective imaging of cathepsin L in breast cancer by fluorescent activity-based probes. Chem Sci. 2018;9:2113–2129.

32. Mertens MD, Schmitz J, Horn M, Furtmann N, Bajorath J, Mareš M, Gütschow M. A coumarin-labeled vinyl sulfone as tripeptidomimetic activity-based probe for cysteine cathepsins. ChemBioChem. 2014;15:955–959.

33. Oresic Bender K, Ofori L, Van Der Linden WA, Mock ED, Datta GK, Chowdhury S, Li H, Segal E, Sanchez Lopez M, Ellman JA, Figdor CG, Bogyo M, Verdoes M. Design of a Highly Selective Quenched Activity-Based Probe and Its Application in Dual Color Imaging Studies of Cathepsin S Activity Localization. J Am Chem Soc. 2015;137:4771–4777.

34. Paulick MG, Bogyo M. Development of activity-based probes for cathepsin X. In: ACS Chemical Biology. American Chemical Society; 2011. p. 563–572.

35. Lee J, Bogyo M. Development of near-infrared fluorophore (NIRF)-labeled activity-based probes for in vivo imaging of legumain. ACS Chem Biol. 2010;5:233–243.

36. Hartley DM, Snodgrass SR, Bradshaw PA. The Measurement of Gamma-Glutamyl Hydrolase (Conjugase) Activity in Rat Brain. 1988.

37. Kasperkiewicz P, Altman Y, D’Angelo M, Salvesen GS, Drag M. Toolbox of Fluorescent Probes for Parallel Imaging Reveals Uneven Location of Serine Proteases in Neutrophils. J Am Chem Soc. 2017;139:10115–10125.

38. Nussbaumerová M, Srp J, Máša M, Hradilek M, Šanda M, Reiniš M, Horn M, Mareš M. Single- and double-headed chemical probes for detection of active cathepsin D in a cancer cell proteome. ChemBioChem. 2010;11:1538–1541.

39. Schauer-Vukasinovic V, Bur D, Kitas E, Schlatter D, Rossé G, Lahm HW, Giller T. Purification and characterization of active recombinant human napsin A. Eur J Biochem. 2000;267:2573–2580.

40. Dubois G, Turpin J, Baumann N. P-Nitrocatechol sulfate for arylsulfatase assay: detection of metachromatic leukodystrophy variants. Adv Exp Med Biol. 1976;68:233–237.

41. Kumar AB, Spacil Z, Ghomashchi F, Masi S, Sumida T, Ito M, Turecek F, Scott CR, Gelb MH. Fluorimetric assays for N-acetylgalactosamine-6-sulfatase and arylsulfatase B based on the natural substrates for confirmation of mucopolysaccharidoses types IVA and VI. Clin Chim Acta 2015;451:125–128.

42. Armand Cognetta III AB, Niphakis MJ, Lee H-C, Martini ML, Hulce JJ. Selective N-Hydroxyhydantoin Carbamate Inhibitors of Mammalian Serine Hydrolases. Chem Biol. 2015;22:928–937.

43. Bedia C, Camacho L, Abad JL, Fabriàs G, Levade T. A simple fluorogenic method for determination of acid ceramidase activity and diagnosis of Farber disease. J Lipid Res. 2010;51:3542–3547.

44. Ouairy CMJ, Ferraz MJ, Boot RG, Baggelaar MP, Van Der Stelt M, Appelman M, Van Der Marel GA, Florea BI, Aerts JMFG, Overkleeft HS. Development of an acid ceramidase activity-based probe. Chem Commun. 2015;51:6161–6163.

45. Ordóñez YF, Abad JL, Aseeri M, Casas J, Garcia V, Casasampere M, Schuchman EH, Levade T, Delgado A, Triola G, Fabrias G. Activity-Based Imaging of Acid Ceramidase in Living Cells. J Am Chem Soc. 2019;141:7736–7742.

46. Cai S, Liu C, Jiao X, He S, Zhao L, Zeng X. A lysosome-targeted near-infrared fluorescent probe for imaging of acid phosphatase in living cells. Org Biomol Chem. 2020;18:1148–1154.

47. Choi Y, Tuzikov AB, Ovchinnikova T V, Bovin N V, Pshezhetsky A V. Novel direct assay for acetyl-CoA:$α$-Glucosaminide N-acetyltransferase using BODIPY-glucosamine as a substrate In: JIMD Reports. Springer; 2016. p. 11–18. Available from: http://link.springer.com/10.1007/8904_2015_501

48. Carbonnelle-Puscian A, Copie-Bergman C, Baia M, Martin-Garcia N, Allory Y, Haioun C, Crémades A, Abd-Alsamad I, Farcet J-P, Gaulard P, Castellano F, Molinier-Frenkel V. The novel immunosuppressive enzyme IL4I1 is expressed by neoplastic cells of several B-cell lymphomas and by tumor-associated macrophages. Leukemia. 2009;23:952–960.

49. Mühle C, Huttner HB, Walter S, Reichel M, Canneva F, Lewczuk P, Gulbins E, Kornhuber J. Characterization of Acid Sphingomyelinase Activity in Human Cerebrospinal Fluid. PLoS One. 2013;8.

50. van Diggelen OP, Voznyi Y V, Keulemans JLM, Schoonderwoerd K, Ledvinova J, Mengel E, Zschiesche M, Santer R, Harzer K. A new fluorimetric enzyme assay for the diagnosis of Niemann-Pick A/B, with specificity of natural sphingomyelinase substrate. J Inherit Metab Dis 2005;28:733–741.

51. Arunachalam B, Phan UT, Geuze HJ, Cresswell P. Enzymatic reduction of disulfide bonds in lysosomes: Characterization of a gamma-interferon-inducible lysosomal thiol reductase (GILT). Proc Natl Acad Sci U S A [Internet] 2000;97:745–750.

52. Soldati C, Lopez-Fabuel I, Wanderlingh LG, Garcia-Macia M, Monfregola J, Esposito A, Napolitano G, Guevara-Ferrer M, Scotto Rosato A, Krogsaeter EK, Paquet D, Grimm CM, Montefusco S, Braulke T, Storch S, Mole SE, De Matteis MA, Ballabio A, Sampaio JL, McKay T, Johannes L, Bolaños JP, Medina DL. Repurposing of tamoxifen ameliorates CLN3 and CLN7 disease phenotype. EMBO Mol Med. 2021; 13(10):e13742.

53. King KB, Kimura JH. The establishment and characterization of an immortal cell line with a stable chondrocytic phenotype. J Cell Biochem. 2003;(5):992-1004.

54. Rappsilber J, Ishihama Y, Mann M. Stop and go extraction tips for matrix-assisted laser desorption/ionization, nanoelectrospray, and LC/MS sample pretreatment in proteomics. Anal Chem. 2003; 75(3):663-70.

55. Liu J, Shikhman AR, Lotz MK, Wong CH. Hexosaminidase inhibitors as new drug candidates for the therapy of osteoarthritis. Chem Biol 2001;8:701–711.

56. Churamani D, Hooper R, Rahman T, Brailoiu E, Patel S. The N-terminal region of two-pore channel 1 regulates trafficking and activation by NAADP. Biochem J. 2013;453(1):147-51.

57. Davis LC, Morgan AJ, Galione A. NAADP ‐regulated two‐pore channels drive phagocytosis through endo‐lysosomal Ca 2+ nanodomains, calcineurin and dynamin . EMBO J 2020;39:1–23.

58. Zhao Y, Araki S, Wu J, Teramoto T, Chang YF, Nakano M, Abdelfattah AS, Fujiwara M, Ishihara T, Nagai T, Campbell RE. An expanded palette of genetically encoded Ca²⁺ indicators. Science. 2011;333(6051):1888-91.

59. Davis LC, Morgan AJ, Galione A. NAADP-regulated two-pore channels drive phagocytosis through endo-lysosomal Ca2+ nanodomains, calcineurin and dynamin. EMBO J. 2020;39(14):e104058.

60. Wang X, Zhang X, Dong XP, Samie M, Li X, Cheng X, Goschka A, Shen D, Zhou Y, Harlow J, Zhu MX, Clapham DE, Ren D, Xu H. TPC proteins are phosphoinositide- activated sodium-selective ion channels in endosomes and lysosomes. Cell. 2012;151(2):372-83.
